# *In silico* analysis reveals a multi-dimensional model of adaptive evolution in the flax orbitide-related precursor protein family

**DOI:** 10.3389/fpls.2026.1824173

**Published:** 2026-06-30

**Authors:** Ziliang Song, Timothy F. Sharbel, Yong Wang, Martin J. T. Reaney

**Affiliations:** 1 China-Malaysia Belt and Road Joint Laboratory on Oil Processing and Safety, Jinan University, Guangzhou, Guangdong, China; 2 Department of Plant Sciences, College of Agriculture and Bioresources, University of Saskatchewan, Saskatoon, SK, Canada; 3 Department of Food and Bioproduct Sciences, College of Agriculture and Bioresources, University of Saskatchewan, Saskatoon, SK, Canada

**Keywords:** adaptive evolution, duplication, flax, linusorb, repeat, selection

## Abstract

Flaxseed is abundant in cyclic peptides called linusorbs, which are derived from precursor proteins through post-translational modification. Previous studies have shown that four of the five precursor proteins contain repeat structures, where the variable linusorb domain is flanked by conserved signatures. The genome of flax cultivar CDC Bethune encodes 25 additional proteins which share the linusorbs-containing repeat pattern. Gene sequence analysis revealed that these repeats have arisen through three distinct modes of tandem duplication. Driven by the genetic diversity of potential linusorbs, here we continued to characterize these linusorb-related tandem repeats (LRTRs) at the amino acid level. Similar to known linusorbs, the linusorb-like domains (LLDs) are moderately hydrophobic in contrast to the hydrophilic spacers. The conserved flanking signatures of known linusorbs remain dominant among the LLDs, indicating a conserved role in the 30 proteins. Similarities of repeat pairs (RPs) across paralogous proteins are at the level between those within proteins and those across non-paralogues, suggesting the LRTRs in protein paralogues underwent adaptive evolution after ancestral gene duplication. Positive selection was identified to episodically act on certain sites and lineages of LRTRs both within and across paralogues. By combining all the evidence, we proposed a multi-dimensional model of adaptive evolution in cyclic peptide precursor proteins for the first time, which involves the concomitance of (a) protein paralogues divergence, (b) repeat divergence within protein paralogues and (c) repeat divergence across protein paralogues. Based on a single cultivated species, the proposed model serves as a prototype for broader evolutionary studies across other Linum species.

## Introduction

Plants synthesize peptides for various purposes, including growth regulation (Shinohara, 2021), reproductive development (Stintzi and Schaller, 2022), signaling (Hirakawa and Sawa, 2019; Sun and Zhang, 2021) and defense against biotic/abiotic stress (Goyal and Mattoo, 2014; Campos et al., 2018). Many of these peptides are utilized in food and pharmaceutical industries because of their bioactivities and health benefits (Lima et al., 2022; Yuan et al., 2022). One unique class of plant peptides is cyclic peptides (CPs), which can be synthesized through N-to-C cyclization of a precursor protein by proteolytic enzymes (Arnison et al., 2013). Depending on the number of disulfide bonds in the peptide ring, there are three structural classes of CPs: cyclotides (Hirakawa and Sawa, 2019), PawS-derived peptides (1) and orbitides (0). Over the past decade, the discovery of plant-derived CPs has been driven by omics approaches (Hellinger et al., 2015; Fisher et al., 2020; Kalmankar et al., 2020). *In silico* mining facilitates the identification of both peptides and their precursor proteins, providing an efficient complement to traditional mass spectrometry methods (Koehbach and Clark, 2016).

**Figure 1:**
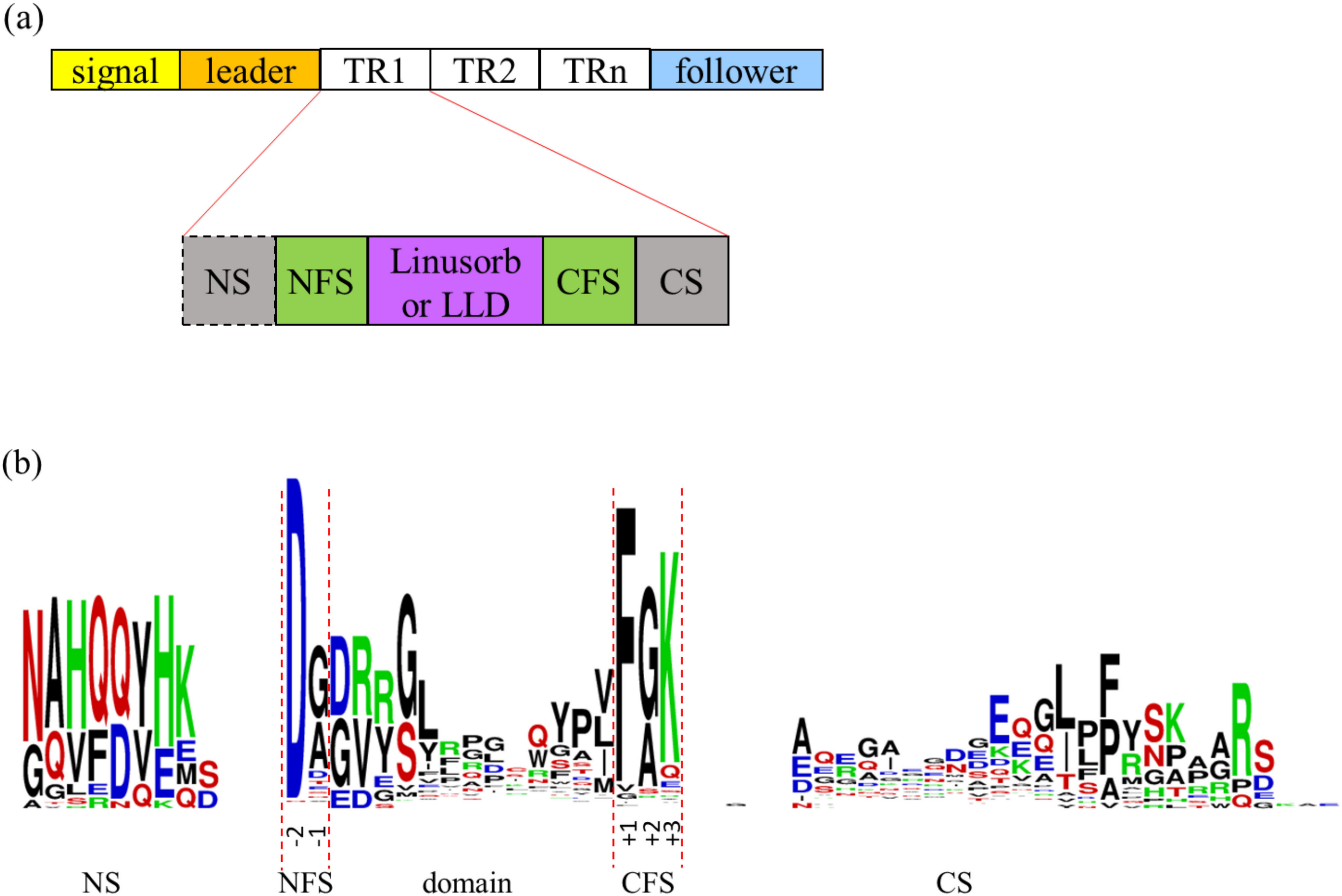
**(a)** Graphical structures of a linusorb-related protein (top) and its TR unit (bottom). The protein contains an N-terminal signal sequence, a leader peptide, a multiple-TR region and a C-terminal follower sequence. A TR comprises 5 sections: N-terminal spacer (NS), N-flanking signature (NFS), a linusorb or linusorb-like domain (LLD), C-flanking signature (CFS) and C-terminal spacer (CS). If the sequence N-terminal to the NFS of the first LLD does not match the consensus TR pattern, these TRs do not have the NS (represented by dashed block). **(b)** Sequence logo of the aligned 336 LRTRs, dissected into the above 5 sections. Created by WebLogo: https://weblogo.berkeley.edu/.

Among the CPs discovered in plants, orbitides naturally occurring in cultivated flax (*Linum usitatissimum*), also known as linusorbs, are one of the few arranged in repetitive structures in their precursor sequences. There are 11 unique linusorb domains distributed in five precursor proteins, four of which contain multiple tandem repeats (TRs), each embedded with one linusorb domain or linusorb-like domain (LLD) (Song et al., 2022). While the linusorb domains are highly diverse, alignment of the linusorb-embedded TRs demonstrated conservation in 5 flanking sites (2 N-terminal and 3 C-terminal, **Figure 1a**).

Using the 5 flanking signatures and the repeat pattern as search condition, we identified 281 LLD-containing repeats (LLDRs) in 25 putative proteins, revealing the diversity of potential orbitides encoded in the genome of flax cultivar CDC Bethune (Song et al., 2022). This profile-based mining scheme identified not only proteins that share little similarity but also proteins with varying copy numbers of TRs that often cause inaccurate alignment. As these 30 proteins, i.e. 25 LLDR-containing proteins and 5 linusorb precursor proteins, are rich in repeats, we then verified their gene sequences through Sanger sequencing (Song et al., 2025). The 30 gene sequences were published in the GenBank accessions OP006678 – OP006707, with the coding sequences (CDSs) and protein sequences annotated in the gene features. Pairwise similarity analysis followed by hierarchical clustering identified 8 groups of paralogues among 21 of these 30 genes (**Table 1**). Based on the pairwise alignments of TRs, we identified 3 modes of duplication that differ in the number of TRs as one duplication unit, primarily inferring TR evolution at the nucleotide level (Song et al., 2025). Alignments of the protein paralogues are displayed in **Supplementary Data Sheet S1**.

**Table 1:** Eight groups of paralogues among 21 genes, adapted from (Song et al., 2025).

Group	Gene 1	Gene 2	Gene 3	Gene 4
1	G11-516P	G11-514P	G14-170N	
2	G3-449N	G4-136N		
3	Lu2-51734	Lu13-23576		
4	Lu5-45630	Lu8-3470		
5	Lu5-46938	Lu8-2811		
6	Lu6-41637	Lu12-11761	Lu12-11698	Lu14-5765
7	Lu10-34966	Lu11-24918	Lu11-28070	
8	Lu9-15288	Lu10-38024	Lu10-38063	

Besides the previously identified 281 LLDRs, our preliminary analysis of the 25 proteins using RADAR (Heger and Holm, 2000) detected more TRs interspersed with LLDRs. These TRs share similar sequence architecture with LLDRs, each containing a domain flanked by signatures different from the flanking signatures of known linusorbs and thus not identified as LLD by previous proteome mining. Nevertheless, sequence similarity and repetitiveness indicate that these TRs are variant forms of LLDRs diversifying from TR duplication.

To further analyze the evolution of the linusorb-related gene family at the amino acid level, here we combined linusorb-embedded repeats, LLDRs and their variants into a linusorb-related tandem repeat (LRTR) family. There are totally 336 LRTRs distributed in 30 proteins. For subsequent alignment, each LRTR was dissected into 5 sections as illustrated in **Figure 1b**. The CDSs encoding LRTRs were also dissected in the same manner. **Supplementary Table 1** lists the 336 dissected LRTRs. Then we thoroughly characterized different sections of TRs to assess their conservation and divergence. Selection analysis was performed to delineate LRTR evolution. We also scanned the 30 proteins for their possible destinations in the cell to capture a glimpse of orbitide biosynthesis in flax. The evolutionary model of linusorb-related gene paralogues within a single genome provides a prototype for a broader study of orthologues across other Linum species.

## Materials and methods

### Pairwise comparison of LRTRs within and across paralogous proteins

We previously identified 8 groups of paralogues among 21 of the 30 genes and examined their repeat duplication at the DNA level (Song et al., 2025). For this study we applied the same pairwise comparison scheme to the LRTRs at the amino acid level, both within and across the paralogues. Briefly, a BioPython script aligned the LRTRs in a pairwise manner using the PAM30 substitution matrix, with gap penalties of 9 for opening and 1 for extension. The output alignment scores were sorted and filtered to generate data subsets for the 8 groups of paralogues. The similarity was calculated by taking the ratio of the alignment score to the self-alignment score of the shorter sequence between the two aligned sequences. This can normalize the alignment score to prevent it from being dependent on sequence length, so that the similarities of any sequence pairs, regardless of length, can be compared at the same level. A similarity matrix was constructed using the R package *pheatmap*, and a coloring scheme was applied using 50% similarity as the significance cutoff.

### Multiple sequence alignment of LRTRs and their CDSs

As LRTRs share a common structure consisting of five sections (**Figure 1**), the five sections were aligned individually by MUSCLE 3.8.31 with default settings (Edgar, 2004). The resulting alignments were then concatenated in the original order to generate the alignment of the entire LRTR. LRTR alignments were performed within each protein and across protein paralogues. PAL2NAL (Suyama et al., 2006) was used to align the CDSs based on the LRTR alignment. Additionally, alignments were conducted on the flanking non-TR sequences among paralogous genes. As TRs were detected by RADAR, the flanking non-TR sequences refer to both N-terminal and C-terminal sequences flanking the TR region in the protein, respectively.

### Phylogenetic analysis of LRTRs within and across paralogues

We investigated the homology of LRTRs within genes, including linusorb-containing repeats and LLDRs, by constructing a maximum-likelihood (ML) phylogenetic tree. The ML tree was built on codon alignment using the IQ-TREE webserver (Trifinopoulos et al., 2016). The substitution model CODON1 for standard codons was applied, and the substitution rate was automatically selected by the built-in ModelFinder. FreeRate heterogeneity was included in the model selection process, and we conducted an Ultrafast bootstrap test with 1000 alignments for branch support analysis. To examine phylogenetic relationships among the previously identified 8 groups of gene paralogues (Song et al., 2025), we also constructed ML trees for LRTRs and their flanking non-TR sequences using the same parameters as above.

### Pairwise calculation of Ka/Ks ratios between LRTRs within and across paralogous genes

The ratio of nonsynonymous (Ka) to synonymous (Ks) mutation rates, known as Ka/Ks or ω, was determined between LRTRs using the Yang & Nielsen method (Yang and Nielsen, 2000). This calculation was performed using the program YN00 in the PAML package version X (Xu and Yang, 2013). For each group of gene paralogues, the multiple sequence alignment of LRTR-encoding CDSs was used as input. The program estimated a common transition/transversion rate ratio (κ) for all sequences, estimated codon frequencies (F3×4 model) for each sequence pair, and applied equal weighting in the counting differences between codons. The output Ka/Ks ratios for all repeat pairs (RPs) were sorted into two data series: within each gene paralogue and across paralogues. Each data series was further sorted into two subsets: Ks >0 and Ks =0. For the Ks >0 subset, frequencies were calculated for ω=0, 0<ω<1, ω=1 and ω>1. For the Ks =0 subset, frequencies were calculated for Ka=0 and Ka>0. These frequencies were transformed into percentages of the total number of RPs series. Histograms were plotted to compare both RP series.

### Detection of adaptive evolution

To detect adaptive evolution acting on LRTRs, we estimated the selective pressure in the phylogeny of LRTRs. Purifying selection, neutral evolution and positive selection were indicated by 0< ω <1, ω =1 and ω >1, respectively. The ω ratio was estimated using the maximum-likelihood method (Yang and Nielsen, 2000) with the program CodeML in PAML X. The multiple CDS alignment and the derived ML tree were used as inputs. We chose the F3×4 codon substitution model to estimate the equilibrium codon frequency. We fixed the shape parameter α at 0 and set ncatG to 5 by default. We implemented the following 2 codon-based models, each with a specific assumption on how the ω ratio varies across the sequence or the branches in the phylogeny.

To identify positively selected sites in the alignment of LRTRs among gene paralogues and detect adaptive evolution acted on LRTRs, we employed 5 site models that assume a constant ω ratio across branches of the phylogeny but varies among sites (Yang and Nielsen, 2002). The 5 site models included M0 (one‐ratio, ω), M1a (nearly neutral, ω_0_<1, ω_1_ = 1), M2a (positive selection, ω_0_<1, ω_1_ = 1, ω_2_>1), M7 (beta) and M8 (beta and ω, ω_s_>1). CodeML performed two likelihood-ratio tests (LRTs) to compare M2a against M1a and M8 against M7, respectively. If the LRT yields a significant result for any of the site models, the posterior probabilities for site classes were calculated using the Bayes empirical Bayes (BEB) method (Yang et al., 2005). Sites with a posterior probability ≥ 95% were identified under positive selection.

Next, branch-site models were employed to detect positive selection that affects only certain branches or sites by allowing ω to vary both among branches in the phylogeny of LRTRs and among sites in the LRTRs. Foreground branches hypothesized to contain positively selected sites were specified, and the remaining branches were considered as background branches under either purifying selection or neutral evolution. The branch-site model A, which assumes the foreground branches contain positively selected sites (ω_2_>1), was compared with the corresponding null model where the foreground branches are under neutral or purifying selection (ω_2 ≤_ 1). Similarly, if the LRT was significant, the BEB method calculated posterior probabilities for site classes, and sites on the specified branch with a posterior probability ≥ 95% were identified under positive selection.

### Detection of gene conversion

Possible gene conversion events among paralogues were detected using the software GENECONV (Sawyer, 1989). Aligned coding sequences of paralogous proteins were loaded as input to the program. The option/r was entered to specify the inputs as coding sequences. The option/w123 was applied to perform permutations. The option/lp was entered to produce lists of pairwise significant fragments as well as global lists. For those paralogous groups containing only two genes, the option -Include_monosites was entered to include monomorphic sites in the alignment. The number of permutations (N) was set as 10,000 by default. Paralogue pairs or groups of multiple paralogues with significant fragments (pairwise or global *p*-value ≤ 0.05) were identified as gene conversion events.

## Results

### Copy number variation of linusorb domains and LLDs

**Figure 2:**
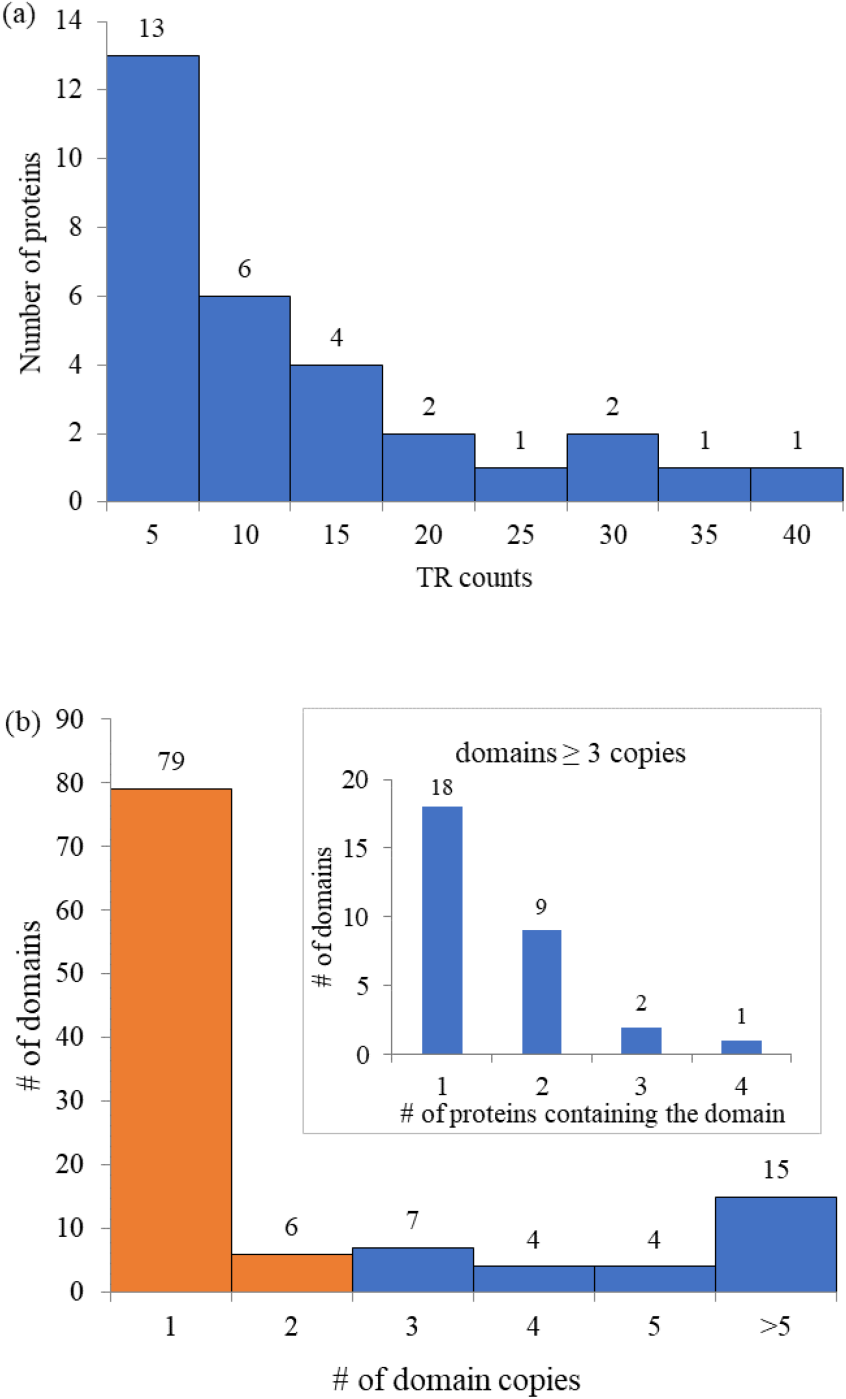
**(a)** Frequencies of proteins containing different ranges of LRTR counts; **(b)** frequencies of domains with different numbers of copies. Inset: frequencies of domains with ≥ 3 copies occurring in different numbers of proteins.

The distribution of LRTR numbers in the 30 proteins is displayed in **Figure 2a**. The number of LRTRs in a protein varies in a wide range from 1 to 37, with an average of 11 LRTRs per protein. More than half of the identified proteins contain 10 or fewer TRs while 5 proteins contain more than 20 TRs. Additionally, there is variation in the number of LRTRs across the 8 groups of paralogues. The standard deviations range from 0.7 to 15.1, and the coefficients of variation range from 0.09 to 0.71. A significantly positive correlation exists between the average number of LRTRs among the paralogues and the standard deviation, with the Pearson’s *r*(7) = 0.82, *p* < 0.05 computed from a two-tail *t*-test. This suggests that the rate of LRTR duplication within each paralogue varies, and the degree of variation increases with the average number of LRTRs.

Each LRTR contains one linusorb domain or LLD except for one empty case (Lu10-38063_2). Among the remaining 335 linusorb domains and LLDs, there are 115 unique sequences. The distribution of their copy numbers is shown in **Figure 2b**: 79 (69%) are singletons, 6 have 2 copies, and 30 have more than 3 copies. The most prevalent domain, GLRQGY, has 34 copies, followed by GLLRDY with 20 copies. While all the 6 domains with 2 copies have both copies embedded within one protein, the 30 domains with multiple copies can be found in different proteins. The inset of **Figure 2b** shows 18 domains have their multiple copies within one protein and 9 domains are shared by 2 proteins. Two domains, GYPPLSPL and GIRRTY, are found in 3 proteins. The second most frequently occurring domain, GLLRDY, is also most widely distributed, with its 20 copies present in 4 paralogous proteins (Group 6). We also found that all the proteins with common domains are paralogous, with Group 6 sharing 6 domains followed by Group 7 sharing 3 domains. This suggests these domains are most conserved among the paralogues.

### Linusorbs and LLDs exhibit physicochemical properties contrasting to spacers

**Figure 3:**
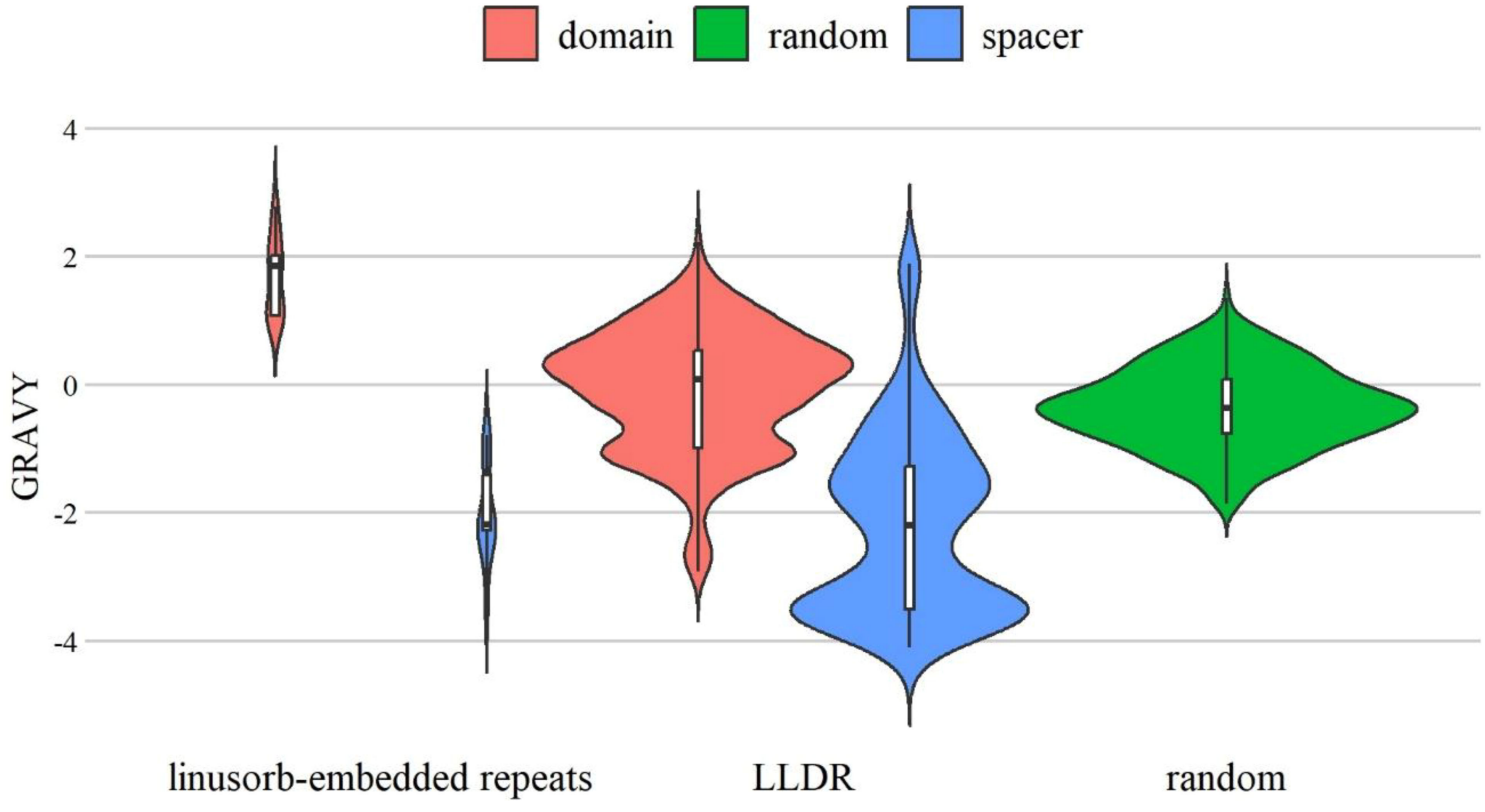
Violin plot illustrating the distributions of GRAVY values of domains and spacers in 15 linusorb-embedded repeats and 320 LLDRs, respectively. The plot includes a random group of 300 random sequences with a length of 10 amino acids at the frequencies of the flax proteome, created using the Sequence Manipulation Suite at https://www.bioinformatics.org/sms2/random_protein.html. The amino acid compositions of both the flax proteome and the 300 random sequences are presented in **Supplementary Data Sheet S3**. The 300 random sequences are displayed in **Supplementary Data Sheet S4**.

The grand average of hydropathy (GRAVY) (http://www.gravy-calculator.de/index.php) is positively correlated with the hydrophobicity of the molecule. The GRAVY values of the 8 known linusorb domains are all positive, ranging from 1.08 to 2.78, indicating their hydrophobicity. In contrast, the spacers in the linusorb-embedded repeats have negative GRAVY values ranging from -3.5 to -0.78. The linusorb domains are significantly more hydrophobic than their corresponding spacers with *p* < 0.05 in the Student’s *t*-test. The contrast between domains and spacers is also observed in LLDRs, as shown in **Figure 3**. Although LLDs are less hydrophobic than their 8 linusorb counterparts (*p* < 0.05), their distribution is more hydrophobic than the spacers in LLDRs (*p* < 0.05). This is evident from the fact that the highest kernel density of the domain category is above 0, compared to that of the spacer category below -2. Nonetheless, spacers of the linusorb domains and LLDs are not significantly different (*p*>0.05), indicating that spacers are consistently hydrophilic. All regions mentioned above are significantly different from random sequences (*p* < 0.05). The consistency in hydropathy between LLDs and linusorbs, as well as the contrast to the spacers, suggest the physicochemical property of the LRTRs is conserved across these proteins.

Note that the LLDs were previously identified based on criteria for conserved flanking signatures and domain length of linusorbs. The sequences of LLDs and spacers were intentionally left random, but their hydropathy distributions are different from that of random sequences. The LLDs have two kernel density peaks, the highest peak above 0 and the other below 0, whereas the random sequences have only one peak below 0 and exhibit a normal distribution. The majority of LLDR spacers have lower GRAVY values than random sequences, indicating that they are significantly hydrophilic.

**Figure 4:**
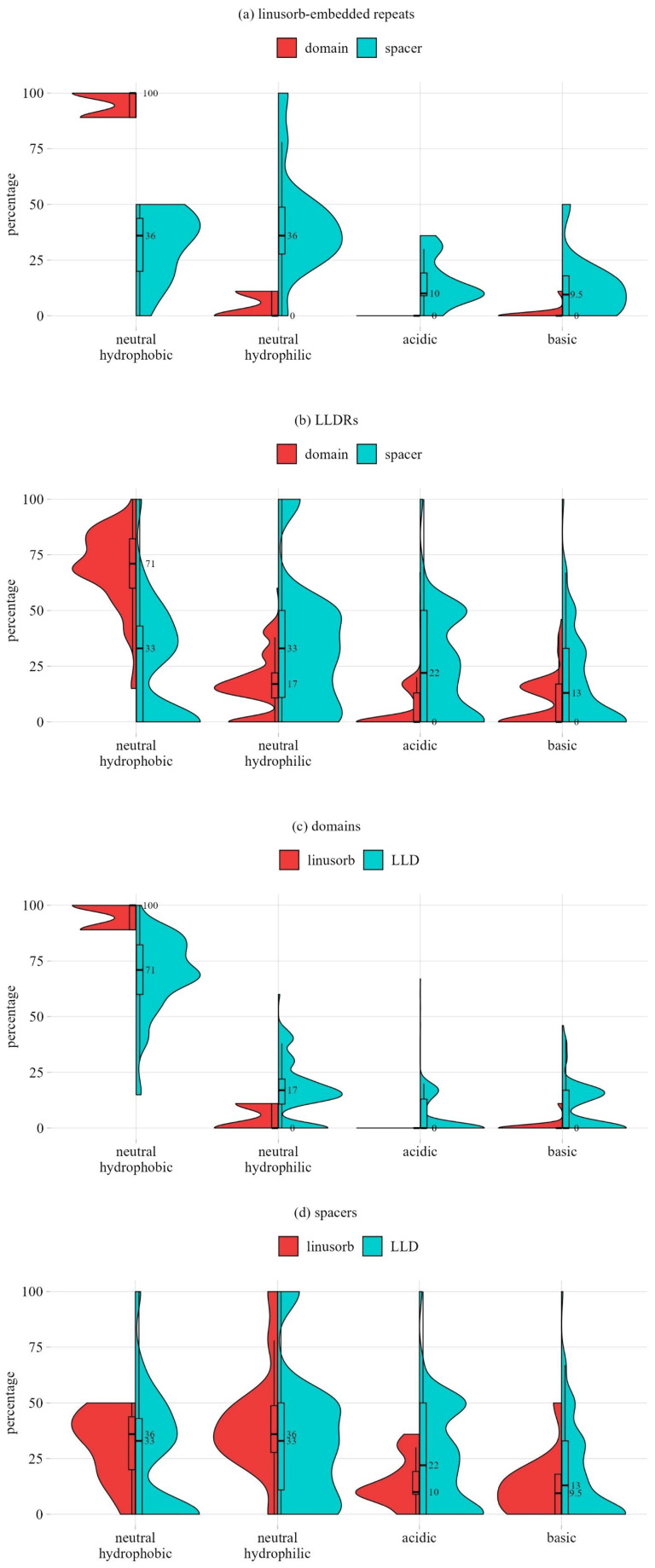
Split violin plots comparing the amino acid composition between domains (scarlet side) and spacers (cyan side) of **(a)** linusorb-embedded repeats and **(b)** LLDRs. Comparisons are also made between linusorbs and LLDs in terms of **(c)** domains and **(d)** their corresponding spacers. The percentage of each amino acid was calculated for the domain and the spacer of each LRTR and summed up to the corresponding category. The vertical boxes at the base of each violin represents the interquartile range (IQR) of the population. The thick horizontal bar inside each box and the number next to it represent the median.

There is also a distinct difference in the distributions of amino acid compositions between the domains and spacers, as shown by the different density areas between the left halves and the right halves of violin plots in **Figure 4**. In linusorb-embedded repeats (**Figure 4a**), the linusorb domains contain high percentages (89% – 100%) of neutral hydrophobic amino acids (GAPLIVMWFY) compared to ≤ 11% neutral hydrophilic amino acids (NQSTC), whereas the opposite proportions are observed in the spacers. Both acidic (DE) and basic (RHK) amino acids are rare in the linusorb-embedded repeats, but the linusorb domains are void of acidic amino acids. With respect to the individual amino acids (**Supplementary Table 2**), the most abundant amino acids in the linusorb domains on average are phenylalanine (F, 19.6%), leucine (L, 18.5%) and proline (P, 17.6%), all of which are neutral hydrophobic. In contrast, the most abundant amino acids in the spacers on average are glutamine (Q, 23.0%), alanine (A, 12.5%) and glutamic acid (E, 11.4%), each of which belongs to neutral hydrophilic, neutral hydrophobic and acidic, respectively.

In LLDRs (**Figure 4b**), the amino acid compositions of LLD domains are consistent with the linusorb-embedded repeats. Neutral hydrophobic amino acids still dominate the LLDs, albeit less extreme than their linusorb-embedded counterparts, ranging from 15% – 100% with medians around 71%. The spacers are equally dominated by four categories of amino acids, with a small portion composed of 100% neutral hydrophilic amino acids. On average, the most abundant amino acids in the LLD domains on average are glycine (G, 16.4%), leucine (L, 13.1%) and tyrosine (Y, 9.50%) (**Supplementary Table 2**).

The distributions of amino acids in LLDs are more diverse than in linusorbs (**Figure 4c**). Linusorbs contain significantly higher proportions of neutral hydrophobic amino acids than LLDs (*p* < 0.05), even though LLDs are also dominated by neutral hydrophobic amino acids. Leucine (L) is one of the most frequently found amino acids shared by both linusorbs and LLDs. As a result, LLDs are richer in the other three amino acid categories than linusorbs. The spacers of both linusorbs and LLDs contain similar compositions of amino acids (**Figure 4d**). The most abundant amino acids in the spacers are the same as in the linusorb-embedded repeats: glutamine (Q, 25.7%), glutamic acid (E, 20.2%) and alanine (A, 9.0%) (**Supplementary Table 2**). Such coincidence is intriguing due to the fact that the spacers of LLDRs were left random in our previous proteome mining work. In summary, the amino acid composition mirrors the average hydropathy described above, and accounts for the moderate hydrophobicity of the linusorb molecules that are co-extracted with flaxseed oil.

### The conserved flanking signatures of linusorbs remain dominant in LLDs

**Figure 5:**
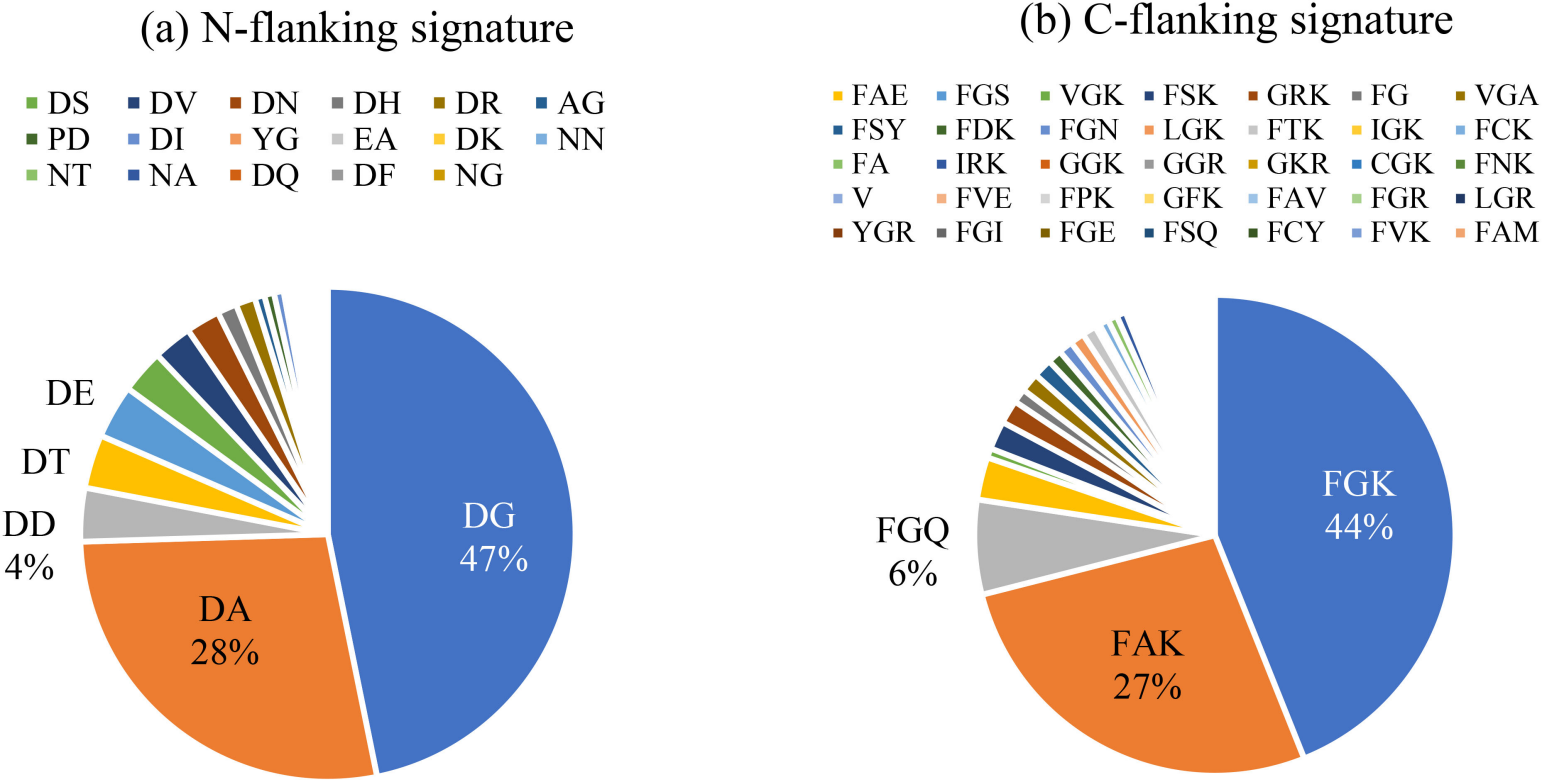
Pie charts illustrating compositions of **(a)** N-flanking signatures and **(b)** C-flanking signatures in the 315 LLDRs.

Our previous study reveals some conservations in the flanking signatures of LLDs (**Figure 1b** in (Song et al., 2025)). There are 22 unique N-flanking signatures (NFSs) in the 315 LLDRs (**Figure 5a**). Among these, DG (46.8%) and DA (27.7%) are the most dominant, followed by DD and other NFSs, each of which is lower than 4%. This is largely consistent with the 21 linusorb-embedded repeats, which are composed of 52.4% DA, 33.3% DD and 14.3% DG, but their frequency orders are different. Among the other 19 NFS variants coexisting with the three conserved NFSs, 11 start with D at the first position, with the second position being highly variable. This pattern is consistent with the NFSs in linusorb-embedded repeats, where the first position is absolutely conserved by D and the second position is occupied by G, D and A.

The 315 LLDRs have 38 unique C-flanking signatures (CFSs) (**Figure 5b**). However, the two dominant CFSs, FGK (44%) and FAK (27%), account for similar proportions as the 2 dominant NFSs. While FGK is conserved with the linusorb-embedded repeats, FAK is a major variant. Interestingly, the absolutely conserved G at the second position in the CFSs of linusorb-embedded repeats is present in only 58.1% of the 315 LLDR CFSs, whereas the less conserved F at the first position and K at the third position occur 92.3% and 81.8%, respectively. There are also more cases like the CFS of LO-C1 in G11-514P, with only two (FA, FG) or even one residue (V).

It is worth noting that the 25 LLDR-containing proteins were identified only by multiple occurrences (≥ 3) of repeat units matching the profile featured with the specified flanking signatures. In reality, many of the 25 proteins contain more than 3 repeats, some matching the profile while some having variations. The probabilities of having the conserved flanking signatures by chance (i.e. 1 in 20 possible amino acids) are 2.5e-3 for NFS (DG/DA/DD) and 1.25e-4 for CFS (FGK), respectively. The fact that the conserved flanking signatures remain dominant suggests that the conserved flanking signatures are not random, and the flanking sites are under strong selection pressure. These conserved flanking signatures might play an indispensable role in maintaining the processing and functionality of these proteins.

### Codon conservation accounts for both conservation and variation of flanking signatures

**Figure 6:**
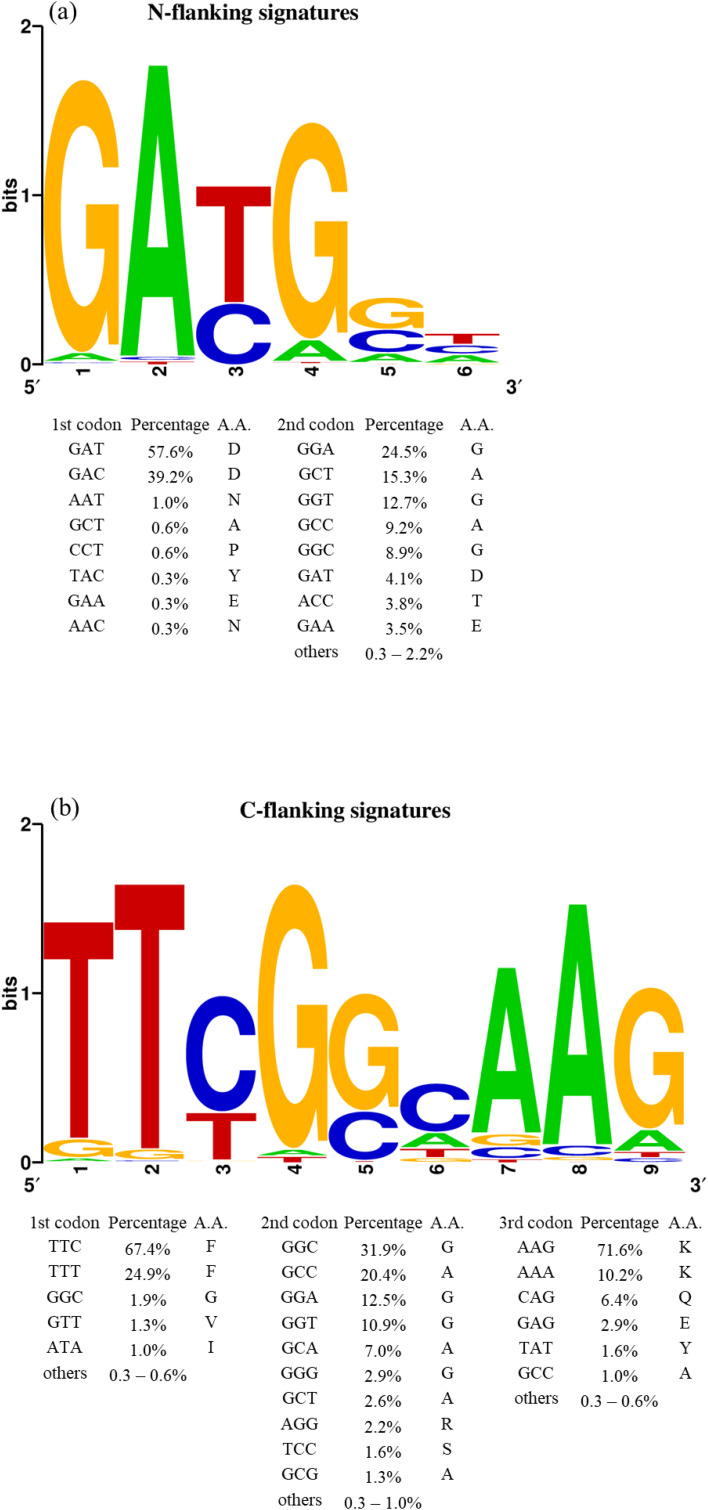
Coding sequence logos of **(a)** NFS and **(b)** CFS of LLDRs. Under the sequence logos are lists of codons with their percentages of the total codons in descending order and their encoding amino acids.

Although the flanking sites exhibit high conservation with minor variations, examining the nucleotide level variation reveals the mutations underlying the amino acid compositions of the flanking sites. In **Figure 6a**, the dominant D (96.8%) at the first NFS amino acid is encoded by two codons, GAT and GAC, differing only in the third codon position. The second amino acid is dominated by G and A, collectively accounting for 71% of all amino acid cases. The top 5 codons encode these two amino acids and differ only in the second codon position, where G is encoded by GGN and A is encoded by GCN (N is any of ACGT). As depicted in **Figure 6a**, the first codon position of the second NFS amino acid is dominated by G, and the second codon position is dominated by G and C, determining the second NFS amino acid to be either G or A. In **Figure 6b**, the first amino acid of the CFS is dominated by F (92.3%), which is encoded by two codons, TTC and TTT, differing only in the third codon position. The second amino acid is again dominated by G and A, accounting for 89.5% of all amino acid cases, encoded by the top seven codons where only the second codon position is the non-synonymous position. The third CFS amino acid is dominated by K, which is encoded by 71.6% AAG and 10.2% AAA as the top two dominant codons. The third most dominant codon, CAG, coding for the amino acid Q, is only one nonsynonymous point mutation from either of the first two codons. Therefore, the dominant amino acid and the major variants at the flanking signatures are encoded by conserved codons that differ in only one nonsynonymous position.

However, non-synonymous mutations could have a detrimental effect on the translated proteins. For example, the linusorb domain in G11-514P is flanked by FG- at the C terminus, where “-” is a stop codon. Among the 315 LLDR CSs, there are also five cases where the last repeat ends abruptly with a stop codon, with three FG- and two FA- in five proteins. These five stop codons contain 2 TAG, 2 TAA and 1 TGA. We discovered that the preceding LLDRs upstream of these five last LLDRs all have K as the third CS amino acid. As K is encoded by only two codons (AAA and AAG), four of the five stop codons (two TAG and two TAA) differ from the two codons for K in the first codon position, implying the possibility of non-sense mutation that substitutes the A at the first codon position for K with T, resulting in truncation of the protein during repeat duplication.

### Pairwise comparison of repeats indicates adaptive evolution

**Figure 7:**
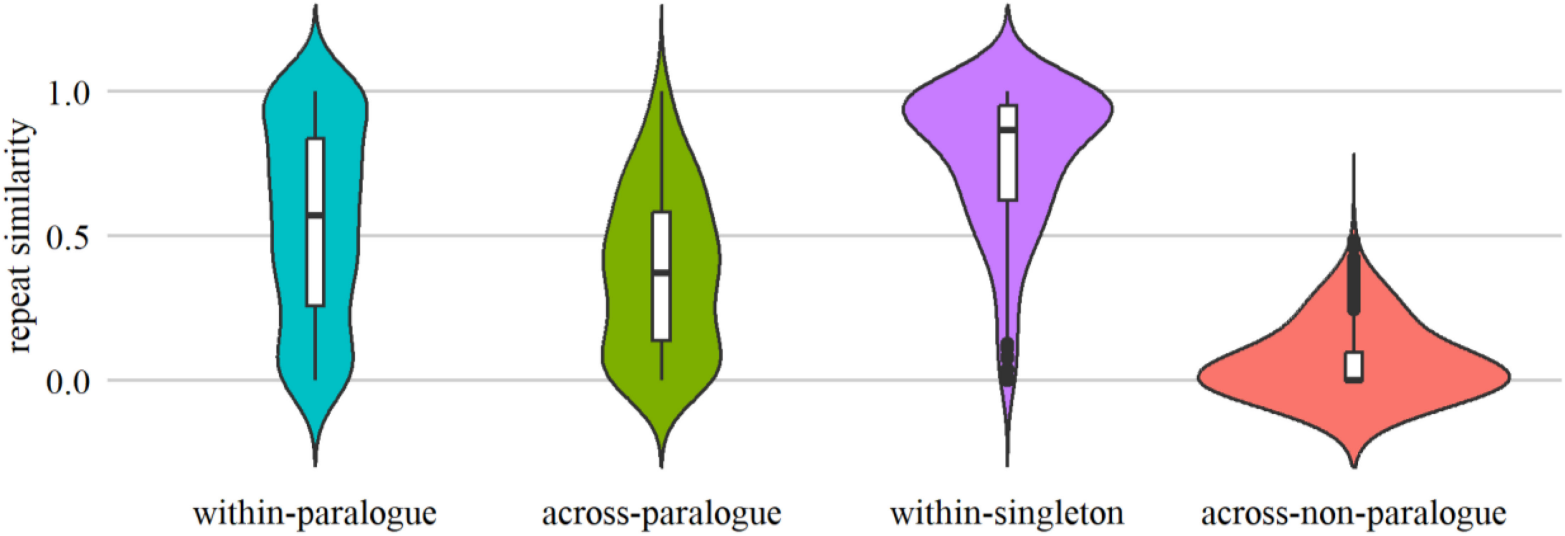
Violin plot showing the similarity distributions of RPs with different relationships. The number of RPs in each class is as follows: within-paralogue (2751), across-paralogue (4534), within-singleton (620) and across-non-paralogue (48360). The density areas are kept constant among all classes for better visualization.

As not all LRTRs are related, it is not reasonable to align all the LRTRs with a multiple sequence alignment. Otherwise, the alignment between two phylogenetically related LRTRs can be severely interfered with other unrelated LRTRs. In contrast, pairwise alignment can generate the most accurate comparison between two LRTRs. Thus, we conducted pairwise correlations of LRTRs within and across paralogous proteins (**Table 1**), within singletons (i.e. proteins with no paralogues) and across non-paralogous proteins. While all groups of RPs are significantly different from one another (*p* < 0.05), different relationships of RPs result in distinct patterns of similarity distribution (**Figure 7**). The boxes and whiskers inside the violins reveal that the first three RP classes exhibit similar ranges (0 to 1.0), whereas the last class has lower range (0 to 0.5). The interquartile range of RPs across paralogous proteins is slightly lower than within paralogues but significantly higher than across non-paralogous proteins, indicating that TRs diverged with some conservation in protein paralogues after ancestral gene duplication. Interestingly, RPs within singleton proteins have higher and narrower interquartile ranges than those within the paralogous proteins, suggesting that LRTRs within singleton proteins underwent low divergence and are under negative selection.

The density distribution varies from class to class. Within-paralogue RPs exhibit a nearly normal distribution, with most RPs in the range of 0.3 – 0.8 and the median above 0.5. Across-paralogue RPs have an up-skewed distribution, with most RPs ranging from ~0.1 to ~0.6. In contrast, the distribution of within-singleton RPs is down-skewed, with most RPs ranging from ~0.6 to 1.0. Most RPs across non-paralogues have nearly 0 similarity, and the remaining outliers have similarities up to 0.5. In summary, RPs within proteins have the highest similarity, followed by RPs across paralogous proteins, while RPs across non-paralogous proteins share little similarity. The relative difference in similarity distribution implies some adaptive divergence of TRs in protein paralogues.

Nevertheless, the signature of high within-paralogue similarity and intermediate across-paralogue similarity can also be resulted from concerted evolution via gene conversion and unequal crossover, which are well-documented mechanisms for tandem arrays. Thus, we conducted gene conversion analysis among paralogues to test this alternative scenario. The assay searched for highly similar and unusually long pairs of segments in the alignment of paralogues as candidates of possible gene conversion events. Statistical significance was assessed by determining pairwise and global *p*-values based on 10,000 permutations. Results are summarized in **Supplementary Data Sheet S5**. Among the 8 groups of paralogues, only one segment shared by *Lu14–5765* and *Lu6–41637* with a simulated *p*-value of 0.0421 is sufficiently similar to be suggestive of gene conversion. This fragment is identified to span four LRTR units with the translated sequence as follows: AKEQ/DGGLRQGYFAKQQ/DEGLRQGYFAKQQ/DG (units separated by/). These findings suggest that gene conversion rarely occurred while most of the paralogues evolved independently.

### Protein LRTRs shares consistent diversity of duplication patterns with DNA LRTRs

In a previous study, we identified three modes of repeat duplication at the DNA level (Song et al., 2025). Both single-unit and double-unit duplications were directly inferred from the similarity matrix pattern. The partial-unit duplication was deduced from in-depth analysis of the similarity matrix with additional evidence support from sequence alignment. In this work, the protein LRTRs were manually restructured to follow the order of 5 sections in the typical LLDR pattern consistently throughout all 30 proteins for better comparison. Therefore, it is uncertain if these LRTRs at the amino acid level exhibit duplication patterns similar to their coding sequences.

The similarity matrices of LRTRs in the 8 groups of paralogous proteins are provided in **Supplementary Figure 1**. In most cases, the patterns remain consistent with those of DNA TRs previously reported. There were five genes in which Tandem Repeat Finder (Benson, 1999) could not identify any repeats, and thus no similarity matrix was generated from these genes at nucleotide level, including *G11-516P* (Song et al., 2025). Here, all 30 proteins except G11-514P have LRTRs with LLDR pattern identified, and thus their LRTR similarity matrices can be constructed. The LRTR similarity matrices of 8 paralogue groups are displayed in **Supplementary Figure 1**.

In Group 1 consisting of G11-516P and G14-170N (**Supplementary Figure 1A**), the matrix patterns of both paralogues indicate single-unit tandem duplication. For other groups of paralogues where both DNA LRTRs and protein LRTRs are available, most exhibit consistent patterns indicative of the same duplication modes as previously reported at nucleotide level. Two distinct exceptions lie in G4-136N and Lu11-28070, where LRTRs of both proteins exhibit a mosaic pattern (**Supplementary Figures 1B, G**), implying double-unit duplication, while such pattern was not evident in the similarity matrices at nucleotide level [**Figure 3a** and e in (Song et al., 2025)].

For G4-136N, there are 5 LRTRs identified in the gene, but 9 LRTRs in the protein. The nearly double number of LRTRs in the protein suggests each LRTR in the gene encodes two LRTRs in the protein. However, the two adjacent LRTRs in the protein have a similarity lower than 50%, resulting in a mosaic pattern. For Lu11-28070, both gene and protein have 21 repeats identified. However, the ones in the gene are not tandem but distributed in five separate regions, each forming a red triangular block indicative of similarity higher than 50%. Only LRTRs 10–16 in the middle of the protein exhibit mosaic pattern while the flanking ones are in two red triangular blocks. These discrepancies in repeat structures identified between protein and gene could be ascribed to codon degeneracy in gene sequences. The gene sequences can be more variable but code for conserved protein sequences.

There appears a significant drop in the RP similarity across paralogues compared to within each paralogue, as indicated by the blue rectangular blocks in the matrices. However, in Groups 2 (**Supplementary Figure 1B**) and 3 (**Supplementary Figure 1C**), such blue rectangular block is not obvious, suggesting that the LRTR similarities across paralogues are comparably high to those within paralogues. These minor cases support the above violin plot in **Figure 7** for the across-paralogue class, which shows that most RPs share 50% similarity or lower. This implies the possibility of positive selection on paralogues after gene duplication, which is further investigated in the next section.

### Nonsynonymous substitutions in both LLD and CS regions indicate positive selection

**Figure 8:**
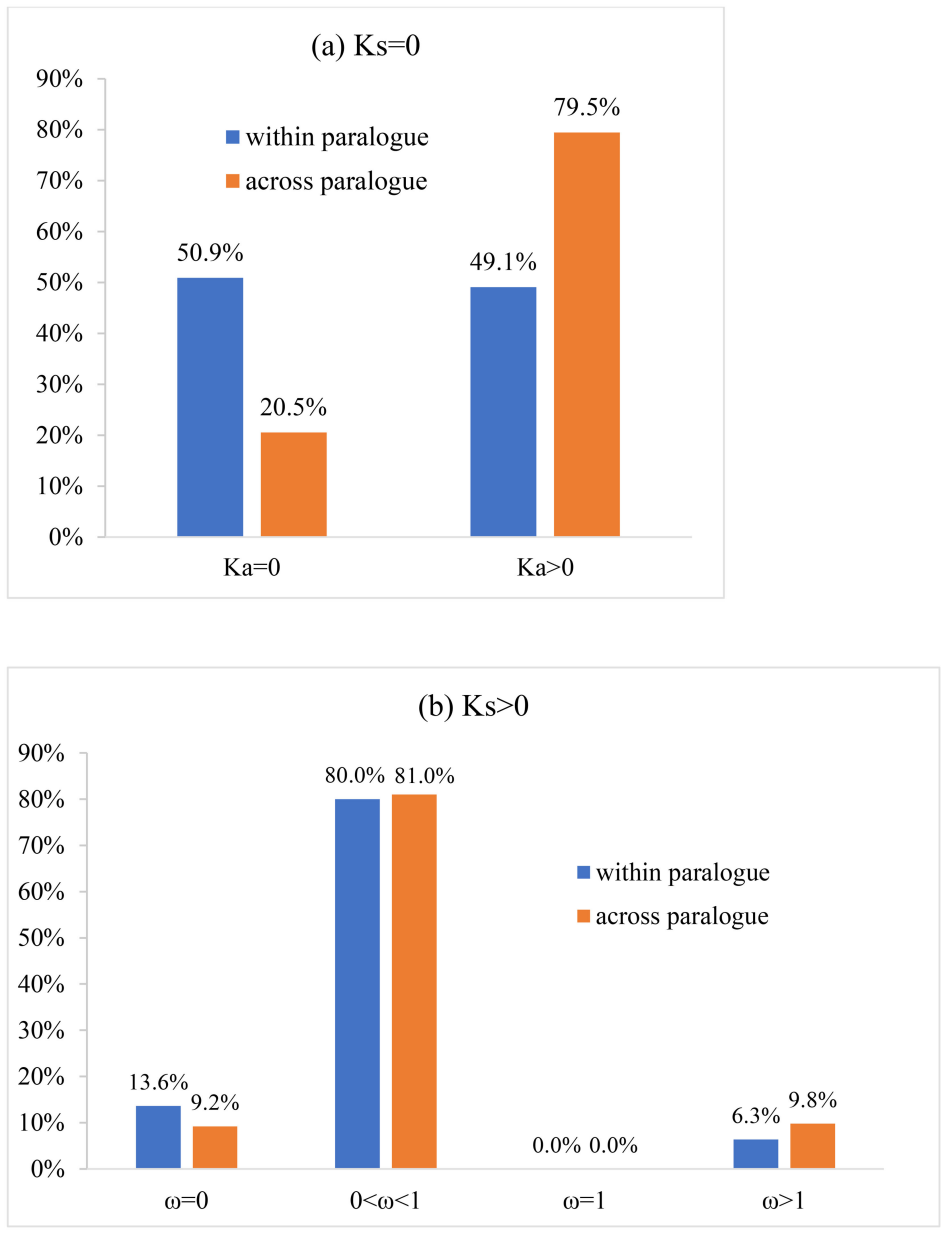
Comparison of repeat pairs (RPs) within and across protein paralogues in terms of **(a)** different Ka values when Ks = 0 and **(b)** Ka/Ks ratios (ω) when Ks >0.

Difference in the similarity distribution between RPs within and across paralogues implies different selection on LRTRs. To test this hypothesis, we estimated Ka/Ks ratios (ω) of RPs using the maximum likelihood (ML) method. We first built an ML tree using IQ-TREE and found that many branches of the ML tree lack adequate statistical power due to the short lengths and high similarities of LRTRs. However, a reliable Ultrafast bootstrap value should be 95% or higher (Trifinopoulos et al., 2016), so we abandoned the ML tree and estimated the ω of RPs instead. As many of the RPs are highly similar, 222 (9.3%) of the total 2380 RPs within paralogues and 1212 (28.1%) of the total 4312 RPs across paralogues have no synonymous substitutions (Ks =0) and thus Ka/Ks ratio are not available for these RPs. Among the RPs with Ks =0 (**Figure 8a**), 50.9% within paralogues and 20.5% across paralogues have no nonsynonymous substitutions either (Ka =0), indicating identical RPs. The remaining RPs with nonsynonymous substitutions but no synonymous substitutions (Ka >0 and Ks =0) across paralogues (79.5%) are 30% more than those within paralogues (49.1%), providing strong evidence of positive selection. Among the RPs with Ks >0 (**Figure 8b**), the proportions of 0<ω<1 and ω=1 are comparable between the within-paralogue RP series and the across-paralogue RP series. Notably, the within-paralogue RP series has 4.4% more ω=0 RPs and 3.5% fewer ω>1 RPs than the across-paralogue RP series, suggesting that nonsynonymous substitutions are dominant in the across-paralogue RPs.

The pairwise estimation of ω assumes all sites are under identical selective pressure, and the ω is averaged over all sites. However, the selective pressure may vary from site to site, so we then identified sites that are under positive selection in LRTRs across paralogues. Site models allow variations in selective constraints among sites, and results of the five site models for the eight paralogue groups are presented in **Table 2**. For groups with a significant likelihood-ratio test (LRT) for either M1a – M2a comparison or M7 – M8 comparison, the Bayes Empirical Bayes (BEB) procedure was carried out to identify positively selected sites. Only Group 1 had a significant LRT, and its 35E in the CS region was identified as positively selected site among LRTRs within and across the paralogues, with a posterior probability of 1.

**Table 2:** Likelihood ratio test (LRT) statistics for LRTRs of 8 paralogue groups under different site models.

Group	*lnL*		2Δ*l*	*P*-value	*lnL*		2Δ*l*	df	*P*-value	BEB positively selected sites	Pr (ω>1)
	M1a	M2a			M7	M8					
1	-403.15	-396.30	13.69	0.0011	-402.82	-396.11	13.42	2	0.0012	35 E	1.00
2	-308.47	-308.47	0.00	1.0000	-308.44	-308.43	0.01	2	0.9985	none	N/A
3	-271.07	-271.07	0.00	1.0000	-271.10	-271.10	0.00	2	1.0000	none	N/A
4	-478.59	-478.59	0.00	1.0000	-478.43	-478.36	0.16	2	0.9233	none	N/A
5	-391.23	-391.23	0.00	1.0000	-391.00	-391.00	0.00	2	1.0000	none	N/A
6	-917.16	-917.16	0.00	1.0000	-912.19	-911.69	1.00	2	0.6074	none	N/A
7	-1087.88	-1087.88	0.00	1.0000	-1084.74	-1084.74	0.00	2	1.0000	none	N/A
8	-725.20	-725.20	0.00	1.0000	-723.46	-723.46	0.00	2	1.0000	none	N/A

*lnL*, log-likelihood score; 2Δ*l*, twice the difference of log-likelihood scores between the models; df, degree of freedom; Pr, posterior probability.

The above analyses using site models are conservative as they only detect sites under positive selection throughout the entire phylogeny of LRTR evolution, from ancestral gene duplication to LRTR duplication within paralogues. It is hypothesized that positive selection may act on certain sites of certain branches after gene duplication. Therefore, the branch-site model, which allows ω to vary among sites and branches, was applied to the LRTRs of paralogous genes. We tested each significant node with an Ultrafast bootstrap value ≥95% for the branch-site model. For every analysis, one significant node was labeled as “foreground” with “$1” and the rest of the tree as “background”. The LRT for branch-site model A against the null model showed significant support for the foreground clade in the phylogenies of LRTRs across paralogous genes.

**Table 3** lists positively selected sites with posterior probabilities ≥ 0.950 identified by the BEB method. Some foreground clades contain LRTRs of purely one gene, including all LRTRs in G11-516P of Group 1, LRTRs 2 and 11 in Lu8–3470 of Group 4, LRTRs 3–9 in Lu11–24918 of Group 7 and LRTRs 2 and 4 in Lu10–38024 of Group 8. This suggests that positive selection acted on these LRTRs of only one paralogue in each group, while LRTRs in the other paralogues are mostly under purifying selection. In other cases, positively selected sites were detected in LRTRs of two or more genes. Notably, in some paralogues, positively selected sites were detected in multiple, consecutive, or all LRTRs, indicating positive selection occurred either episodically or consecutively.

**Table 3:** LRTRs with sites under positive selection detected by branch-site models and BEB method with posterior probabilities ≥ 0.95.

Group	Foreground clade taxa	Position (aa)	Residue	Region
1	all LRTRs of G11-516P	16	F	LLD
		18	W	LLD
		26	Q	CS
		36	S	CS
		37	S	CS
2	G3-449N_2,3 and G4-136N_2,4,6	4	I	LLD
		7	F	LLD
		10	T	LLD
		14	K	CFS
		17	A	CS
		19	V	CS
3	none	none	none	none
4	Lu8-3470_2,11	19	K	CFS
5	Lu5-46938_4 and Lu8-2811_3	24	–	CS
		26	E	CS
		27	M	CS
6	Lu6-41637_37 and Lu12-11698_33	8	Y	LLD
	Lu12-11761_2,3,4,18,33 and Lu12-11698_5,7,8 and Lu6-41637_37	8	Y	LLD
7	Lu10-34966_17 and Lu11-24918_10	9	G	LLD
		11	P	LLD
		13	L	LLD
		14	S	LLD
	Lu11-24918_3,4,5,6,7,8,9	10	Y	LLD
		11	P	LLD
		12	P	LLD
		14	S	LLD
8	Lu10-38024_2,4	24	–	CS
		25	–	CS

In total, 27 sites are identified under positive selection, of which 15 are in LLD (56%), 2 in the CFS (7%) and 10 in the CS region (37%). This is consistent with the fact that both the LLD and the CS regions are highly variable at the amino acid level (i.e. nonsynonymous substitutions are frequent), while the CS region is relatively conservative with some variations, especially at the third position. Position 35 was identified under positive selection throughout LRTR evolution in both paralogues by the site models, but not the branch-site models. This difference can be explained by the different assumptions and goals of the two models. The site models assume all branches share one single ω and identify positively selected sites with ω>1. The branch-site models allow ω to vary among branches and sites, and compare whether the alternative hypothesis, which assumes the specified foreground branch/clade contains positively selected sites with ω>1, provides a significantly better fit than the null hypothesis, which assumes both the foreground and background are under neutral or purifying selection with 0<ω≤1. The five positions identified in **Table 3** (16, 18, 26, 36 and 37) are under positive selection only in the LRTRs of G11-516P (foreground), whereas Position 35 is under positive selection across all LRTRs of both paralogues.

**Figure 9:**
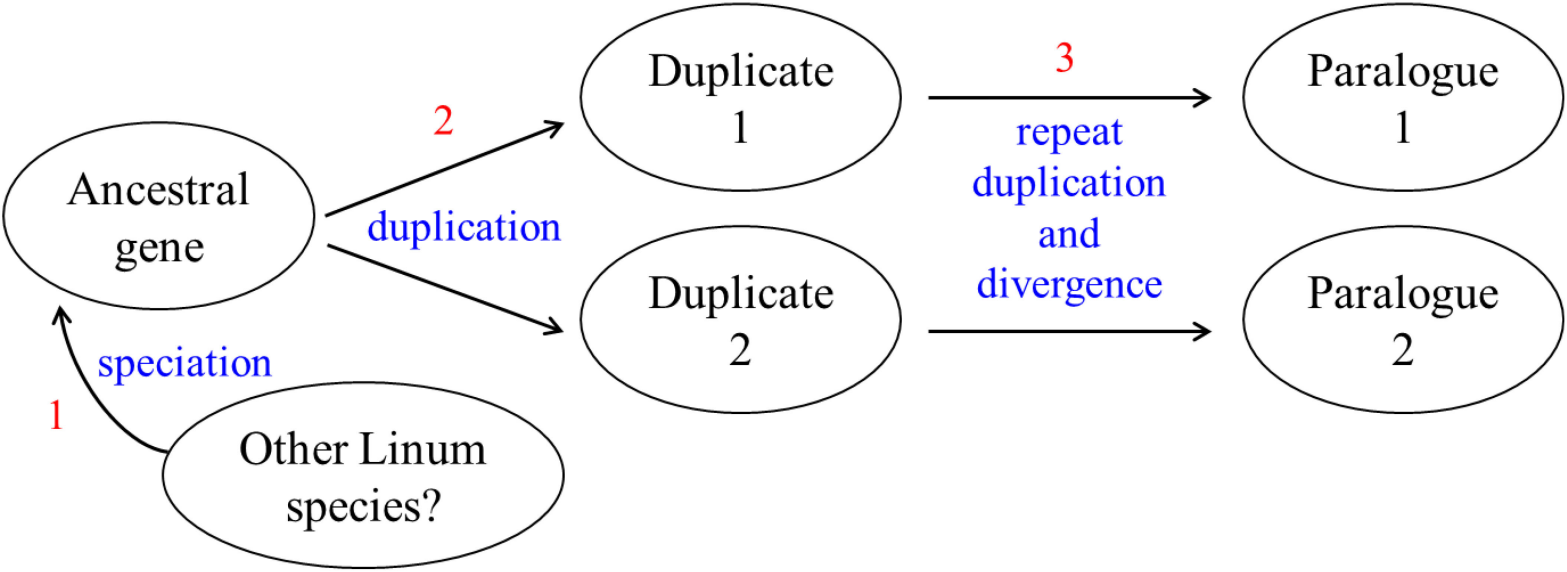
Schematic representation of a multi-dimensional model of adaptive evolution.

Overall, these positively selected sites located at various regions of the paralogous proteins, across regions within a protein and across paralogues constitute a multi-dimensional model of adaptive evolution (**Figure 9**). In this model, Dimension 1 involves LRTR evolution among orthologues in the Linum genera or even higher hierarchies. Such speciation-driven evolution is yet to be investigated. Dimensions 2 and 3 involve LRTR evolution among paralogues within a species’ genome and has been elucidated in the present study.

### None of the LRTR regions are annotated

Only one protein, Lu1-17016, is annotated with the flax amaranthin-like lectin 4 (LuALL4, GenBank accession: AIU47275.1), which functions in defense response (GO: 0006952). Note that Lu1–17016 is a singleton without any paralogues among the 30 proteins. Our preliminary search of the ClusteredNR database by BLAST revealed that none of the 30 proteins have reliable hits (E-value < 1.0E-5 with manual examination of the local alignments), indicating no homology to any well-characterized proteins, except LuALL4, which had an identical match (E-value = 0.0) and was unambiguously identified. LuALL4 contains two agglutinin domains (Pfam PF07468) as the characteristic domain of the amaranthin-type lectin family and a pathogenesis-related (PR) protein Bet_v_I domain (PF00407) (Faruque et al., 2015). The two agglutinin domains, one located at 3–155 aa and 187–340 aa, respectively, and the LLDR region located at 413–767 aa, 73 amino acid N-terminal to the agglutinin domains. The Bet_v_I domain located at 872–1017 aa is 105 aa C-terminal to the LLDR region. Although we searched InterProScan and retrieved the same identification of the three domains, we did not find any characterized domain that matches the LLDR region, which was roughly predicted as an intrinsically disordered region by the MobiDB-lite method in InterProScan. As there are 19 LuALL proteins identified, we used RADAR to search for any TRs in the other 18 LuALL proteins, but none of them, other than LuALL4, matches the LLDR pattern. It is also noteworthy that LuALL4 has no signal peptide identified.

The other 29 proteins were analyzed by InterProScan for the presence of any known domains. In general, these proteins are poorly characterized by limited information on different regions of the protein (Data S2). Nonetheless, the following structural regions were detected in most proteins with posterior probabilities higher than 6:

- A signal peptide extending from the N-terminus to the beginning of the repeat region, identified by Phobius or SignalP. The presence of signal peptide in most of the LLDR-containing proteins is consistent with the known linusorb precursor proteins and is a common feature shared by other cyclic peptide precursor proteins (Arnison et al., 2013). The signal peptide is an indication of secretion, in which the signal peptide is cleaved from the precursor protein and the rest of the protein is transported to different target locations.
- The region spanning from LRTRs to the C-terminus was identified as non-cytoplasmic by Phobius, suggesting that the protein is directed towards the secretory pathway after synthesized by ribosomes, and the LRTRs may undergo post-translational modification at other organelles.
- Another structure is intrinsic disorder predicted by the method MobiDBLite. Detected in 7 proteins, the intrinsically disordered regions distributed in almost any location of the protein.

### Most of the LLDR-containing proteins are targeted to the plastid

Analysis with InterProScan retrieved limited information on different regions of the 30 proteins, only characterizing some general structures such as a signal peptide. To complement the InterProScan results we used TargetP 2.0 (Almagro Armenteros et al., 2019) to predict the N-terminal sorting signals of the 30 proteins. All the 5 linusorb precursor proteins and 15 LLDR-containing proteins harbor a chloroplast transit peptide (cTP) with significantly higher probabilities than other types of N-terminal pre-sequences (**Supplementary Table 3**). The predicted cleavage sites are located between the signal peptide and the LRTR region, implying that these proteins are subject to post-translational modification, which removes the cTP during or after the entry to the chloroplast for further processing of the remaining sequence (Emanuelsson et al., 2000). Two proteins, Lu5–45630 and Lu5-47766, contain a signal peptide (SP), suggesting their destination to the endoplasmic reticulum (ER) for subsequent transport through the secretory pathway. The remaining eight proteins are categorized as “other”, meaning no targeting signal is predicted. We then checked whether they are integral membrane proteins using TMHMM 2.0 (Krogh et al., 2001), but found no transmembrane domains. We speculate that the possible destinations of these eight proteins can be cytoplasm, nucleus or peroxisome.

## Discussion

### LRTR evolution in paralogous proteins: a multi-dimensional model

Pairwise comparison of LRTRs and categorization of RP similarities by the relationship of their proteins (within paralogues, across paralogues and across non-paralogues) shows how RPs under different categories differ in the distributions of similarity. Such different distributions, in combination with the rarity of gene conversion events, indicate adaptive evolution of LRTRs among paralogous proteins. According to the classical model of adaptive evolution in the context of gene duplication (Ohta, 1991; Swanson, 2003), paralogues derived from ancestral gene duplication can accumulate mutations and develop diverged functions under positive selection, followed by purifying selection that maintains the new functions in one or more paralogues (Bielawski and Yang, 2003). The moderate similarities of RPs across paralogues at the level between those within paralogues and those across non-paralogues match the pattern of adaptive evolution in general.

However, the presence of repeats complicated this prototype. Repeat divergence after gene duplication is accompanied by repeat duplication in the paralogues. The similarity distribution of RPs in **Figure 7** indicates that there are more highly similar RPs within paralogues than across paralogues. The high similarity of within-paralogue RPs might be due to conservation under purifying selection or recent repeat duplication without enough time for mutation to accumulate. The low similarity of across-paralogue RPs is indicative of divergence, but it is unclear whether such divergence results from positive selection on the original repeats from ancestral gene duplication or on newly generated repeats in the paralogous proteins. Although it might be challenging to distinguish the source of selection, it is important to recognize that different sources of selection have distinct evolutionary implications. We attempted to unravel this multi-dimensional evolution model from the similarity matrices among paralogues but there seems to be no pattern on the distributions of significantly similar RPs and their insignificant counterparts across paralogues. Nevertheless, selection analysis indicated that positive selection has episodically acted on certain lineages and sites of LRTRs. Both pieces of evidence shed light on the multi-dimensional model of adaptive evolution with respect to LRTRs in paralogous proteins. The positively selected sites and lineages of LRTRs may have implications of functional divergence that needs experimental verification.

### LRTR evolution diversifies the product of post-translational modification

Protein TRs have widely been recognized for their influence on protein stability, flexibility and specificity of ligand binding (Andrade et al., 2001). These TRs come in various conformations such as an open rod-like or superhelical structure, which exposes an extensive surface accessible for solvents and suitable for binding larger substrates like proteins and nucleic acids (e.g. HEAT domain) (Groves et al., 1999). On the other hand, a closed barrel-like structure provides a compact and stable surface that facilitates interactions with smaller ligands (e.g. Kelch domain) (Adams et al., 2000). Despite exhibiting low sequence conservation, a few conserved residues within TRs are crucial for their proper folding (Björklund et al., 2006).

While the protein precursors of many CPs, such as linusorbs, also display sequence variation with conserved sites, their primary outcomes differ. In linusorb precursor proteins, LRTRs maintain consistent lengths and structures, as demonstrated in our previous study through sequence alignments (Song et al., 2022). The highly conserved sites flanking linusorb domains ensure specific recognition by proteolytic enzymes, while the linusorb domains are allowed to diverge through mutations, resulting in peptide products with sequence polymorphism and a uniformly circular structure. In contrast, in the case of resistance genes (*R* genes), the most polymorphic bases are found in the characteristic LRR-coding region. This extensive variation encodes essential information for gene-for-gene specificity, enabling the *R* protein to interact with the products of pathogen *avirulence* (*avr*) genes (Ellis et al., 2000). Since CPs also serve as defense agents in plants, it is likely that the variability of the peptide domain plays a crucial role in the specificity of receptor-ligand binding.

Unlike the conserved sites in protein TRs that contribute to maintaining the structural stability of the protein, conserved sites flanking the peptide domain recruit proteolytic enzymes to modify the precursor protein. This process is similar to other proteases that recognize specific amino acids and cleave the proprotein into peptides. However, the protease for CP synthesis is a specialized protease capable of performing cyclization instead of simple digestion (Condie et al., 2011; Mylne et al., 2011). Interestingly, the cyclization of peptide precursor represents an opposite analogy to protein splicing. In protein splicing, an internal protein segment known as intein is excised from a precursor protein, and the remaining portions called exteins join together via a peptide bond (Gogarten et al., 2002). In contrast, a CP is formed by ligating both termini of an “intein”. While protein splicing can occur spontaneously among specific amino acids such as cysteine and serine via nucleophilic attack (Wood et al., 2023), peptide cyclization requires the catalytic action of proteolytic enzymes. Although peptide cyclization is more complex than protein digestion and splicing, as it requires more specific proteases, the circular structure of CPs makes them unique and confers an evolutionary implication, e.g. resistance to biotic interactions in seeds. This post-translational modification presumably plays an indispensable role in increasing the fitness of a species.

Gene duplication serves as another contributing factor to divergence, allowing paralogues to evolve under relaxed selective constraints (Schaper and Anisimova, 2015). The genome of *Arabidopsis thaliana* contains over 150 NBS-LRR genes that have diversified through various mechanisms (Leister, 2004). Similarly, in the genome of *L. usitatissimum*, 21 out of the 30 linusorb-related genes form eight groups of paralogues, with each group consisting of two to four paralogues. LRTRs across paralogues show lower similarity compared to LRTRs within a paralogue, indicating divergence following gene duplication. Additionally, polymorphisms in the leader and follower sequences among paralogues suggest functional divergence, potentially leading to different metabolic pathways and regulation of the precursor proteins.

While paralogues arise from gene duplication, the mechanisms by which non-paralogous genes may interact with one another remain unclear. A more comprehensive investigation is necessary to unveil the diversification of this extensive repertoire of LRTR-containing genes. Furthermore, the paralogues in a cultivated flax genome only represent a portion of the evolutionary story, and it would be useful to explore potential orthologues in other *Linum* species. This approach would help to reconstruct the evolution of linusorbs during speciation and enhance our understanding of the role these protein LRTRs play in adaptation.

## Data Availability

The data supporting the findings of this study are available within the article and/or its **Supplementary Materials**.

## Supplementary material

Supplementary Table 1 Repeat sections of 30 proteins.

Supplementary Table 2 Amino acid compositions of linusorbs, LLDs and corresponding spacers.

Supplementary Table 3 Subcellular localizations of 30 proteins.

Supplementary Figure 1 Similarity matrices of 8 paralogous groups.

Supplementary Data Sheet 1 Sequence alignments of paralogous proteins.

Supplementary Data Sheet 2 Domains identified in 6 proteins by Interproscan.

Supplementary Data Sheet 3 Amino acid compositions.

Supplementary Data Sheet 4 300-random-sequences.

Supplementary Data Sheet 5 Gene conversion test results.
