# Supplementary material for: In silico analysis reveals a multi-dimensional model of adaptive evolution in the flax orbitide-related precursor protein family
Source: Front Plant Sci. 2026 Jun 30;17:1824173. doi: 10.3389/fpls.2026.1824173 (PMC13365257; doi:10.3389/fpls.2026.1824173)
Supplement: Supplementary Table 1 — Repeat sections of 30 proteins. [file Supplementaryfile1.zip › Data S1_Paralogue alignments.docx]

Group 1

G14-170N MAAASSLALATASLVATGAGGRNNAFLPS---KNKTP-NLFLNPNKTTSSTVKAVVSSSS

G11-516P MAVVSSLAL-TTSLVATAAGRNNNAFPPSSSRNNKAPADLFITPKTTTTVKAAAV----S

G11-514P -MAASSVPL-TTSLVATAAAGRNN--------NSKTPANLFLTPKTSTVKAAV------S

..**:.* *:*****.*. .** :.*:* :**:.*:.:* . *

G14-170N CKRPYPKGDASLFLGIDDVFGKDAVAGHDNDQDAASGQEMAADDMLMPFFWIFGKEGQQQ

G11-516P CKRPYPKG---------AVAAATSTLSPISGKDGGLRNQEESDGMLVFPLFIFGKEGSQD

G11-514P CKLS--------------------------------------------------------

** .

G14-170N EAEESSDDMLMPFFWIFGKEGQQQEAESSDDMLLPFFWIFGKEGQQ--------------

G11-516P KYN--------------GAAALRDQEESDGMLIPPFFVIFGKEGCQDIGHKYNNAAAAGA

G11-514P -----------------GSHHHHHQEEGSG------------------------------

* . : *...

G14-170N -QEAESSDDMLMP-FFWIFGKQQQQQGESSDDMLMPFFWVFGKQGDNNKGDAVEAILKN

G11-516P LRDQEESDGILVPPFFLIFGKEGSQDKYNAAA-------AGGLRGKEQQGDKMAAGAEN

G11-514P -----GGDDMLKPFFFWIFG---------------------------------------

.*.:* * ** ***

Group 2

G3-449N MAIASSTFTLALPSLGSSPSPFKGRAHIGLAPVLKARKTSATTLSRETLISHSSKLHHSL

G4-136N --MASSAFTLALPSLGSSPSPFNGRAHVGLPPVLKARKTPIVSSSK---LHSTLKKHEVV

:***:***************:****:**.********. .: *. : : * * :

G3-449N LKKSGDAGI-------------------------------------GDDGIPPFWLTLFG

G4-136N DSERGDAGIPPFWLTLVGKQRTDVLNSKLGDAGLPPMWVEVFGSERGDAGIPPFWLTLVG

.: ***** ** *********.*

G3-449N KQQANVFNSEKGDAGMAPMWVTVFGSERG-------------------VFNSEKGDAGMA

G4-136N KQRTDVFNSKLGDAGLPPMWVEVFGSERGTDAGIPPFWLTLVGKQHANVFNLEKGDAGLP

**.::****: ****:.**** ******* *** ******:.

G3-449N PVWVTVFGSERG-------------------VFNSEKGDAGMAPVWGD------------

G4-136N PMWVEVFGSERGTDAGIPPFWLTLVGKQHANVFNSEKGDAGLPPMWVEVFGSERGTDAGI

*:** ******* **********:.*:* :

G3-449N -GFWLR------ERCF-------

G4-136N PPFWLTLIGKHAGQIVDSTSVNT

*** . .

Group 3

Lu2-51734 MMASSLAVSAASLVVTAAGTNVFPSRNTPNFFLANNKSTSPLKPIISCHPGGGSLRSKAH

Lu13-23576 MFGK--------------------AHQDGSVFVGQSSLFGKAHQDGSVFVGQSSLFGKAH

*:.. :.: ..*:.:.. . : * . * .** .***

Lu2-51734 QDGSLFGGQGAMFGKAHQDGSLFGGQGAMFGKAHQDGSLFGGQGAMFGKAHQDGSLFGGQ

Lu13-23576 QDGSLFGGQGAMFGKAHQDGSVFVGQSSLFGKAHQDGSLFGGQGAMFGKAHQDGSVFVGQ

*********************:* **.::**************************:* **

Lu2-51734 GAMFGKAHQDVHQDGSLFGGQGAMFDKAHQDGSLFGGQGAMFGKAHQD---------VHQ

Lu13-23576 GSLFGKA----HQDGSLFGGQGAMFGKAHQDGSVFVGQSSLLGKAHQDGSLFGGQGAMFG

*::**** **************.*******:* **.:::****** :.

Lu2-51734 DVHQDGSLFGGQGAMCGKAHQDGSLFGGQGAMFGKAHQDGSLFGGQGAMFGKAHQDGSLF

Lu13-23576 KAHQDGSVFVGQSSLFGKAHQDGSLFGDQGAMFCKAHQDGSVFVGQSSLFGKTHQDGSLF

..*****:* **.:: ***********.***** *******:* **.::***:*******

Lu2-51734 GGQGAMFGKAHQDGALFGGQGAMFGKVHQDGSLFGGQGAMFGKAHQ--------DGSLFG

Lu13-23576 GGQGAMFGKAHQDGSVFVGQSSLFGKAHQDGSLFGGQDARFGKAHQDVHQDGHHDGSLFG

**************::* **.::***.**********.* ****** ******

Lu2-51734 GQGAMFGK----AHQDGSLFG--------GQGAMFGKGQDVKGGDNFVAAPILKE

Lu13-23576 GQGAMFGKAHQDAHQDGTLFGKARVEGDLGDGSLFGKGQDVKRGDNFVAAPILKE

******** *****:*** *:*::******** ************

Group 4

Lu5-45630 MAPSHLRFLLLALVLLGAGCCSVNGIRKDPIGDYWRSVMKDEPMPKAIQSLLLPAANSDH

Lu8-3470 --------MILMLLQLGADSVNA---RKDSIGGYWKSVMKDQLMPKAIES-LLPAANSDH

::* *: ***.. .. ***.**.**.*****: *****:* *********

Lu5-45630 ------QHPAVHYDTD----HNFKNPDQSQPDEHIFEYYSKDDALPSNKFAKGIGLKPDE

Lu8-3470 ADVRCDTNQVFQYHKDAVPSNEFSKEIESQPNQ-VFQYH--KDIVPSNEFAEGTESQPNQ

: ..:* .* ::*.: :***:: :*:*: .* :***:**:* :*::

Lu5-45630 ALLFYSKDDAPPSNKFAKGIGLKPDEALLFYSKDDAPPSNKFAKGIGLKPDEALLFYSKD

Lu8-3470 VFQYH--EDAVPSNEFAKEIELQPNQVFQYH--KDAVPSNEFAKEIESQPNQVFQYH--K

.: :: :** ***:*** * *:*::.: :: .** ***:*** * :*::.: :: .

Lu5-45630 DAPPSNKFAKGIGSKPDEALFFYSKD-----------------------DAPPSNKFAKG

Lu8-3470 DAVPSNEFAKEIESQPNQ-VFQYHKDVVTSNEFSKGMELQPNQVFQYHKDIVPSNEFAKG

** ***:*** * *:*:: :* * ** * ***:****

Lu5-45630 IGSKPDEALFFYSKDDAP-----------------------PSNKFAKGIGSKPDEALFF

Lu8-3470 IESQPNQ-VFQYHKDVVPSNEFPKEIETRPNQVFQYHKDVVPSNEFSKGIGSQPNQ-VFQ

* *:*:: :* * ** .* ***:*:*****:*:: :*

Lu5-45630 YSKD-----------------------DAPPSNKFAKGIGSRPDEALLFYSK--------

Lu8-3470 YNKDVVPSNEGFKEIESQPNQVFQYHKDAVPSNEFAVGTETQPNQVFQYHKDVVPSNEFA

*.** ** ***:** * :.*::.: ::..

Lu5-45630 -----

Lu8-3470 KGIES

Group 5

Lu5-46938 MSMKSGRKGDSAALLTIASKARDAGLIPVPMFGLAETSNAPEGDAGIIDPILLIFGKLEM

Lu8-2811 MSMKSGRKGDSAALLTFASKG-DATLIPVPMFGKAEMMD-RKGDAGIIDPTLPIFGKVEM

****************:***. ** ******** ** : :******** * ****:**

Lu5-46938 MARNGDAGFVPNFSVFGKEDAALFIPSFPIFGKEERMAPKGDAGFDIFFPFFGKADIMMP

Lu8-2811 MARN----------------------------KVEMMAPKGDAGFDIFFPFFGKQAERMP

**** * * ****************** **

Lu5-46938 PKEDAGFDIFFPFFGKQAEMMTPNG--------------------------DDGPSA

Lu8-2811 PKEDAGFDNFFPFFGKQAEMMTPKGVTLRVHAQDHDINVLSFHRHICLINYKNLMSS

******** **************:* .: *:

Group 6

Lu14-5765 ------MEVSYEITS----PSSRTK---VFYETTSPRSRTEVSCETTLISS--------R

Lu6-41637 ------MPAAATFSSVASSPPQHSKKNMTTMLTASLKTSSKVSVSAAVVSFNKQQDGGIR

Lu12-11761 MASSFAMPAAATFLPVASSPPQHSKKNMTMTLLTSPKTSSKVSVSAVVVSVNKQQDGGIR

Lu12-11698 MASSFAMPAAATFSSVASSPPQHTKKNMTTVLTASPKTRSKVSVSAAVVSFNKQQDGGIP

* .: : . *...:* . :* .: ::** .:.::*

Lu14-5765 TEVFCETT----------------------------------------------------

Lu6-41637 RTYFAKQQDGGLRQGYFAKEKDGGLRQGYFAE--DGGLLRDYFAKEQDGGLRQGYFAKQQ

Lu12-11761 LPYFAKQK---------------------------------------DGGVWDAFFGK--

Lu12-11698 RTYFAKQTDGGLLKDYFAKKHDGGIRRTYFAKQYDGGLLKDYFAKEYDGGEWAAFFGKQH

*.:

Lu14-5765 -----------LPNSG--TVVLCAPPSPRNRTHVFYETTSPRSKTEVSCE------TTSI

Lu6-41637 DEGLRQGYFAKQQDGGLRQGYFAKEKDGGLRQGYFAKEKDGGLRQGYFAE--DGGLLRDY

Lu12-11761 -----------QQDGGEWAAFFGKQEDGGEWAAFFGKQQDGGIRRTYFAKQRDEGLLGDY

Lu12-11698 DGGIRRTYFTKEHDGGEWAAFFGKQHDGGEWAAFFGKQQDEGIRRTYFAKQHDRGLLRDY

:.* : . . * : . . .: .

Lu14-5765 SSRTEDGGLLRDYFAKEQDGGLLRDYFAKEQ---------DEGLLRDYFTKEHDRGLSRD

Lu6-41637 FAKEQDGGLRQGYFAKEKDGGLRQDYFAE-----------DGGLLRDYFAKEQDGGLRQG

Lu12-11761 FAKQEDGGLLGDYFAKQEDGGLLGDYFAKQR---------DGGLLGDYFAKQEDGGLLGD

Lu12-11698 FAKQEDGGLLRDYFAKEQDGGLLRDYFAKDEGLLRDYFAMDGGLLRDYFAKEQDGGLLRD

:. :**** .****::**** ****: * *** ***:*: * ** .

Lu14-5765 YFAKEQDRGRRSLVTSPRN-----------------KMDVFCETTSASNWTEVLG---AH

Lu6-41637 YFAKQQDEGLRQGYFAKQQDGGLRQGYFAKEKDGGLRQGYFAKEKDGGLRQGYFAE--DG

Lu12-11761 YFAKQRNGGLLGDYFAKQE---------------------------------------DG

Lu12-11698 YFAKEQDGGLRQGYFAKQQDGGLRQGYFGKQQDGGLRQGYFGKQQDGGLRQGYFGKQHDR

****:.: * : .:

Lu14-5765 TLLKNRMEVSCETTSSRNMIKRDAEEGLANVVPLLMSQRDETTVE---AMDEE---IKGL

Lu6-41637 GLLRDYFAKEQDGGLRQGYFAKQQDEGLRQ--GYFAKQQDKGLRQGYFAKEKDGGLRQGY

Lu12-11761 GLLGDYFAKQRDGGLLRDYFAKQEDRGLLG--DYFAKQQDGGLLRDYFAKQEDGGLLGDY

Lu12-11698 GLRQGYFGKEQDGGLRQGYFGKQQDEGLRQ--GYFGKQQDGGLRQDYFSKQQDGGLRQGY

* . : . : .. : .: : ** : .*.* : ::: .

Lu14-5765 IDEKTKKLLR--------------------------------------------------

Lu6-41637 FAKEKDGGLRQGYFAEDGGLLRDYFAKEQDGGLRQGYFAKEKDGSLRQGYFVEDGGLLRD

Lu12-11761 FAKEHDGGFV-------------------------------------------------D

Lu12-11698 FSKQHDGGLRQG-----------YFSKQQDGGLRQGYFGKQQDGGLRQG-----------

: :: . :

Lu14-5765 LVLDERSTAVPREC---------------------------------------------R

Lu6-41637 YFAKEQDGGLRQGYFAKEKDGGLRQGYFAEDGGLLRDYFAKQQDGGLRQGYFAEDGGLLR

Lu12-11761 YFTKKHDGGEWAAFF---------------------------------------------

Lu12-11698 YFGKQRDGGLRQGYFGKQQDGGLRQDYFA-------------KDGGLRQDYFAKDGGLLG

. .:.. .

Lu14-5765 ELFWKMNS----------------------------------------------------

Lu6-41637 DYFAKQQDGGLSQGYFAEDEGLLRDYFAKEQDESLLRDHFAKEQDGGTIVSPPMFVLVGK

Lu12-11761 ----------------------------------------------------------GK

Lu12-11698 DYFAKKQDGG------------------------LLGDYFAKEQDGGMIVSSPMFMLVGK

Lu14-5765 --------

Lu6-41637 NQDEIPSN

Lu12-11761 QQDEKPSK

Lu12-11698 NQDEIPSN

Group 7

Lu11-24918 MAATSSLAVTTTLLATVGASKNRLPPTA--------------------------------

Lu10-34966 -MATSSLAMTTTLLATVGASKTISPPVAVSYKACGKRDGGYPPLSPLFGKKKWDVGYPPL

Lu11-28070 -MATSSLAMTTTLLATVGASKTRSPSTT--------------------------------

******:************. *..:

Lu11-24918 ------------------------------------------------------------

Lu10-34966 SPLFGQKKNDASYPPLSSLFGRKKRDVEYPSISPLLGREKKDAGYPPLSPLFGQKKGDTG

Lu11-28070 ---------------------------------------RRDFGYPPLNPLFGQKKGDTG

Lu11-24918 ----------SVSCKSYP-----------------------------GATNAVSYKARMK

Lu10-34966 YPPLSPLFGQEKSDDGYPPLSPLFGQEKDNTGYLPLSSLFGQKKSDAGYPPLSPLFGQDN

Lu11-28070 YPPLNPLFGQQKGDTGYPPLNPLFGEKKE----------------DTGYPSLHSLFGQKK

. . .** * . . .. :

Lu11-24918 GDAGYPPLSPLFGQNNEDAGYPPLSPLFGQEKGDAGVYGFIPHVFGKGQ-----------

Lu10-34966 GDAGYPPLSPLFGQEKSDAGYPPLSPLFGQEERDAG-YPPLSPLFGQDERDAGYPPLSPL

Lu11-28070 GDTGFPPLSPLFGQEKRDAGYPPLSPLFSQEKEDAG-YPPLSPLFGQEKGDADYPPLSPL

**:*:*********:: ***********.**: *** * :. :**: :

Lu11-24918 --VENGDAGVYGFIPHVFGKGQVENGDAGVYGFIPHVFGKGQV-----------------

Lu10-34966 FGQEKSDAG-YPPLSPLFGQ---EKRDAG-YPPLSPLFGQDEHDAGYPPLSPLYGRQPEG

Lu11-28070 FGQEKGDAG-YPPLSPLFGK---EKGDAG-Y---NGLFGKD-------------------

*:.*** * :. :**: *: *** * :**:.

Lu11-24918 ----------------------------------------ENGDAGVYGFIPH-------

Lu10-34966 DAGCVSPDFHMIFGNDAGWQCIFSYDKTGNAGWQCIFSYDKTGDAGNNGLFAKRQDGEVG

Lu11-28070 ------------------------------AGWQCIFSYDKTGDAGYNGLFGK-------

:.**** *:: :

Lu11-24918 ------VFGKGQV-ENGDAGVYGFIPH-------------VFGK---GQV----------

Lu10-34966 DAGYNGLFAKRQDGEVGDAGYNGLFAKRQDGEVGDAGYNGLFAKRQDGEVGDAGYNGLFA

Lu11-28070 DAGWQCIFSY--D-KTDDAGYNGLFGK-------DASWQCIFCY---DKVGDAGYNGLFA

:*. : .*** *:: : :* .:*

Lu11-24918 -----ENGDAGVYGFIPHVFGKGQVEKGDAGVYGFIPH-------------VFGKGQVEK

Lu10-34966 KRQDGEVGDAGYNGLFAKRQ-DGEV--GDAGYNGLFAKRQDGDVGDAGYNRLFAKRQDGE

Lu11-28070 KCHDGEVGDTGYYGLFAKCH-DGEV--GDTGYYGLFAKCHDGEVGDTGYYGLFVKCHDGE

* **:* *::.: .*:* **:* *::.: :* * : :

Lu11-24918 AGDAGSTVPYFPMIFGKDVGYIAHLIIFGNRS

Lu10-34966 VGDAG----YNGLFAKRQDGEVGDAGYNGLFA

Lu11-28070 VGDTG----YYGLFAKCHDDEVGDTGYYGLFA

.**:* * :: . :. * :

Group 8

Lu10-38024 MATSAFAFASTFRTPTAPIVSLPSAKNTTPA-----------------------AATVAR

Lu9-15288 MASSAFALASTFRVPTAPTVSLPSAKADHSVRNW--PVFGKADHSVRNWPVFGKADHSVR

Lu10-38063 MATIA------ARFPKHTT----GKGGDQSVNPWRFPIFGKTSHNSAG----GDHGYTSH

**: * * *. . . .. .

Lu10-38024 RFP----NHSAKRTLPMLAT-------VGDGGLFHHFFGKVDNNS------TVGDDGVFT

Lu9-15288 NWPVFGKADNSVSNWPVFGKAADDNSTRGDSGYVVPCFGKADNSHLHWPVFGKADNSHLH

Lu10-38063 NSP---GGDSSGNVLPCFAK-ADNSTGDCDGGYVLTCFAKAENST------GGGDAGYV-

. * .: * :.. *.* . *.*.:*. .* . .

Lu10-38024 F--FGKAKNDFT-------VGDGGF-FHYFGKIENNSIVDY-------DEGI--FTFFGK

Lu9-15288 WPVFGKADNSVRNWPVFGKANNSHLHWPVFGKEADNSVRNWPVFGKEADNSVCNWPVFGK

Lu10-38063 LPCFGKADNSTR-------GGDAGYVLSCFGK-ADNCTRGG-------DDGYI-LPCFGK

****.*. .:. *** :*. . *:. . ***

Lu10-38024 VENDSTVCDDGF-FHYFGNTDN-------------NSKGVDGGIGKADNN

Lu9-15288 ATDNSMGGDSGYVVPCFGKADNSHLHWPVFGKEADNSTGGDSGYVVPCFG

Lu10-38063 ADNSTAGGDGGYVLPCFGIADN-------------STAGGDGGYVRTCSS

. :.: *.*: . ** :** .: * *.* . .
