# Supplementary material for: In silico analysis reveals a multi-dimensional model of adaptive evolution in the flax orbitide-related precursor protein family
Source: Front Plant Sci. 2026 Jun 30;17:1824173. doi: 10.3389/fpls.2026.1824173 (PMC13365257; doi:10.3389/fpls.2026.1824173)
Supplement: Supplementary Table 1 — Repeat sections of 30 proteins. [file Supplementaryfile1.zip › Data S2_Domains identified in 6 proteins by Interproscan.docx]

Lu5-45630

Lu5-45630 contains a region identified to be homologous to the membrane-bound, organ-specific protein family (PF10950) with an E-value of 1.7E-8:


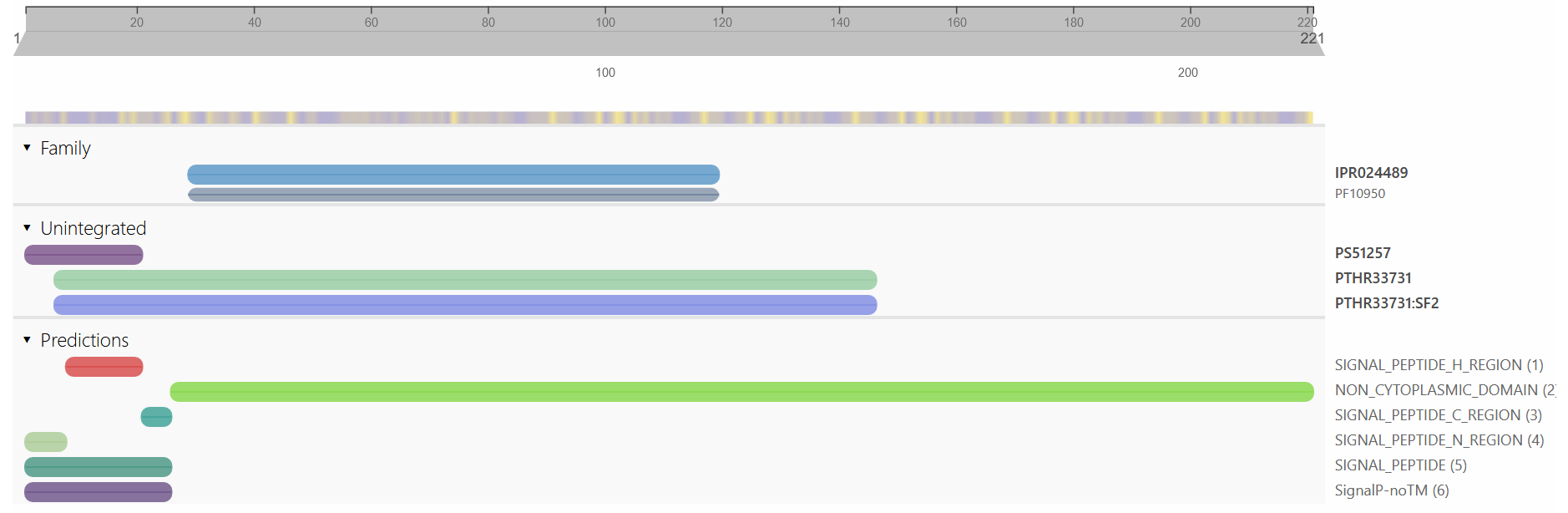


This region spans from the C-terminal end of the leader peptide to the second repeat motif. Alignment of Lu5-45630 to the profile HMM of PF10950 produces a consistent match:

# STOCKHOLM 1.0

g45630.t1 mapshlrflllalvllgagccsvngirk-DPiGDYWRSVMKDEPMPKAIQSLLLP--AANSDHQHPAVHYDTDHNFKN--PDQSQPDEHIFEYYSKDDAL-PSNKFAKGIGSRPDeaLLFYSKDD-----------------------alpsnkfakgigsrpdeallfyskddalpsnkfakgigsrpdeallfyskdd

#=GR g45630.t1 PP ***************************5.5559******************9872..222333334444443344444..33333444443333323222.234445555555531123333333.......................23333334444444456677777777788888888888888999********

#=GC PP_cons .............................55.9******************9872..222333334444443344444..33333444443333323222.23444555555553..23333333...........................................................................

#=GC RF ............................xxx.xxxxxxxxxxxxxxxxxxxxxxxxxxxxxxxxxxxxxxxxxxxxxxxxxxxxxxxxxxxxxxxxxxxxxxxxxxxxxxxxxxx..xxxxxxxxxxxxxxxxxxxxxxxxxxxxxxx....................................................

g45630.t1 alpsnkfakgigsrpdeallfyskddappsnkfakgigsrpdeallfysk

#=GR g45630.t1 PP **************************************************

#=GC PP_cons ..................................................

#=GC RF ..................................................

//

Although the matching region is conserved, only the fragment GDYWRSVMKDEPMPKAIQSLLL in the leader peptide is supported by high posterior probabilities ≥7, and the rest of region has only medium-low posterior probabilities ≤5. This renders the characterization of this putative domain inadequate confidence. According to the descriptions in the InterPro database, the plant organ-specific proteins consist of an N-terminal region followed by a tandem repeat region. The N-terminal region extends from the end of the signal peptide to the start of the repeat region, with a length ranging from 30 to 80 aa. The region of Lu5-45630 that matches the profile HMM of PF10950 before the repeat region has 67 aa, within the range of the characterized N-terminal region. The repeat motif of Lu5-45630 has 26 aa, which is also consistent with the repeat length range from 20 to 40 aa in these organ-specific proteins belonging to Type II proteins, a transmembrane protein category of which the C-terminal domain is targeted to the ER lumen. By piecing up the evidence from various perspectives we can more confidently speculate that Lu5-45630 is a member of the organ-specific protein family. This family includes a number of plant organ-specific proteins with unknown function but predicted to be exported and glycosylated (PMID: 2102854, Differential expression of two related organ-specific genes in pea). InterProScan also identifies the region from 6 aa to 146 aa matching a putative protein family in PANTHER with an E-value of 1.9E-12. We checked all the members in this family but unfortunately none is uncharacterized, so this match is not further investigated.

Lu8-3470

Same as Lu5-45630, a region homologous to the organ-specific protein family (PF10950) is identified in Lu8-3470. The homologous region extends from the end of the signal peptide to the third repeat motif. Alignment of Lu8-3470 to the profile HMM of the organ-specific protein family confirms the match reported by InterProScan with high (> 6) posterior probabilities. We thus have good confidence to accept the characterization by InterProScan.


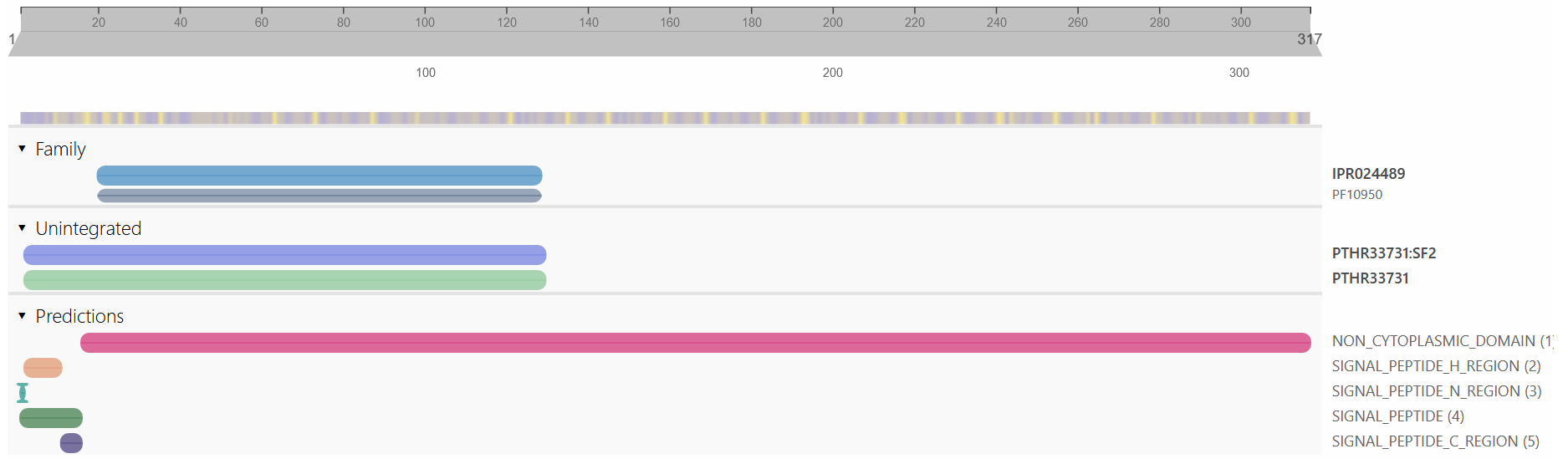


# STOCKHOLM 1.0

Lu8_127411 milmllqlgadsvnarkds--IGGYWKSVMKDQLMPKAIESLLPaanSDHADVRCDTNQVFQYHKDAVPSNEFSKEIESQPNQVfQYHK-DIVPSNE--FAEGTESQPNqVFQYHEDAVPSN---EFAKEIELQPN-----qvfqyhkdavpsnefakeielqpnqvfqyhkdavpsnefakeiesqpnqvfqyhkdvvt

#=GR Lu8_127411 PP ****************876..499********************4444444455555555555544555789********999856666.8877655..6699****986778888886554...35566665555.....678889*****************************************************

#=GC PP_cons .....................499********************...4444455555555555544555789********9998.6666.8877655..6699****98.778888886554...35566665555................................................................

#=GC RF ...................xxxxxxxxxxxxxxxxxxxxxxxxx...xxxxxxxxxxxxxxxxxxxxxxxxxxxxxxxxxxxxx.xxxxxxxxxxxxxxxxxxxxxxxx.xxxxxxxxxxxxxxxxxxxxxxxxxxxxxxx...........................................................

Lu8_127411 snefskgmelqpnqvfqyhkdivpsnefakgiesqpnqvfqyhkdvvpsnefskgigsqpnqvfqynkdvvpsnegfkeiesqpnqvfqyhkdavpsnefavgtetqpnqvfqyhkdvvpsnefakgies

#=GR Lu8_127411 PP **********************************************************************************************************************************

#=GC PP_cons ..................................................................................................................................

#=GC RF ..................................................................................................................................

//

Lu5-47766


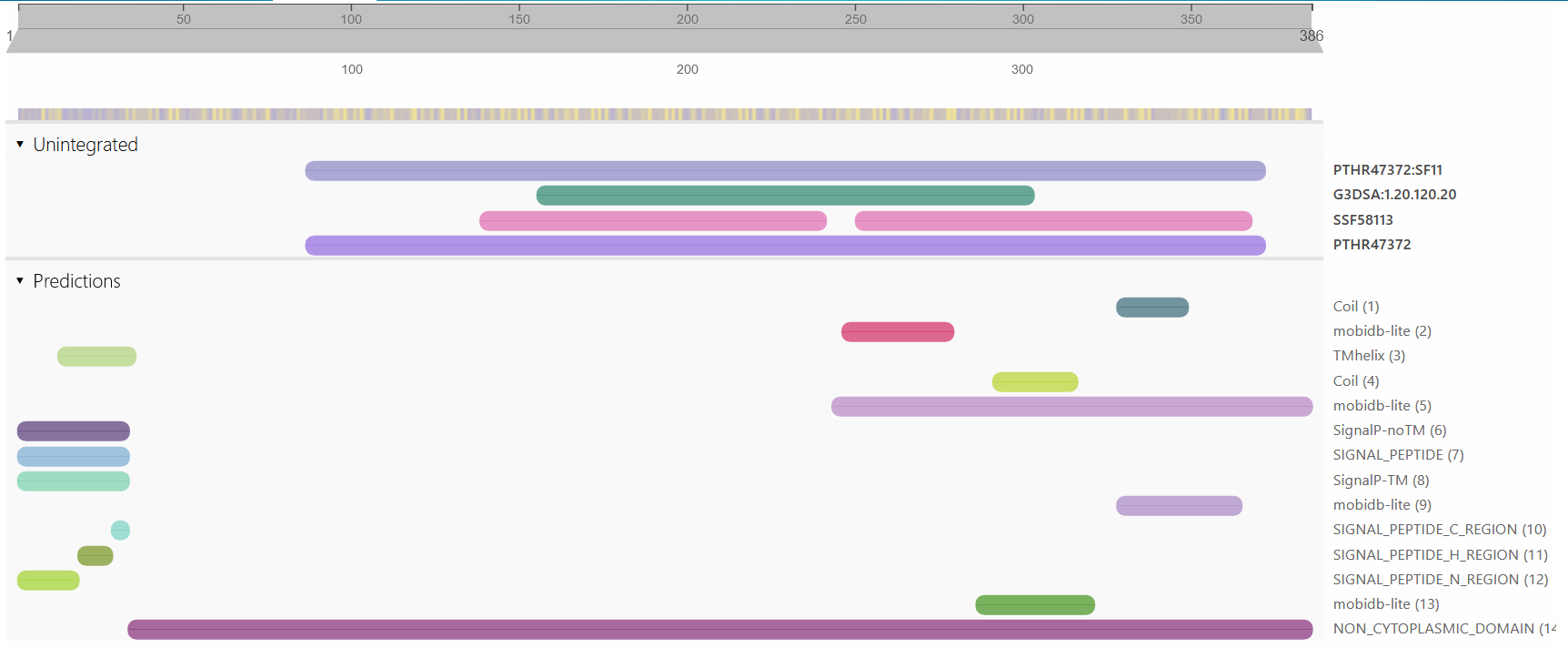


The repeat region of Lu5-47766 have two hits: one (87 – 372 aa) matches the DAUER up-regulated-related subfamily (PTHR47372:SF11) in the PANTHER database, the other has two sections (139 – 241 aa, 251 – 368 aa) matching the apolipoprotein (SSF58113) in the SUPERFAMILY database. As the PANTHER database classifies functionally related proteins based on scores against the Hidden Markov models (HMMs), the DAUER up-regulated-related subfamily (PTHR47372:SF11) contains 80 proteins of 39 species, most of which are plants. Sixty-nine of these 80 proteins are uncharacterized. All the remaining 11 proteins are assigned as late embryogenesis abundant (LEA) domain-containing proteins, among which 8 are from plant species. The phylogenetic tree of these 80 proteins provided by PANTHER shows that the 11 LEA proteins are mixed with instead of separated from those uncharacterized proteins, indicating that despite uncharacterized, these uncharacterized proteins share some important domains with the LEA proteins so as to belong to the same subfamily. Alignment of Lu5-47766 to the profile HMM of the DAUER up-regulated-related subfamily by *hmmalign* reveals a good match between the repeat region and the profile, with the majority of aligned sites supported by high consensus posterior possibilities (#=GC PP_cons ≥ 8, see the Stockholm alignment below). It is therefore with good confidence that Lu5-47766 is a member of the DAUER up-regulated-related subfamily, in which the repeat region contains LEA domains.

# STOCKHOLM 1.0

#=GS g47766.t1 DE CP027629.1_106420

g47766.t1 mamtmtmkvrdrglvtmmavvlclaltaevchgwgsdsdgpglwd--------------------------------------------------------K----TKETVSHAAGSVKEKTGDVVEgaqgwankH--------ISstmnlgadTTESVGAAGGDKAQGAKEIYRDARDKAYDTAGKVGSAGMDKAEDTK

#=GR g47766.t1 PP **********99999999999999999988888888888877764........................................................3....445566666666666666666554464331........22334456778889999999999999999999999999999999999999999999

#=GC PP_cons .....................................................................................................3....445566666666666666666........1........22........8889999999999999999999999999999999999999999999

#=GC RF .............................................xxxxxxxxxxxxxxxxxxxxxxxxxxxxxxxxxxxxxxxxxxxxxxxxxxxxxxxxxxxxxxxxxxxxxxxxxxxxxxxxxx........xxxxxxxxxxx........xxxxxxxxxxxxxxxxxxxxxxxxxxxxxxxxxxxxxxxxxxxxxx

#=GC MM .............................................----------------------------------------------------------------------------------........-----------........----------------------------------------------

g47766.t1 IKMSELGTAGQEKAGNAKEKVKHVAGEVGAAGRDKAEDAKEkvkhvagevgaasrDKAEDAKEKVKHVAGEVGAASRDKAEDAK----------EKVKHVAGEVGAASRDKAEDATAKLSAMGAAGRDKAEDAKEKVKHVAGEVGAASRDKAEDAKGKLSAMGAAGREKAEDAKEKVKQATVNVRDKAEEAKEKAKHVAG

#=GR g47766.t1 PP 999998777799999999999998888888888888888866666666666677778888888888888888888888888888..........8888888888888889***99999999999999********************************9777777**********************************

#=GC PP_cons 99999877779999999999999888888888888888886..............78888888888888888888888888888..........8888888888888889***99999999999999********************************9777777**********************************

#=GC RF xxxxxxxxxxxxxxxxxxxxxxxxxxxxxxxxxxxxxxxxx..............xxxxxxxxxxxxxxxxxxxxxxxxxxxxxxxxxxxxxxxxxxxxxxxxxxxxxxxxxxxxxxxxxxxxxxxxxxxxxxxxxxxxxxxxxxxxxxxxxxxxxxxxxxxxxxxxxxxxxxxxxxxxxxxxxxxxxxxxxxxxxxxxx

#=GC MM -----------------------------------------..............-------------------------------------------------------------------------------------------------------------------------------------------------

g47766.t1 DVGAAANDKAED-GKEKVSQAAEDARANGGEKAEGAKD---------IYGKVKEKLSQAT-----------gigtkskdekggel

#=GR g47766.t1 PP ************.6*******************99999.........5555555533333...........24444555667899

#=GC PP_cons ************.6*******************99999.........5555555533333.........................

#=GC RF xxxxxxxxxxxxxxxxxxxxxxxxxxxxxxxxxxxxxxxxxxxxxxxxxxxxxxxxxxxxxxxxxxxxxxx..............

#=GC MM ----------------------------------------------------------------------...............

//

Lu5-47766 also matches the profile HMM of the Apolipoprotein A-I superfamily (SSF58113). Consistent with the InterProScan result, there are two matching fragments in the repeat region, each spanning 3 linusorb-like repeat motifs (see the Stockholm alignment below). However, the consensus posterior possibilities (#=GC PP_cons ≤ 4) are lower than those of the alignment with the LEA domains of the DAUER up-regulated-related subfamily, indicating that the conservation is less reliable than that to the latter. Additionally, although the C-terminus region of Lu5-47766 also matches the profile HMM even with higher consensus posterior possibilities, it is worth noting that the conservation is not as strong as the two fragments in the repeat region, as indicated by the lower-case residues in the sequence. Given the above analyses, our speculation is that the linusorb-like repeat motifs in Lu5-47766 are LEA domains of the DAUER up-regulated-related subfamily.

# STOCKHOLM 1.0

#=GS g47766.t1 DE CP027629.1_106420

g47766.t1 mamtmtmkvrdrglvtmmavvlclaltaevchgwgsdsdgpglwdktketvshaagsvkektgdvvegaqgwankhisstmnlgadttesvgaaggdkaqgakeiyrdardkaydtagkvgsagmdkaedtkikmselgtagqekagnakekvkhv--------------------------------------------

#=GR g47766.t1 PP *******************************************************************************************************************************99999999999988877776655444444............................................

#=GC PP_cons ........................................................................................................................................................................................................

#=GC RF ............................................................................................................................................................xxxxxxxxxxxxxxxxxxxxxxxxxxxxxxxxxxxxxxxxxxxx

g47766.t1 --------------AGEVGAAGRDKAEDAKEKVKHVAGEVGAASRDKAEDAKEKVKHVAGEVGAASRDKAEDAKEKVKHVAGEVGaasrdkaEDATAKLSAMGAAGRDKAEDAKEKVKHVAGEVGAASRDKAEDAKGKLSAMGAAGREKAEDAKEKVKQA----tvnvrdkaeeakekakhvagdvgaaandkaedgkek

#=GR g47766.t1 PP ..............44444444444444444444444444444444444444444444444444444444444444444444444333333322222333333333333333333333333333333333333333333333333333333333333332....233344444444444444444444444444444444

#=GC PP_cons ..............44444444444444444444444444444444444444444444444444444444444444444444444.......22222333333333333333333333333333333333333333333333333333333333333332........................................

#=GC RF xxxxxxxxxxxxxxxxxxxxxxxxxxxxxxxxxxxxxxxxxxxxxxxxxxxxxxxxxxxxxxxxxxxxxxxxxxxxxxxxxxxxx.......xxxxxxxxxxxxxxxxxxxxxxxxxxxxxxxxxxxxxxxxxxxxxxxxxxxxxxxxxxxxxxxxxxxxxxxx....................................

g47766.t1 vsqaaedaranggekaegakdiygkvkeklsqatgigtkskdekggel

#=GR g47766.t1 PP 4455555556677888888899999999999*****************

#=GC PP_cons ................................................

#=GC RF ................................................

//

Lu6-41637


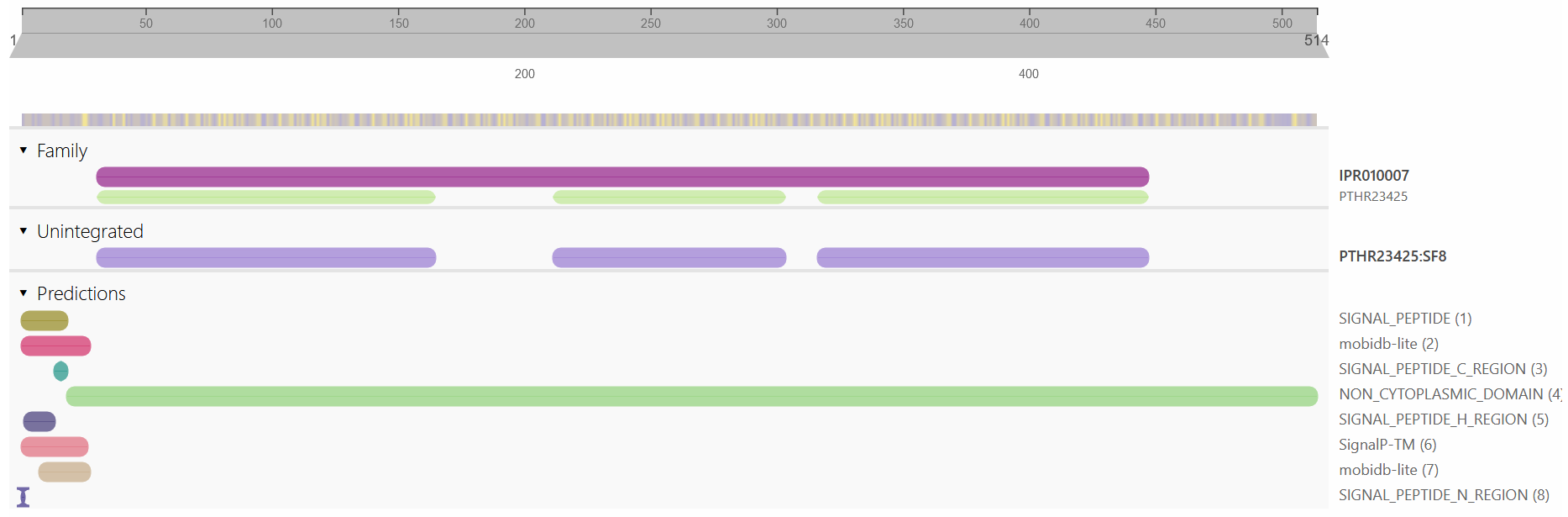


InterProScan finds matches from two protein family libraries in the region spanning from the end of the signal peptide to the fifth last linusorb-like repeat motif: one is the SPAN-X family from the InterPro library (IPR010007, equivalent Pfam ID: PF07458), matching the entire region specified (31 – 447 aa); the other is the nucleoporin AMO1-like family from the PANTHER library (PTHR23425), matching 3 fragments in the specified region, namely 31 – 164 aa spanning from the end of the signal peptide to the first 9 repeats, 212 – 303 aa spanning from the 14th to the 20th repeat, and 317 – 447 aa spanning from the 23rd to the 33rd repeats (see the Stockholm alignment below). Alignment of Lu6-41637 to the profile HMM of the SPAN-X family by *hmmalign* shows a low conservation with the profile, as there is no capital letter in the sequence. Plus, many of the columns are supported by high posterior probabilities (> 6), meaning the poor match is of good certainty.

# STOCKHOLM 1.0

Lu6_4193 massfampaaatfssvassppqhskknmttmltaslktsskvsvsaavvsfnkqqdggirrtyfakqqdgglrqgyfakekdgglrqgyfaedggllrdyfakeqdgglrqgyfakekdgglrqdyfaedggllrdyfakeqdgglrqgyfakekdgglrqdyfaedggllrdyfakeqdgglrqgyfakekdgglrqdy

#=GR Lu6_4193 PP ********************************************************************************************************************************************************************************************************

#=GC PP_cons ........................................................................................................................................................................................................

#=GC RF ........................................................................................................................................................................................................

#=GC MM ........................................................................................................................................................................................................

Lu6_4193 faedggllrdyfakeqdgglrqgy----------------------------------------FAKQQDEGLRQGYFAKEKDGGLRQGYFAKEKDGGLRQGYF-AEDGGLLRDYFAKEQDGG-LRQGYFAKEKDGGLRQGYFAE-DGGLLRD-YFAKEQDGGLRQGYFAKQQDKGLRQGYFAKEKDGGLRQGYFAKEKD

#=GR Lu6_4193 PP **9999999999887766665555........................................5555666667788888888888888888888888888888.777777777777777776.455888888888888877754.3444444.6777777778888888888888888888888888888888888888

#=GC PP_cons ................................................................5555666667788888888888888888888888888888.777777777777777776.455888888888888877754.3444444.6777777778888888888888888888888888888888888888

#=GC RF ........................xxxxxxxxxxxxxxxxxxxxxxxxxxxxxxxxxxxxxxxxxxxxxxxxxxxxxxxxxxxxxxxxxxxxxxxxxxxxxxxxxxxxxxxxxxxxxxxxxxxxxxxxxxxxxxxxxxxxxxxxxxxxxxxxxxxxxxxxxxxxxxxxxxxxxxxxxxxxxxxxxxxxxxxxxxxxxxxx

#=GC MM ........................--------------------------------------------------------------------------------------------------------------------------------------------------------------------------------

Lu6_4193 GSLRQGYFVEDGgllrdyfakeqDGGLRQGYFAKEKdGSLRQG-----------------------yfvedggllrdyfakeqdgglrqgyfakekdgglrqgyfaedggllrdyfakqqdgglsqgyfaedegllrdyfakeqdesllrdhfakeqdggtivsppmfvlvgknqdeipsn

#=GR Lu6_4193 PP 8888887666666666666666655666666666666666655.......................46677888888888888888888899999999999999*****************************************************************************

#=GC PP_cons 888888766666...........5566666666666.666655..........................................................................................................................................

#=GC RF xxxxxxxxxxxx...........xxxxxxxxxxxxx.xxxxxxxxxxxxxxxxxxxxxxxxxxxxx...................................................................................................................

#=GC MM ------------...........-------------.----------------------------....................................................................................................................

//

When Lu6-41637 is aligned to the profile HMM of the nucleoporin AMO1-like family by *hmmalign*, the matching region differs from that output by InterProScan, in that there is only one consecutive region spanning 12 repeats from the 15th to the 26th repeat, with most of the posterior probabilities >6. The discrepancy in the matching region between InterProscan and hmmalign is due to the repetitive nature of this region, making the alignment “slippery”, i.e. aligning any repeat to the profile can achieve similar scores. Therefore, we can confidently assume Lu6-41637 as a member of the nucleoporin AMO1-like family which consists of 31 genes in 7 species including *Gossypium hirsutum* (upland cotton) as the only plant species. We found that the *Gossypium hirsutum* nucleoporin AMO1-like protein (A0A1U8KIH9) is highly repetitive, as identified by RADAR. Interestingly, although Lu6-41637 is less conserved with the above SPAN-X family than with the nucleoporin AMO1-like family, we found that the nucleoporin AMO1-like family (PTHR23425) is actually integrated to the SPAN-X family in the InterPro library (IPR010007). The reason may be that SPAN-X stands for “Sperm Protein Associated with the Nucleus on the X chromosome”, and nucleoporins are the building blocks of the nuclear pore complex that transport molecules across the nuclear envelope at a high rate (*Doye V, Hurt E (June 1997). "From nucleoporins to nuclear pore complexes". Current Opinion in Cell Biology.* ***9*** *(3): 401–11.*). Given these knowledge facts, we characterize Lu6-41637 as a nucleoporin AMO1-like protein pertaining to the larger SPAN-X family.

Lu10-34966

InterProScan identifies a nucleoporin FG repeat (IPR025574 in InterPro or PF13634 in Pfam) in the repeat region of Lu10-34966. The match spans 6 repeats from the 7th to the 12th repeat motif (126 – 224 aa). Alignment of Lu10-34966 to the profile HMM of Nucleoporin FG repeat generates a consistent match spanning from the 7th to the 9th repeat motif, shorter than the InterProScan output (see the Stockholm alignment below). However, the match is supported by low posterior probabilities < 5. While the surrounding regions have much higher posterior probabilities, they are not determined as match to the profile HMM. Thus, there is little confidence about the repeat region of Lu10-34966 characterized as the Nucleoporin FG repeat. This Nucleoporin FG repeat is reported to occur mostly in fungi and metazoa, with only 13 plant species of the total 832 species containing this repeat domain. We speculate that the reason why InterProScan matches it to Lu10-34966 may be the repetitive signature FG shared by both proteins, as shown in the HMM logo of Nucleoporin FG repeat.


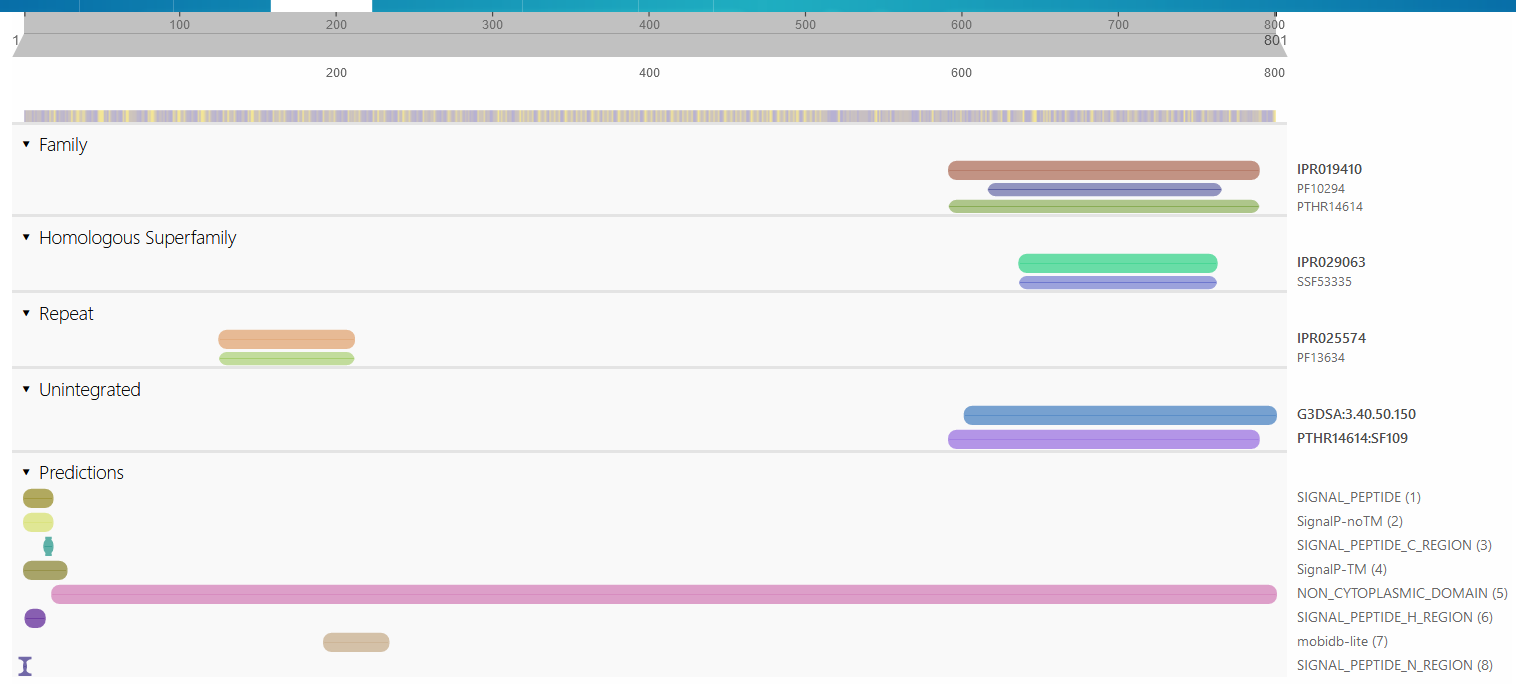


# STOCKHOLM 1.0

Lu10_4234 matsslamtttllatvgasktisppvavsykacgkrdggypplsplfgkkkwdvgypplsplfgqkkndasypplsslfgrkkrdveypsispllgrekkdagypplsplfgqkkgdtgypplsplfgqekdntgylpls------------SLFGQKKSDaGYPPLSPLFGQDNGDAGYPPLSPLFGQ-----------

#=GR Lu10_4234 PP *************************************************************9999999999999999999999999999999999999998888888876666666666666655333333333333333............3333333332222222233322222222222223333...........

#=GC PP_cons ........................................................................................................................................................333333333.222222233322222222222223333...........

#=GC RF ............................................................................................................................................xxxxxxxxxxxxxxxxxxxxx.xxxxxxxxxxxxxxxxxxxxxxxxxxxxxxxxxxxxxx

Lu10_4234 --EKSDAGYPPLSPLFG----------------qeerdagypplsplfgqderdagypplsplfgqeksdagypplsplfgqekrdagypplsplfgqdehdagypplsplygrqpegdagcvspdfhmifgndagwqcifsydktgnagwqcifsydktgdagnnglfakrqdgevgdagynglfakrqdgevgdagyn

#=GR Lu10_4234 PP ..222222222222222................2223333333333333555555555555555577777778888888889999999999999999*******************************************************************************************************

#=GC PP_cons ..222222222222222.......................................................................................................................................................................................

#=GC RF xxxxxxxxxxxxxxxxxxxxxxxxxxxxxxxxx.......................................................................................................................................................................

Lu10_4234 glfakrqdgevgdagynglfakrqdgevgdagynglfakrqdgevgdagynglfakrqdgevgdagynglfakrqdgevgdagynglfakrqdgdvgdagynrlfakrqdgevgdagykglfakrqdgevgdagykglfakrqdgevgdagynglfa

#=GR Lu10_4234 PP *************************************************************************************************************************************************************

#=GC PP_cons .............................................................................................................................................................

#=GC RF .............................................................................................................................................................

//

Lu13-23576

The majority of the protein, spanning from the leader peptide to the last repeat motif even followed by a short vestigial fragment with similar repeat pattern, is identified homologous to the C-terminal domain of serralysin-like metalloprotease (IPR011049 in InterPro library and G3DSA:2.150.10.10 in CATH-Gene3D database) with an E-value of 1.2E-5. The C-terminal domain of serralysin-like metalloprotease is a beta-helix structure with metal-binding activity. According to InterPro’s description, serralysin is a bacterial Zn-endopeptidase including the astacin family, snake venom Zn-endopeptidases, the extracellular metalloproteases from Serratia sp., Pseudomonas sp. and Erwinia sp., and the matrixins. It acts as a virulence factor causing tissue damage and anaphylactic response (Mechanistic studies of the astacin-like Serratia metalloendopeptidase serralysin). The serralysin precursor lacks a signal peptide but employs the C-terminal domain for secretion. This is consistent with the InterProScan result of Lu13-23576 in which no signal peptide is predicted. Alignment of Lu13-23576 to the profile HMM of the C-terminal domain of serralysin-like metalloprotease (see the Stockholm alignment below) shows only a short fragment (62 – 96 aa) is considered as match state but with low posterior probabilities (< 5), and the larger region reported by InterProScan turns out to be insert state with high posterior probabilities (> 6). This associated with the E-value of 1.2E-5 suggests that the characterization of Lu13-23576 as a homologue of the C-terminal domain of serralysin-like metalloprotease is not reliable enough to accept.


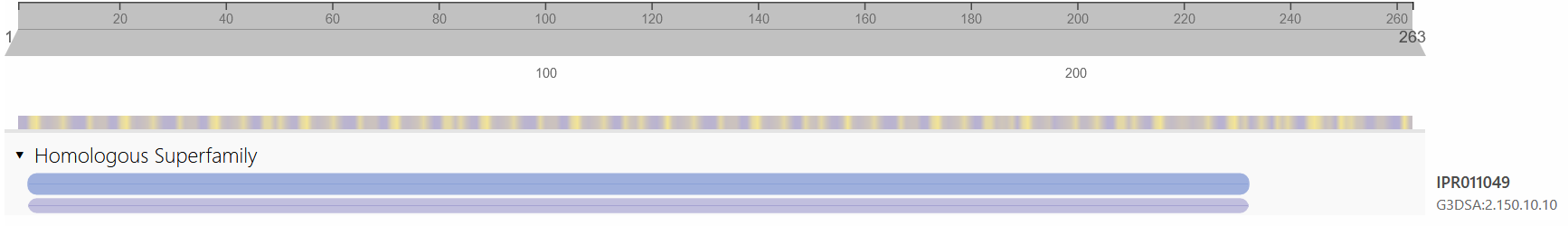


# STOCKHOLM 1.0

Lu13-118310 mfgkahqdgsvfvgqsslfgkahqdgsvfvgqsslfgkahqdgslfggqgamfgkahqdgs-----------------------------------------------------------------------------------------VFVGQSSLFgkTHQDGSLFGGQGAMFGKAHQDGSV---------------

#=GR Lu13-118310 PP ***9999888888888888877776666665555555555555544444444444443333.........................................................................................44444444444444444444444444444444433...............

#=GC PP_cons ......................................................................................................................................................444444444..444444444444444444444433...............

#=GC RF .............................................................xxxxxxxxxxxxxxxxxxxxxxxxxxxxxxxxxxxxxxxxxxxxxxxxxxxxxxxxxxxxxxxxxxxxxxxxxxxxxxxxxxxxxxxxxxxxxxxxxx..xxxxxxxxxxxxxxxxxxxxxxxxxxxxxxxxxxxxxxx

Lu13-118310 --------------------------------------------------------------fvgqsslfgkahqdgslfgdqgamfckahqdgsvfvgqsslfgkahqdgslfgdqgamfckahqdgsvfvgqsslfgkahqdgslfggqdarfgkahqdvhqdghhdgslfggqgamfgkahqdahqdgtlfgkarve

#=GR Lu13-118310 PP ..............................................................34444555556666667777777777777777777777777777777888888888888888888888888888888888888888888888888888889999999999999999999999999999999*******

#=GC PP_cons ........................................................................................................................................................................................................

#=GC RF xxxxxxxxxxxxxxxxxxxxxxxxxxxxxxxxxxxxxxxxxxxxxxxxxxxxxxxxxxxxxx..........................................................................................................................................

Lu13-118310 gdlgdgslfgkgqdvkrgdnfvaapilke

#=GR Lu13-118310 PP *****************************

#=GC PP_cons .............................

#=GC RF .............................

//
