# Supplementary material for: In silico analysis reveals a multi-dimensional model of adaptive evolution in the flax orbitide-related precursor protein family
Source: Front Plant Sci. 2026 Jun 30;17:1824173. doi: 10.3389/fpls.2026.1824173 (PMC13365257; doi:10.3389/fpls.2026.1824173)
Supplement: Supplementary Table 1 — Repeat sections of 30 proteins. [file Supplementaryfile1.zip › Data S3_Amino acid compositions.docx]

Amino acid composition of the flax proteome:

| Amino Acid | Count | Frequency (%) |
| --- | --- | --- |
| A | 13099 | 5.65 |
| C | 3563 | 1.54 |
| D | 10674 | 4.60 |
| E | 14526 | 6.27 |
| F | 8206 | 3.54 |
| G | 11748 | 5.07 |
| H | 5399 | 2.33 |
| I | 10169 | 4.39 |
| K | 10615 | 4.58 |
| L | 16504 | 7.12 |
| M | 4186 | 1.81 |
| N | 8062 | 3.48 |
| P | 10080 | 4.35 |
| Q | 9216 | 3.98 |
| R | 12300 | 5.31 |
| S | 15216 | 6.56 |
| T | 11457 | 4.94 |
| V | 12530 | 5.41 |
| W | 2784 | 1.20 |
| Y | 6005 | 2.59 |

Amino acid composition of the 300 generated sequences (total 3000 residues):

| Amino Acid | Count | Frequency (%) |
| --- | --- | --- |
| A | 169 | 5.63 |
| C | 47 | 1.57 |
| D | 139 | 4.63 |
| E | 186 | 6.20 |
| F | 108 | 3.60 |
| G | 151 | 5.03 |
| H | 71 | 2.37 |
| I | 133 | 4.43 |
| K | 136 | 4.53 |
| L | 215 | 7.17 |
| M | 53 | 1.77 |
| N | 105 | 3.50 |
| P | 130 | 4.33 |
| Q | 120 | 4.00 |
| R | 158 | 5.27 |
| S | 198 | 6.60 |
| T | 147 | 4.90 |
| V | 163 | 5.43 |
| W | 35 | 1.17 |
| Y | 79 | 2.63 |
