# Supplementary material for: In silico analysis reveals a multi-dimensional model of adaptive evolution in the flax orbitide-related precursor protein family
Source: Front Plant Sci. 2026 Jun 30;17:1824173. doi: 10.3389/fpls.2026.1824173 (PMC13365257; doi:10.3389/fpls.2026.1824173)
Supplement: Supplementary Table 1 — Repeat sections of 30 proteins. [file Supplementaryfile1.zip › Data S5_Gene conversion test results.docx]

Group 1

CLUSTAL W (1.81) multiple sequence alignment

G11-516P ATGGCTGTTGTGTCCTCTCTGGCTCTGACCACT---AGCCTAGTTGCTACCGCCGCCGGC

G11-514P ATGGCTGCT---TCCTCTGTCCCTCTGACCACC---AGCCTAGTTGCTACCGCCGCCGCC

G14-170N ATGGCTGCTGCTTCCTCTCTCGCTCTGGCCACCGCTAGCCTAGTTGCTACCGGCGCCGGC

******* * ****** * ***** **** **************** ***** *

G11-516P CGTAATAATAATGCCTTCCCACCATCCTCCTCCAGGAACAACAAGGCACCAGCAGACCTT

G11-514P GGCCGGAACAAC------------------------AATAGCAAGACACCAGCAAACCTT

G14-170N GGCCGTAATAACGCCTTCCTACCCTC---------GAAGAACAAGACAC---CAAACCTT

* ** ** ** * **** *** ** *****

G11-516P TTCATTACTCCCAAGACAACAA------CAACAGTGAAAGCAGCAGCTGTC---------

G11-514P TTCCTTACTCCCAAAACA---------TCAACAGTGAA---AGCTGCCGTC---------

G14-170N TTCCTTAATCCCAACAAAACAACGTCGTCAACAGTGAA---AGCTGTTGTCTCATCATCA

*** *** ****** * * ********** *** * ***

G11-516P TCATGCAAACGTCCCTACCCGAAAGGAG--------------------------------

G11-514P TCATGCAAACTTTC----------------------------------------------

G14-170N TCATGCAAACGCCCCTACCCGAAAGGAGATGCTAGTTTATTCTTGGGTATTGATGATGTA

********** *

G11-516P ------------------------------------------------------------

G11-514P ------------------------------------------------------------

G14-170N TTCGGAAAGGATGCTGTTGCTGGCCATGATAATGATCAGGATGGTTTGTTGTTTCCACTC

G11-516P ------------------------------------------------------------

G11-514P ------------------------------------------------------------

G14-170N TTGCTTTTTATATTGGGGATGGCGAGAACAAGGTGTAGGAAATTGTTTAGATATCGTTTA

G11-516P ------------------------------------------------------------

G11-514P ------------------------------------------------------------

G14-170N GATGCATATTAACTAATCCCATCATTATATCTAACTTTCTTATATCTTTCTTATATAAAT

G11-516P ------------------------------------------------------------

G11-514P ------------------------------------------------------------

G14-170N CAATAACTTTCTTATATAAATCAATAACAAAGGTTTTTAGTACTAATCAATGATTAGTAT

G11-516P ------------------------------------------------------------

G11-514P ------------------------------------------------------------

G14-170N TTGCTGAAGCCTTTGGTTTAATGACTAGTACTTGCTGAAGCCTTTAGATTGATTACGACT

G11-516P -----------CAGTTGCTGCTGCT-----------------------------------

G11-514P --------------GGGTAGCCACC-----------------------------------

G14-170N TGTGAGAATTTCATGTGTAGCTTCTTTTTTCAGTTTACGCTAATTGGATTTTGGATTTTC

* ** *

G11-516P ------------------------------------------------------------

G11-514P ------------------------------------------------------------

G14-170N TTTGTCAATACTGGCTAAAACGTTTGATCGAAAAACGATTTATCAAAGTATTTGGTAATT

G11-516P ------------------------------------------------------------

G11-514P ------------------------------------------------------------

G14-170N AGGGTTTTCTTTTAAAAGTTTTTAATGGCTTCCTAATTCAGTTTTAGATAAACTATTACA

G11-516P ------------------------------------------------------------

G11-514P ------------------------------------------------------------

G14-170N ACTAACCATCAATTTTGGATAAACTATTACAACTAACCATCAATTTTAGATAAACTATTA

G11-516P ------------------------------------------------------------

G11-514P ------------------------------------------------------------

G14-170N CAACTAACCATCAGTTGTAGATAAACTATTACAACTAACCATCAGTTGTAGATAAACTAT

G11-516P ---------------------------------------------------------ACT

G11-514P ---------------------------------------------------------ACC

G14-170N TACAACTAACCATCAGTTGTAGATAAACTATTACAACTAACCATCAGTTGTAGATAAACT

**

G11-516P AGTAC--CTTGTCTCCTATTTCTGGA----------------------------------

G11-514P ACCAC--C----------------------------------------------------

G14-170N ATTACAACTAACCCTCTATTTATAGAATTTCTCATAAACTTTCACCCTATTTGACCATCA

* ** *

G11-516P ------------------------------------------------------------

G11-514P ------------------------------------------------------------

G14-170N ACTCATTAAGCTAATCCATTTACATTAATCCGGTCCATACTACTAAAAAAGTGTGTGTCC

G11-516P ------------------------------------------------------------

G11-514P ------------------------------------------------------------

G14-170N ATATTACTAAAAAAGCGTGTGAAAGTGTGTGACTTTGTAGGACCCGATTCGATTAGTCGT

G11-516P ------------------------------------------------------------

G11-514P ------------------------------------------------------------

G14-170N GGTCCAAACTACTAATTAACATTGACCTCTAATAAGATGTGTTAACTCCTAACTGGACCG

G11-516P ------------------------------------------------------------

G11-514P ------------------------------------------------------------

G14-170N AATTACTTTTGATTAATCAGCCTCCCTAGTTTTTATTCGGATTCGGATTTAGGCCGAAGG

G11-516P ---------------AAGGATGGCGGCCTCCGCAACCAGGAGGAG---AGCGATGGTATG

G11-514P ----------------------------------ACCAGG--------------------

G14-170N ACATAAATTCTTCACAATGATGCAGCTGCAAGTGGCCAGGAGATGGCCGCCGATGATATG

*****

G11-516P TTGGTCTTCCCCTTATTTATATTCGGCAAGGAAGGTAGTCAGGACAAGTATAATGGAGCA

G11-514P ------------------------------------------------------------

G14-170N TTGATGCCATTCTTTTGGATATTCGGAAAAGAAGGACAGCAGCAGGAG------------

G11-516P GCTGCCCTCCGCGACCAGGAGGAGAGCGATGGTATGTTGATCCCCCCCTTCTTTGTCATA

G11-514P ----------------AAGAGGGGAGCGGCGG----------------------------

G14-170N ------------GCCGAGGAGAGCAGCGATGATATGTTGAT---GCCATTCTTTTGGATA

* *** **** *

G11-516P TTCGGCAAGGAAGGTTGTCAGGATATCGGCCACAAGTATAATAATGC-------------

G11-514P ------------------------------------------------------------

G14-170N TTCGGCAAGGAAGGACAGCAGCAGGAGGCCGAGAGCAGCGATGATATGTTGCTGCCATTC

G11-516P ----------------------CGCAGCAGCTGGCGCCCTCCGCGACCAGGAGGAGAGCG

G11-514P -------------------------------------------------------CGGCG

G14-170N TTTTGGATATTCGGCAAGGAAGGACAGCAGCAGGAGGCC-----------GAGAGCAGCG

***

G11-516P ATGGTATACTGGTCCCCCCCTTCTTTCTCATATTCGGCAAGGAAGGTAGTCAGGACAAGT

G11-514P ATGATATGTTGAAGCCCTTCTTCTTTTGGATATTCGGC----------------------

G14-170N ATGATATGCTGATGCC---TTTCTTTTGGATATTCGGCAAGCA-----------------

*** *** ** ** ****** *********

G11-516P ATAATGCAGCAGCAGCTGG-----------------------------------------

G11-514P ------------------------------------------------------------

G14-170N -----GCAGCAGCAGCAGGGTGAGAGCAGCGATGATATGTTGATGCCTTTCTTTTGGGTA

G11-516P --CGGCCTCCGCGGCAAGGAGCAGCAGGGTGACAAGATGGCGGCTGGAGCTGAGAATTAG

G11-514P ---------------------------------------------------------TAG

G14-170N TTCGGCAAGCAAGGTGACAACAACAAGGGCGATGCTGTAGAAGCAATCCTTAAGAACTAG

***

# Output of GENECONV for sequence file G11-516P_G11-514P_G14-170N.clw

# GENECONV version 1.81

# Command line: GENECONV G11-516P_G11-514P_G14-170N.clw /r /w123 /lp

# -outpath=D:\BaiduSyncdisk\PhD\Software\geneconv

#

# G11-516P_G11-514P_G14-170N.clw: 3 sequences:

# CLUSTAL W (1.81) multiple sequence alignment

#

# Options and features: 3 DNA sequences, SILENT SITES only,

# 1680 aligned bases, 6 polymorphisms, CLUSTAL format,

# mismatches in fragments not allowed, no groups defined,

# maxSimPairPval=0.05, maxSimGlobPval=0.05, no maxKAPairPval limits,

# no maxKAGlobPval limits, simPvals based on 10,000 permutations,

# global P-values based on BLAST-like global scores,

# fragment limits: minlength=1, minnpolys=2, minscore=2,

# pairwise max lists=2000, starting seed=123,

# sites with indels are skipped,

# no match (replacement) char in source file.

# Sequence names: G11-516P G11-514P G14-170N

#

# No mismatches in fragments (`gscale'=0)

# Nucleotide codes in data: T(682) C(549) A(685) G(532) Other(2592)

# CG/TCAG ratio: 44.16%

# Non-nucleotide characters in data: -(2DH)(n=2592)

# 1456 sites with missing data or unrecognized characters.

# No sites with all gap or unrecognized characters.

# 59 polymorphic and 165 monomorphic sites with no indels.

# 495 codon positions with indels or missing data.

# 29 amino-acid polymorphic codon positions with no indels.

# 6 silent codon polymorphisms to be permuted.

# CODON POSITIONS with unknown or missing data are excluded.

# The silent sites at 36 amino-acid monomorphic codon positions are

# 0-degen (2) 2-degen (12) 3-degen (0) 4-degen (18) irreg (4)

# The silent polymorphic sites from these codon positions are

# 0-degen (0) 2-degen (3) 3-degen (0) 4-degen (3) irreg (0)

# Codon usage for 3-fold degenerate codons: (Ile) ATT(2) ATC(0) ATA(2)

#

# Comparisons without any fragments (SILENT SITES):

# G11-516P v G11-514P (inner frags): All poly sites are different.

#

# The following are for outer fragments (SILENT SITES):

# G14-170N (outer sequence frags): No sites are unique.

#

# The starting random number seed is 123.

# Simulated P-values are based on 10,000 permutations.

# Maximum BLAST-like scores:

# Inner Max Sim S.D.s above S.D. of

# frags Score P-value sim. mean sims

# SCORE 0.118 1.0000 -1.26 0.3431

# OuterSeq

# frags 0.118 1.0000 -1.26 0.3431

#

# Global lists: no I and no O significant fragments

# Pairwise lists: no I and no O significant fragments

# where I means inner pair, O is outer seq, A is outer pair, and

# G means outer group (fragments).

# Global P-values are based on BLAST-like global scores.

#

# Fragment offsets and lengths are in nucleotides.

# Bases other than `TCAGU' are treated as indels

# for the purpose of generating polymorphisms.

#

#

# Global inner fragments (6 polymorphisms, 1680 aligned bases):

# (Inner fragments are runs of matching sites.)

# (Sim and BC KA P-values are corrected for multiple comparisons.)

# (Fragments are listed only if Sim P-value <= 0.05.)

# See earlier in the file for the full restrictions on fragments.

#

#

# No inner fragments listed.

#

#

# Global outer-sequence fragments (6 polymorphisms, 1680 aligned bases):

# (Outer-sequence fragments are runs of unique sites.)

# (Sim and BC KA P-values are corrected for multiple comparisons.)

# (Fragments are listed only if Sim P-value <= 0.05.)

# See earlier in the file for the full restrictions on fragments.

#

#

# No outer-sequence fragments listed.

#

#

# Pairwise inner fragments (6 polymorphisms, 1680 aligned bases):

# (Inner fragments are runs of matching sites.)

# (P-values are not corrected for multiple comparisons.)

# (Fragments are listed only if Sim P-value <= 0.05.)

# See earlier in the file for the full restrictions on fragments.

#

# No inner fragments listed.

#

#

# Pairwise outer-sequence fragments (6 polymorphisms, 1680 aligned bases):

# (Outer-sequence fragments are runs of unique sites.)

# (P-values are not corrected for multiple comparisons.)

# (Fragments are listed only if Sim P-value <= 0.05.)

# See earlier in the file for the full restrictions on fragments.

#

# No outer-sequence fragments listed.

#

Group 2

CLUSTAL W (1.81) multiple sequence alignment

G3-449N ATGGCTATTGCTTCCTCCACTTTCACCCTTGCTTTGCCCAGCCTAGGGTCATCTCCATCA

G4-136N ATG------GCTTCCTCCGCTTTCACCCTTGCTTTGCCCAGCTTAGGGTCATCTCCATCA

*** ********* *********************** *****************

G3-449N CCATTCAAGGGTCGTGCTCATATTGGACTTGCTCCCGTTCTTAAAGCTCGCAAAACCTCA

G4-136N CCCTTCAACGGTCGTGCTCATGTTGGACTTCCTCCCGTTCTTAAAGCTCGCAAAACCCC-

** ***** ************ ******** ************************** *

G3-449N GCGACTACCCTTTCTCGCGAAACCTTGATCAGTCACTCATCCAAACTTCATCACAGTCTC

G4-136N --------------------------GATCGTCAGCTCATCCAAACT---TCACAGTACC

**** ************ ******* *

G3-449N CTCAAGAAATC---------------------AGGTGATGCCGGCAT-------------

G4-136N CTCAAGAAACATGAGGTTGTTGACTCGGAGAGAGGTGATGCCGGCATTCCTCCCTTCTGG

********* ***************

G3-449N ------------------------------------------------------------

G4-136N TTAACCCTCGTGGGCAAGCAGCGCACTGATGTTCTCAACTCGAAGCTAGGTGATGCTGGC

G3-449N --------------------------------------AGGTGATGATGGCATTCCTCCC

G4-136N CTTCCTCCCATGTGGGTGGAGGTTTTTGGCTCAGAGAGAGGTGATGCCGGCATTCCTCCC

******** ************

G3-449N TTCTGGTTAACCCTCTTTGGCAAGCAGCAAGCTAATGTTTTCAACTCGGAGAAGGGTGAT

G4-136N TTCTGGTTAACCCTCGTTGGCAAGCAGCGCACTGATGTTTTCAACTCGAAGCTAGGTGAT

*************** ************ ** ************** ** ******

G3-449N GCTGGCATGGCTCCTATGTGGGTGACGGTTTTTGGCTCAGAGAGAGG-------------

G4-136N GCTGGCCTTCCTCCCATGTGGGTGGAGGTTTTTGGCTCAGAGAGAGGTACTGATGCCGGC

****** * **** ********* *********************

G3-449N --------------------------------------------TGTTTTTAACTCGGAG

G4-136N ATTCCTCCCTTTTGGTTAACCCTCGTTGGCAAGCAGCATGCTAATGTTTTCAACTTAGAG

****** **** ***

G3-449N AAGGGTGATGCTGGCATGGCTCCCGTGTGGGTGACGGTTTTTGGCTCAGAGAGAGG----

G4-136N AAGGGTGATGCTGGCCTTCCTCCCATGTGGGTGGAGGTTTTTGGCTCAGAGAGAGGTACT

*************** * ***** ******** *********************

G3-449N -----------------------------------------------------TGTTTTT

G4-136N GATGCCGGCATTCCTCCCTTTTGGTTAACCCTCGTCGGCAAGCAGCATGCTAATGTTTTC

******

G3-449N AACTCGGAGAAGGGTGATGCTGGCATGGCTCCCGTGTGGGGTGACGGTTTTTGGCTCAGA

G4-136N AACTCAGAGAAGGGTGATGCTGGCCTTCCTCCCATGT-GGGTGGAGGTTTTTGGCTCAGA

***** ****************** * ***** *** ***** ***************

G3-449N GAGAGGTGTT--------------------------------------------------

G4-136N GAGAGGTACTGATGCCGGCATTCCTCCCTTCTGGTTAACCCTCATCGGCAAACATGCTGG

******* *

G3-449N -----------------------------TTTAA

G4-136N TCAGATTGTTGACTCAACCAGCGTTAACACCTGA

* *

# Output of GENECONV for sequence file .\G3-449N_G4-136N.clw

# GENECONV version 1.81

# Command line: GENECONV .\G3-449N_G4-136N.clw /r -Include_monosites

# /w123 /lp

#

# .\G3-449N_G4-136N.clw: 2 sequences:

# CLUSTAL W (1.81) multiple sequence alignment

#

# Options and features: 2 DNA sequences, SILENT SITES only,

# 814 aligned bases, 116 polymorphisms, CLUSTAL format,

# mismatches in fragments not allowed, no groups defined,

# maxSimPairPval=0.05, maxSimGlobPval=0.05, no maxKAPairPval limits,

# no maxKAGlobPval limits, simPvals based on 10,000 permutations,

# global P-values based on BLAST-like global scores,

# fragment limits: minlength=1, minnpolys=2, minscore=2,

# pairwise max lists=2000, starting seed=123,

# sites with indels are skipped,

# USING MONOMORPHIC AS WELL AS POLYMORPHIC CODON POSITIONS,

# no match (replacement) char in source file.

# Sequence names: G3-449N G4-136N

#

# No mismatches in fragments (`gscale'=0)

# Nucleotide codes in data: T(361) C(326) A(246) G(333) Other(362)

# CG/TCAG ratio: 52.05%

# Non-nucleotide characters in data: -(2DH)(n=362)

# 362 sites with missing data or unrecognized characters.

# No sites with all gap or unrecognized characters.

# 55 polymorphic and 397 monomorphic sites with no indels.

# 128 codon positions with indels or missing data.

# 27 amino-acid polymorphic codon positions with no indels.

# 116 silent codon polymorphisms to be permuted.

# CODON POSITIONS with unknown or missing data are excluded.

# The silent sites at 116 amino-acid monomorphic codon positions are

# 0-degen (6) 2-degen (35) 3-degen (2) 4-degen (57) irreg (16)

# The silent polymorphic sites from these codon positions are

# 0-degen (0) 2-degen (35) 3-degen (2) 4-degen (57) irreg (22)

# Codon usage for 3-fold degenerate codons: (Ile) ATT(3) ATC(2) ATA(0)

#

# The starting random number seed is 123.

# Simulated P-values are based on 10,000 permutations.

# Maximum BLAST-like scores:

# Inner Max Sim S.D.s above S.D. of

# frags Score P-value sim. mean sims

# SCORE 1.166 0.1505 1.08 0.6616

# OuterSeq

# frags -1.884 1.0000 -0.67 1.4305

#

# Global lists: no I and no O significant fragments

# Pairwise lists: no I and no O significant fragments

# where I means inner pair, O is outer seq, A is outer pair, and

# G means outer group (fragments).

# Global P-values are based on BLAST-like global scores.

#

# Fragment offsets and lengths are in nucleotides.

# Bases other than `TCAGU' are treated as indels

# for the purpose of generating polymorphisms.

#

#

# Global inner fragments (116 polymorphisms, 814 aligned bases):

# (Inner fragments are runs of matching sites.)

# (Sim and BC KA P-values are corrected for multiple comparisons.)

# (Fragments are listed only if Sim P-value <= 0.05.)

# See earlier in the file for the full restrictions on fragments.

#

#

# No inner fragments listed.

#

#

# Global outer-sequence fragments (116 polymorphisms, 814 aligned bases):

# (Outer-sequence fragments are runs of unique sites.)

# (Sim and BC KA P-values are corrected for multiple comparisons.)

# (Fragments are listed only if Sim P-value <= 0.05.)

# See earlier in the file for the full restrictions on fragments.

#

#

# No outer-sequence fragments listed.

#

#

# Pairwise inner fragments (116 polymorphisms, 814 aligned bases):

# (Inner fragments are runs of matching sites.)

# (P-values are not corrected for multiple comparisons.)

# (Fragments are listed only if Sim P-value <= 0.05.)

# See earlier in the file for the full restrictions on fragments.

#

# No inner fragments listed.

#

#

# Pairwise outer-sequence fragments (116 polymorphisms, 814 aligned bases):

# (Outer-sequence fragments are runs of unique sites.)

# (P-values are not corrected for multiple comparisons.)

# (Fragments are listed only if Sim P-value <= 0.05.)

# See earlier in the file for the full restrictions on fragments.

#

# No outer-sequence fragments listed.

#

Group 3

CLUSTAL W (1.81) multiple sequence alignment

Lu2-51734 ATGATGGCTTCCTCTCTGGCTGTGTCTGCCGCTAGCCTAGTTGTTACCGCTGCTGGTACT

Lu13-23576 ATG-----------TTCGGCAAG-------GCTCATCAGGATGGTTCCGTGTTTGTCGGT

*** * *** *** * * ** * *** ** *

Lu2-51734 AATGTCTTCCCATCCAGGAA--CACACCCAACTTTTTCCTTGCTAATAACAAATCCACAT

Lu13-23576 CAGAGTTCATTGTTCGGGAAGGCTCATCAGGATGGTTCCGTGTTTGT-------------

* * * * **** * ** * * **** ** * *

Lu2-51734 CGCCACTGAAACCCATCATCTCATGCCATCCCGGTGGTGGTTCCTTGCGAAGCAAGGCTC

Lu13-23576 -------------------------------CGGCCAGAGTTCATTGTTCGGCAAGGCTC

*** **** *** *********

Lu2-51734 ATCAGGATGGTTCTTTGTTTGGCGGACAGGGTGCCATGTTCGGCAAGGCTCATCAGGATG

Lu13-23576 ATCAGGATGGTTCCTTGTTCGGCGGCCAGGGTGCCATGTTCGGCAAGGCTCATCAGGATG

************* ***** ***** **********************************

Lu2-51734 GTTCCTTGTTCGGCGGACAGGGTGCCATGTTCGGCAAGGCTCATCAGGATGGTTCCTTGT

Lu13-23576 GTTCCGTGTTTGTCGGCCAGAGTTCATTGTTCGGCAAGGCTCATCAGGATGGTTCCTTGT

***** **** * *** *** ** * *********************************

Lu2-51734 TTGGCGGCCAGGGTGCCATGTTCGGCAAGGCTCATCAGGATGGTTCCTTGTTCGGCGGCC

Lu13-23576 TCGGCGGCCAGGGTGCCATGTTCGGCAAGGCTCATCAGGACGGTTCCGTGTTCGTCGGCC

* ************************************** ****** ****** *****

Lu2-51734 AGGGTGCCATGTTCGGCAAGGCTCATCAGGATGTCCATCAGGATGGTTCCTTGTTCGGCG

Lu13-23576 AGGGTTCATTGTTCGGCAAGGCT------------CATCAGGATGGTTCCTTGTTCGGCG

***** * ************** *************************

Lu2-51734 GCCAGGGTGCCATGTTCGACAAGGCTCATCAGGATGGTTCCTTGTTCGGCGGCCAGGGTG

Lu13-23576 GCCAGGGTGCCATGTTCGGCAAGGCTCATCAGGATGGTTCCGTGTTTGTCGGCCAGAGTT

****************** ********************** **** * ******* **

Lu2-51734 CCATGTTCGGCAAGGCTCATCAGGATG--TCCAT----------CAGGATGTC-------

Lu13-23576 CATTGTTGGGCAAGGCTCATCAAGATGGTTCCTTGTTCGGCGGCCAGGGTGCCATGTTCG

* **** ************** **** *** * **** ** *

Lu2-51734 --------CATCAGGATGGTTCCTTGTTCGGCGGCCAGGGTGCCATGTGCGGCAAGGCTC

Lu13-23576 GCAAGGCTCATCAGGATGGTTCCGTGTTTGTCGGCCAGAGTTCATTGTTTGGCAAGGCTC

*************** **** * ******* ** * *** **********

Lu2-51734 ATCAGGATGGTTCCTTGTTCGGCGGCCAGGGTGCCATGTTCGGCAAGGCTCATCAGGATG

Lu13-23576 ATCAAGATGGTTCCTTGTTCGGCGACCAGGGTGCCATGTTCTGCAAGGCTCATCAGGATG

**** ******************* **************** ******************

Lu2-51734 GTTCCTTGTTCGGCGGCCAAGGTGCCATGTTCGGCAAGGCTCATCAGGATGGTTCCTTGT

Lu13-23576 GTTCCGTGTTTGTCGGCCAGAGTTCATTGTTTGGCAAGACTCATCAAGATGGTTCCTTGT

***** **** * ****** ** * **** ****** ******* *************

Lu2-51734 TCGGCGGCCAAGGTGCCATGTTCGGCAAGGCTCATCAGGATGGTGCTTTGTTCGGCGGCC

Lu13-23576 TCGGCGGCCAGGGTGCCATGTTCGGCAAGGCTCATCAGGACGGTTCCGTGTTCGTCGGTC

********** ***************************** *** * ****** *** *

Lu2-51734 AAGGTGCCATGTTCGGTAAGGTTCATCAGGATGGTTCCTTGTTTGGCGGCCAGGGTGCCA

Lu13-23576 AGAGTTCATTGTTTGGCAAGGCTCATCAAGATGGTTCCTTGTTCGGCGGCCAGGATGCCA

* ** * **** ** **** ****** ************** ********** *****

Lu2-51734 TGTTCGGCAAGGCTCATCAG------------------------GATGGTTCCTTGTTTG

Lu13-23576 GGTTCGGCAAGGCTCATCAAGATGTCCATCAGGATGGTCATCATGATGGTTCCTTGTTCG

****************** ************** *

Lu2-51734 GCGGCCAGGGTGCCATGTTCGGCAAGGCT------------CATCAGGATGGTTCCTTGT

Lu13-23576 GCGGCCAAGGTGCCATGTTCGGCAAGGCTCATCAGGATGCCCATCAGGATGGTACCTTGT

******* ********************* ************ ******

Lu2-51734 TCGG------------------------TGGCCAAGGTGCCATGTTCGGCAAAGGTCAAG

Lu13-23576 TTGGAAAGGCTCGTGTCGAAGGCGATTTAGGCGATGGTTCCTTGTTCGGCAAAGGTCAAG

* ** *** * *** ** ******************

Lu2-51734 ATGTCAAGGGGGGTGACAACTTTGTTGCAGCACCAATCCTCAAAGAATGA

Lu13-23576 ATGTCAAGAGGGGTGACAACTTTGTTGCAGCACCAATTCTTAAAGAATGA

******** **************************** ** *********

# Output of GENECONV for sequence file .\Lu2-51734_Lu13-23576.clw

# GENECONV version 1.81

# Command line: GENECONV .\Lu2-51734_Lu13-23576.clw /r -Include_monosites

# /w123 /lp

#

# .\Lu2-51734_Lu13-23576.clw: 2 sequences:

# CLUSTAL W (1.81) multiple sequence alignment

#

# Options and features: 2 DNA sequences, SILENT SITES only,

# 1070 aligned bases, 217 polymorphisms, CLUSTAL format,

# mismatches in fragments not allowed, no groups defined,

# maxSimPairPval=0.05, maxSimGlobPval=0.05, no maxKAPairPval limits,

# no maxKAGlobPval limits, simPvals based on 10,000 permutations,

# global P-values based on BLAST-like global scores,

# fragment limits: minlength=1, minnpolys=2, minscore=2,

# pairwise max lists=2000, starting seed=123,

# sites with indels are skipped,

# USING MONOMORPHIC AS WELL AS POLYMORPHIC CODON POSITIONS,

# no match (replacement) char in source file.

# Sequence names: Lu2-51734 Lu13-23576

#

# No mismatches in fragments (`gscale'=0)

# Nucleotide codes in data: T(540) C(469) A(344) G(624) Other(163)

# CG/TCAG ratio: 55.29%

# Non-nucleotide characters in data: -(2DH)(n=163)

# 163 sites with missing data or unrecognized characters.

# No sites with all gap or unrecognized characters.

# 141 polymorphic and 766 monomorphic sites with no indels.

# 64 codon positions with indels or missing data.

# 75 amino-acid polymorphic codon positions with no indels.

# 217 silent codon polymorphisms to be permuted.

# CODON POSITIONS with unknown or missing data are excluded.

# The silent sites at 217 amino-acid monomorphic codon positions are

# 0-degen (15) 2-degen (36) 3-degen (15) 4-degen (103) irreg (48)

# The silent polymorphic sites from these codon positions are

# 0-degen (0) 2-degen (36) 3-degen (15) 4-degen (103) irreg (63)

# Codon usage for 3-fold degenerate codons: (Ile) ATT(0) ATC(30) ATA(0)

#

# The starting random number seed is 123.

# Simulated P-values are based on 10,000 permutations.

# Maximum BLAST-like scores:

# Inner Max Sim S.D.s above S.D. of

# frags Score P-value sim. mean sims

# SCORE 0.698 0.3208 0.34 0.9445

# OuterSeq

# frags 1.113 0.2583 1.30 1.2094

#

# Global lists: no I and no O significant fragments

# Pairwise lists: no I and no O significant fragments

# where I means inner pair, O is outer seq, A is outer pair, and

# G means outer group (fragments).

# Global P-values are based on BLAST-like global scores.

#

# Fragment offsets and lengths are in nucleotides.

# Bases other than `TCAGU' are treated as indels

# for the purpose of generating polymorphisms.

#

#

# Global inner fragments (217 polymorphisms, 1070 aligned bases):

# (Inner fragments are runs of matching sites.)

# (Sim and BC KA P-values are corrected for multiple comparisons.)

# (Fragments are listed only if Sim P-value <= 0.05.)

# See earlier in the file for the full restrictions on fragments.

#

#

# No inner fragments listed.

#

#

# Global outer-sequence fragments (217 polymorphisms, 1070 aligned bases):

# (Outer-sequence fragments are runs of unique sites.)

# (Sim and BC KA P-values are corrected for multiple comparisons.)

# (Fragments are listed only if Sim P-value <= 0.05.)

# See earlier in the file for the full restrictions on fragments.

#

#

# No outer-sequence fragments listed.

#

#

# Pairwise inner fragments (217 polymorphisms, 1070 aligned bases):

# (Inner fragments are runs of matching sites.)

# (P-values are not corrected for multiple comparisons.)

# (Fragments are listed only if Sim P-value <= 0.05.)

# See earlier in the file for the full restrictions on fragments.

#

# No inner fragments listed.

#

#

# Pairwise outer-sequence fragments (217 polymorphisms, 1070 aligned bases):

# (Outer-sequence fragments are runs of unique sites.)

# (P-values are not corrected for multiple comparisons.)

# (Fragments are listed only if Sim P-value <= 0.05.)

# See earlier in the file for the full restrictions on fragments.

#

# No outer-sequence fragments listed.

#

Group 4

CLUSTAL W (1.81) multiple sequence alignment

Lu5-45630 ATGGCGCCATCTCATTTGCGTTTCTTACTACTTGCACTGGTTCTGGTATGCTCCTCCTCA

Lu8-3470 ------------------------------------------------------------

Lu5-45630 ATTTATCATTTCTGCATATGCCAGCAAGTTGGATACTCTATACTTATATGATCGTCTTTG

Lu8-3470 -----------------------------------------------ATGATC----TTG

****** ***

Lu5-45630 ATGATGTATATGCAGTTGGGAGCTGGTTGTTGCAGTGTCAATGGAATTAGGAAAGACCCA

Lu8-3470 ATGTTGC---TGCAGTTGGGAGCTGAT------AGTGTCAATGCA---AGAAAAGACTCA

*** ** *************** * ********** * ** ****** **

Lu5-45630 ATAGGAGATTACTGGAGATCTGTGATGAAAGATGAGCCTATGCCTAAAGCAATCCAATCC

Lu8-3470 ATAGGGGGTTACTGGAAATCTGTGATGAAAGATCAGCTTATGCCCAAAGCAATCGAATCC

***** * ******** **************** *** ****** ********* *****

Lu5-45630 CTACTACTTCCTGCTGCAAATTCTGATCATCAACACCCTGCTGTCCATTATGATACTGAT

Lu8-3470 CTACTG---CCTGCTGCAAACTCTGAT------CACGCCGATGTACGTTGTGATACTAAT

***** *********** ****** *** * * *** * ** ******* **

Lu5-45630 CACAACTTCAAAAACCCT-----------------------------------GATC-AG

Lu8-3470 CAGGTCTTCCAGTACCACAAGGATGCCGTGCCATCCAACGAATTTTCCAAGGAGATCGAG

** **** * *** **** **

Lu5-45630 TCACAACCTGATGAACACATCTTCGAGTACTATAGCAAAGATGATGCCCTGCCATCCAAC

Lu8-3470 TCGCAACCTAAC---CAAGTATTCCAATACCACA------AGGATATCGTGCCATCTAAT

** ****** * ** * *** * *** * * * *** * ******* **

Lu5-45630 AAATTCGCAAAGGGGATTGGGTTGAAACCTGACGAAGCCCTCTTGTTCTATAGCAAAGAT

Lu8-3470 GAATTTGCGGAAGGGACCGAGTCGCAACCTAATCAAGTCTTCCAGTACCAC------GAG

**** ** * **** * ** * ***** * *** * ** ** * * **

Lu5-45630 GATGCCCCTCCATCCAACAAATTCGCAAAGGGGATTGGGTTGAAACCTGACGAAGCCCTC

Lu8-3470 GATGCCGTGCCATCTAATGAATTTGCCAAGGAGATCGAGTTGCAGCCTAACCAAGTCTTC

****** ***** ** **** ** **** *** * **** * *** ** *** * **

Lu5-45630 TTGTTCTATAGCAAAGATGATGCCCCTCCATCCAACAAATTCGCAAAGGGGATTGGGTTG

Lu8-3470 CAGTACCACA------AGGATGCCGTGCCATCTAATGAATTTGCCAAGGAAATCGAGTCG

** * * * * ****** ***** ** **** ** **** ** * ** *

Lu5-45630 AAACCTGACGAAGCCCTCTTGTTCTATAGCAAAGATGATGCCCCTCCATCCAACAAATTC

Lu8-3470 CAGCCTAATCAAGTCTTCCAGTACCACA------AGGATGCCGTGCCATCTAATGAATTT

* *** * *** * ** ** * * * * ****** ***** ** ****

Lu5-45630 GCAAAGGGGATTGGGTCGAAACCTGACGAAGCCCTCT-----------------------

Lu8-3470 GCCAAGGAAATCGAGTCGCAGCCTAATCAAGTCTTCCAGTACCACAAGGATGTCGTGACA

** **** ** * **** * *** * *** * **

Lu5-45630 -------------------------------------------TCTTCTACAGCAAAGAT

Lu8-3470 TCTAATGAATTTTCCAAGGGGATGGAATTGCAGCCTAATCAAGTCTTCCAGTACCACAAG

***** * * * *

Lu5-45630 GATGCCCCGCCATCCAACAAATTCGCAAAGGGGATTGGATCGAAACCTGACGAAGCCCTC

Lu8-3470 GATATCGTGCCATCCAACGAATTTGCCAAGGGAATCGAATCGCAGCCTAATCAAGTCTTC

*** * ********** **** ** ***** ** * **** * *** * *** * **

Lu5-45630 T-----------------------------------------------------------

Lu8-3470 CAGTACCACAAGGATGTCGTGCCATCCAACGAATTCCCCAAGGAGATCGAGACACGGCCT

Lu5-45630 -------TCTTCTACAGCAAAGATGATGCCCCGCCATCCAACAAATTCGCAAAGGGGATT

Lu8-3470 AATCAAGTCTTCCAGTACCACAAGGATGTCGTGCCGTCAAATGAATTTTCCAAAGGGATC

***** * * * * **** * *** ** ** **** * ** *****

Lu5-45630 GGATCGAAACCTGACGAAGCCCTCTTCTTCTATAGCAAAGAT------------------

Lu8-3470 GGGTCACAACCTAATCAAG---TCTTCCAGTACAACAAGGATGTCGTGCCATCAAATGAA

** ** ***** * *** ***** ** * *** ***

Lu5-45630 ---------------------------------------------------GATGCCCCG

Lu8-3470 GGTTTCAAGGAGATCGAGTCGCAACCTAATCAAGTATTCCAATACCACAAAGATGCCGTG

****** *

Lu5-45630 CCATCCAACAAATTCGCAAAGGGGATTGGATCGCGACCTGATGAAGCCCTCTTGTTCTAT

Lu8-3470 CCATCTAATGAATTTGCGGTTGGGACCGAGACACAACCTAATCAAGTCTTCCAGTACCAC

***** ** **** ** **** * * * **** ** *** * ** ** * *

Lu5-45630 AG---------------------------CAAATGA------------

Lu8-3470 AAGGATGTCGTGCCATCCAACGAATTTGCCAAGGGAATCGAGTCTTAG

* *** **

# Output of GENECONV for sequence file .\Lu5-45630_Lu8-3470.clw

# GENECONV version 1.81

# Command line: GENECONV .\Lu5-45630_Lu8-3470.clw /r -Include_monosites

# /w123 /lp

#

# .\Lu5-45630_Lu8-3470.clw: 2 sequences:

# CLUSTAL W (1.81) multiple sequence alignment

#

# Options and features: 2 DNA sequences, SILENT SITES only,

# 1188 aligned bases, 125 polymorphisms, CLUSTAL format,

# mismatches in fragments not allowed, no groups defined,

# maxSimPairPval=0.05, maxSimGlobPval=0.05, no maxKAPairPval limits,

# no maxKAGlobPval limits, simPvals based on 10,000 permutations,

# global P-values based on BLAST-like global scores,

# fragment limits: minlength=1, minnpolys=2, minscore=2,

# pairwise max lists=2000, starting seed=123,

# sites with indels are skipped,

# USING MONOMORPHIC AS WELL AS POLYMORPHIC CODON POSITIONS,

# no match (replacement) char in source file.

# Sequence names: Lu5-45630 Lu8-3470

#

# No mismatches in fragments (`gscale'=0)

# Nucleotide codes in data: T(465) C(486) A(578) G(409) Other(438)

# CG/TCAG ratio: 46.18%

# Non-nucleotide characters in data: -(2DH)(n=438)

# 438 sites with missing data or unrecognized characters.

# No sites with all gap or unrecognized characters.

# 223 polymorphic and 527 monomorphic sites with no indels.

# 156 codon positions with indels or missing data.

# 115 amino-acid polymorphic codon positions with no indels.

# 125 silent codon polymorphisms to be permuted.

# CODON POSITIONS with unknown or missing data are excluded.

# The silent sites at 125 amino-acid monomorphic codon positions are

# 0-degen (4) 2-degen (50) 3-degen (8) 4-degen (56) irreg (7)

# The silent polymorphic sites from these codon positions are

# 0-degen (0) 2-degen (50) 3-degen (8) 4-degen (56) irreg (11)

# Codon usage for 3-fold degenerate codons: (Ile) ATT(7) ATC(12) ATA(2)

#

# The starting random number seed is 123.

# Simulated P-values are based on 10,000 permutations.

# Maximum BLAST-like scores:

# Inner Max Sim S.D.s above S.D. of

# frags Score P-value sim. mean sims

# SCORE 0.656 0.3615 0.37 1.0566

# OuterSeq

# frags 0.726 0.3817 0.54 1.1782

#

# Global lists: no I and no O significant fragments

# Pairwise lists: no I and no O significant fragments

# where I means inner pair, O is outer seq, A is outer pair, and

# G means outer group (fragments).

# Global P-values are based on BLAST-like global scores.

#

# Fragment offsets and lengths are in nucleotides.

# Bases other than `TCAGU' are treated as indels

# for the purpose of generating polymorphisms.

#

#

# Global inner fragments (125 polymorphisms, 1188 aligned bases):

# (Inner fragments are runs of matching sites.)

# (Sim and BC KA P-values are corrected for multiple comparisons.)

# (Fragments are listed only if Sim P-value <= 0.05.)

# See earlier in the file for the full restrictions on fragments.

#

#

# No inner fragments listed.

#

#

# Global outer-sequence fragments (125 polymorphisms, 1188 aligned bases):

# (Outer-sequence fragments are runs of unique sites.)

# (Sim and BC KA P-values are corrected for multiple comparisons.)

# (Fragments are listed only if Sim P-value <= 0.05.)

# See earlier in the file for the full restrictions on fragments.

#

#

# No outer-sequence fragments listed.

#

#

# Pairwise inner fragments (125 polymorphisms, 1188 aligned bases):

# (Inner fragments are runs of matching sites.)

# (P-values are not corrected for multiple comparisons.)

# (Fragments are listed only if Sim P-value <= 0.05.)

# See earlier in the file for the full restrictions on fragments.

#

# No inner fragments listed.

#

#

# Pairwise outer-sequence fragments (125 polymorphisms, 1188 aligned bases):

# (Outer-sequence fragments are runs of unique sites.)

# (P-values are not corrected for multiple comparisons.)

# (Fragments are listed only if Sim P-value <= 0.05.)

# See earlier in the file for the full restrictions on fragments.

#

# No outer-sequence fragments listed.

#

Group 5

CLUSTAL W (1.81) multiple sequence alignment

Lu5-46938 ATGTCTATGAAATCAGGCAGGAAGGGGGATAGCGCCGCCCTCTTGACCATCGCTTCAAAG

Lu8-2811 ATGTCTATGAAATCAGGCAGGAAGGGGGATAGCGCCGCCCTCTTGACCTTCGCTTCAAAG

************************************************ ***********

Lu5-46938 GCCAGGGATGCCGGCCTCATTCCTGTTCCGATGTTCGGTCTGGCAGAAACCAGCAATGCT

Lu8-2811 G---GGGATGCCACCCTCATTCCTGTTCCGATGTTCGGCAAGGCGGAAATGATGGA---T

* ******** ************************ *** **** * * *

Lu5-46938 CCAGAGGGGGATGCCGGCATCATTGATCCTATCTTACTGATATTTGGTAAGCTCGAAATG

Lu8-2811 CGAAAGGGGGATGCCGGCATCATTGATCCTACCTTACCGATATTTGGTAAGGTGGAAATG

* * *************************** ***** ************* * ******

Lu5-46938 ATGGCTCGAAATGGGGATGCCGGCTTCGTTCCCAACTTTTCGGTATTTGGCAAGGAGGAT

Lu8-2811 ATGGCTCGAAAT------------------------------------------------

************

Lu5-46938 GCCGCCCTATTTATTCCTTCTTTCCCGATATTTGGCAAGGAGGAGAGGATGGCTCCAAAG

Lu8-2811 ------------------------------------AAGGTCGAAATGATGGCTCCAAAG

**** ** * *************

Lu5-46938 GGGGATGCCGGCTTCGATATTTTCTTCCCGTTCTTCGGTAAGGCGGACATAATGATGCCA

Lu8-2811 GGTGATGCCGGCTTCGATATTTTCTTCCCGTTCTTCGGTAAGCAGGCAGAAAGGATGCCA

** *************************************** ** ** *******

Lu5-46938 CCAAAGGAGGATGCCGGCTTCGACATTTTCTTCCCGTTCTTCGGTAAGCAGGCAGAAATG

Lu8-2811 CCAAAGGAGGATGCTGGCTTCGACAATTTCTTCCCGTTCTTCGGTAAGCAGGCAGAAATG

************** ********** **********************************

Lu5-46938 ATGACTCCAAATGGGGATGACGGTCCGAGTGCATGA------------------------

Lu8-2811 ATGACTCCAAA-GGGGGTGACGCTCCGAGTGCATGCACAAGATCACGACATAAATGTTCT

*********** **** ***** ************

Lu5-46938 -------------------------------------------------------

Lu8-2811 CTCCTTCCATAGGCATATATGCTTAATTAATTATAAAAATCTGATGAGTTCATAG

# Output of GENECONV for sequence file .\Lu5-46938_Lu8-2811.clw

# GENECONV version 1.81

# Command line: GENECONV .\Lu5-46938_Lu8-2811.clw /r -Include_monosites

# /w123 /lp

#

# .\Lu5-46938_Lu8-2811.clw: 2 sequences:

# CLUSTAL W (1.81) multiple sequence alignment

#

# Options and features: 2 DNA sequences, SILENT SITES only,

# 535 aligned bases, 99 polymorphisms, CLUSTAL format,

# mismatches in fragments not allowed, no groups defined,

# maxSimPairPval=0.05, maxSimGlobPval=0.05, no maxKAPairPval limits,

# no maxKAGlobPval limits, simPvals based on 10,000 permutations,

# global P-values based on BLAST-like global scores,

# fragment limits: minlength=1, minnpolys=2, minscore=2,

# pairwise max lists=2000, starting seed=123,

# sites with indels are skipped,

# USING MONOMORPHIC AS WELL AS POLYMORPHIC CODON POSITIONS,

# no match (replacement) char in source file.

# Sequence names: Lu5-46938 Lu8-2811

#

# No mismatches in fragments (`gscale'=0)

# Nucleotide codes in data: T(229) C(204) A(221) G(246) Other(170)

# CG/TCAG ratio: 50.00%

# Non-nucleotide characters in data: -(2DH)(n=170)

# 170 sites with missing data or unrecognized characters.

# No sites with all gap or unrecognized characters.

# 35 polymorphic and 330 monomorphic sites with no indels.

# 59 codon positions with indels or missing data.

# 20 amino-acid polymorphic codon positions with no indels.

# 99 silent codon polymorphisms to be permuted.

# CODON POSITIONS with unknown or missing data are excluded.

# The silent sites at 99 amino-acid monomorphic codon positions are

# 0-degen (9) 2-degen (39) 3-degen (5) 4-degen (40) irreg (6)

# The silent polymorphic sites from these codon positions are

# 0-degen (0) 2-degen (39) 3-degen (5) 4-degen (40) irreg (15)

# Codon usage for 3-fold degenerate codons: (Ile) ATT(7) ATC(4) ATA(3)

#

# The starting random number seed is 123.

# Simulated P-values are based on 10,000 permutations.

# Maximum BLAST-like scores:

# Inner Max Sim S.D.s above S.D. of

# frags Score P-value sim. mean sims

# SCORE -0.158 0.8982 -1.07 0.5695

# OuterSeq

# frags 1.428 0.1891 1.97 1.2226

#

# Global lists: no I and no O significant fragments

# Pairwise lists: no I and no O significant fragments

# where I means inner pair, O is outer seq, A is outer pair, and

# G means outer group (fragments).

# Global P-values are based on BLAST-like global scores.

#

# Fragment offsets and lengths are in nucleotides.

# Bases other than `TCAGU' are treated as indels

# for the purpose of generating polymorphisms.

#

#

# Global inner fragments (99 polymorphisms, 535 aligned bases):

# (Inner fragments are runs of matching sites.)

# (Sim and BC KA P-values are corrected for multiple comparisons.)

# (Fragments are listed only if Sim P-value <= 0.05.)

# See earlier in the file for the full restrictions on fragments.

#

#

# No inner fragments listed.

#

#

# Global outer-sequence fragments (99 polymorphisms, 535 aligned bases):

# (Outer-sequence fragments are runs of unique sites.)

# (Sim and BC KA P-values are corrected for multiple comparisons.)

# (Fragments are listed only if Sim P-value <= 0.05.)

# See earlier in the file for the full restrictions on fragments.

#

#

# No outer-sequence fragments listed.

#

#

# Pairwise inner fragments (99 polymorphisms, 535 aligned bases):

# (Inner fragments are runs of matching sites.)

# (P-values are not corrected for multiple comparisons.)

# (Fragments are listed only if Sim P-value <= 0.05.)

# See earlier in the file for the full restrictions on fragments.

#

# No inner fragments listed.

#

#

# Pairwise outer-sequence fragments (99 polymorphisms, 535 aligned bases):

# (Outer-sequence fragments are runs of unique sites.)

# (P-values are not corrected for multiple comparisons.)

# (Fragments are listed only if Sim P-value <= 0.05.)

# See earlier in the file for the full restrictions on fragments.

#

# No outer-sequence fragments listed.

#

Group 6

CLUSTAL W (1.81) multiple sequence alignment

Lu14-5765 ATGGAGGTCTCTTACGAGAT------------TACTT-----------------------

Lu12-11761 ATGGCTTCCTCTTTCGCCATGCCCGCCGCTGCTACTTTCTTGCCGGTGGCATCATCACCA

Lu6-41637 ------------------ATGCCCGCTGCTGCTACTTTCTCGTCGGTGGCATCATCGCCA

Lu12-11698 ATGGCTTCCTCTTTCGCCATGCCCGCCGCTGCTACTTTCTCGTCGGTGGCATCATCGCCA

** *****

Lu14-5765 --------CGCCAAGCAG-----------------------------------CAGGACG

Lu12-11761 CCGCAACACTCCAAGAAGAATATGACGATGACGCTGCTTACTAGCCCCAAAACTAGCTCA

Lu6-41637 CCGCAACACTCTAAAAAGAACATGACAACGATGCTTACTGCTAGCCTCAAAACTAGCTCA

Lu12-11698 CCGCAACACACCAAAAAGAATATGACGACGGTGCTTACTGCTAGCCCCAAAACTAGGTCA

* * ** ** ** *

Lu14-5765 AAGGT--------------CTTTTGTACTTTTC--CAAATAGCAGGACGGTGATTTTGTG

Lu12-11761 AAGGTGTCGGTTTCTGCTGTTGTTGTCTCCGTCAACAAGCAACAGGACGGAGGTATTCGG

Lu6-41637 AAGGTGTCGGTTTCTGCTGCTGTTGTCTCCTTCAACAAGCAGCAGGATGGAGGTATTCGG

Lu12-11698 AAGGTGTCGGTTTCTGCTGCTGTTGTCTCCTTCAACAAGCAGCAGGACGGAGGTATTCCA

***** * **** ** *** * ***** ** * * **

Lu14-5765 CATCCCTACTTCGTCAAGGAACAAGACGGAGGTCTATTAAGAGACTACTTCGCCAAGGAA

Lu12-11761 CTCCCCTACTTTGCCAAGCAGAAGGACGGAGGTGT-------------------------

Lu6-41637 CGCACCTACTTCGCCAAGCAGCAGGATGGAGGTCTCAGACAAGGCTACTTCGCCAAGGAG

Lu12-11698 CGCACCTACTTCGCCAAGCAGACTGACGGAGGTCTTTTGAAAGACTACTTCGCCAAGAAA

* ******* * **** * ** ****** *

Lu14-5765 CAGAACACAGGTCTTTTACGAGACTACTTCGCCAAGGAGCAGGACAGAGGTCTCTTGCGA

Lu12-11761 ------------------------------------------------------TTGGGA

Lu6-41637 AAGGACGGAGGTCTTAGACAAGGCTACTTCGCCGAA------GACGGAGGTCTCTTGCGA

Lu12-11698 CATGACGGAGGTATTCGACGCACCTACTTTGCCAAGCAGTATGATGGAGGTCTTTTGAAA

*** *

Lu14-5765 GACTACTTTGATAAGTAGCAGGACAGAGGTCTTTTGCGAGACTACTTTGCCAAACAGTGG

Lu12-11761 CGCT--------------------------------------------------------

Lu6-41637 GACTACTTTGCCAAGGAACAGGACGGAGGTCTCAGACAAGGCTACTTCGCCAAGCAACAA

Lu12-11698 GACTACTTCGCCAAGGAATATGACGGAGGTGAATGGGCTGCCTTCTTCGGCAAACAGCAT

**

Lu14-5765 GACAGTGGTTTTGTGTGCCCCTCCTTCGCCAAGGAACAGGACACATGTCTTTTACGAGAC

Lu12-11761 ---------------------TTCTTCGGCAAGCAACAAGACGGAGGTGAATGGGCTGCC

Lu6-41637 GACGAAGGTCTCAGACAAGGCTACTTTGCCAAGCAACAAGACGGAGGCCTCAGACAAGGC

Lu12-11698 GACGGGGGTATTCGACGCACCTACTTCACCAAGGAACATGACGGAGGTGAATGGGCTGCC

* *** **** **** *** * * * *

Lu14-5765 TACTTCGCCAAGGAGCAAGACAGAGGTCTCTTGCGAGACCACTTCGATAAGTAGCAGGAC

Lu12-11761 TTCTTCGGCAAGCAGGAAGACGGAGGTGAATGGGCTGCCTTCTTCGGCAAACAGCAGGAT

Lu6-41637 TACTTCGCCAAGGAAAAGGACGGAGGTCTCAGACAAGGCTACTTCGCCAAGGAAAAGGAC

Lu12-11698 TTCTTCGGCAAACAACATGACGGAGGTGAATGGGCTGCCTTCTTCGGCAAACAGCAGGAC

* ***** *** * * *** ***** * * ***** ** * ****

Lu14-5765 TGAGGTCTTTTGCGAGACTACTTCTCCAAACAGGAAGACGAAGGTCGTTTGCGAGATTAT

Lu12-11761 GGAGGTATCCGACGCACCTACTTCGCCAAGCAACGGGACGAAGGGCTTTTGGGAGATTAT

Lu6-41637 GGAGGTCTTAGACAAGGCTACTTCGCCGAG------GACGGAGGTCTCTTGCGAGACTAC

Lu12-11698 GAAGGTATCCGTCGCACCTACTTCGCCAAGCAACATGACAGAGGTCTTTTGCGAGATTAT

**** * * ******* ** * *** *** * *** **** **

Lu14-5765 TTCGTCAAGGAATAGGACGGAGGTCTCTTGAGAGACTACTTCGCCAAGGAACAGGATGG-

Lu12-11761 TTTGCTAAGCAAGAAGACGGAGGTCTTTTAGGAGACTACTTCGCCAAGCAATAGGACGG-

Lu6-41637 TTTGCCAAGGAACAGGACGGAGGTCTCAGACAAGGCTACTTCGCCAAAGAAAAGGACGGA

Lu12-11698 TTTGCCAAGCAAGAAGACGGAGGTCTTTTACGAGACTATTTCGCCAAGGAACAGGACGGA

** * *** ** * *********** ** *** ******** ** **** **

Lu14-5765 --------------------------------AGGTCTCTTGAGAGACTACTTCGCCAAG

Lu12-11761 --------------------------------AGGGCTTTTGGGAGACTATTTTGCTAAA

Lu6-41637 GGTCTTAGACAAGACTACTTCGCCGAGGACGGAGGTCTCTTGCGAGACTACTTTGCCAAG

Lu12-11698 GGTCTCTTACGGGACTACTTCGCGAAGGACGAAGGTCTCTTGCGAGACTACTTCGCGA--

*** ** *** ******* ** ** *

Lu14-5765 GAACAGGACGAAGGTCTCTTGCGAGACTACTTCACCAAGGAACATGACAGAGGTCTCTCG

Lu12-11761 CAAGAAGACGGAGGTCTTTTAGGAGACTACTTCGCCAAGCAACGGGACGGAGGGCTTTTG

Lu6-41637 GAACAGGACGGAGGTCTCAGACAAGGCTACTTCGCCAAGCAACAAGACGAAGGTCTCAGA

Lu12-11698 ----TGGACGGAGGACTTTTGCGAGACTACTTCGCCAAGGAACAGGATGGAGGTCTCTTG

**** *** ** ** ******* ***** *** ** *** **

Lu14-5765 CGAGACTACTTCGCCAAGGAACAGGACCGAGGTCTCTTACGAGACTACTTCGCCAAGCAG

Lu12-11761 GGAGACTATTTTGCTAAGCAAGAAGACGGAGGTCTTTTAGGAGACTACTTTGCCAAGCAA

Lu6-41637 CAAGGTTACTTCGCCAAGCAACAAGACGGAGGCCTCAGACAAGGCTACTTTGCTAAGGAA

Lu12-11698 CGAGACTACTTCGCCAAGGAACAGGACGGAGGTCTCAGACAAGGCTACTTTGCCAAGCAA

** ** ** ** *** ** * *** **** ** * ** ****** ** *** *

Lu14-5765 GAGGACGGAGGCCTTT-----GACTACTTTGCCAAACAGCAGGATGGTGGTTTTATGTGC

Lu12-11761 CGGGACGGAGGGCTTTTGGGAGACTATTTTGCTAAGCAAGAAGACAGAGGTATCCGACGC

Lu6-41637 AAGGACGGAGGTCTCAGACAAGGCTACTTCGCCAAGGAAAAGGACGGAGGTCTTAGACAA

Lu12-11698 CAGGACGGAGGTCTCAGACAAGGCTACTTTGGCAAGCAACAGGACGGAGGTCTCAGACAA

********* ** * *** ** * ** * * ** * *** *

Lu14-5765 CCCTACTTCGCTAAGCAGCAGGTCGGAGGTCTTTTGCGAGACTAGTTCGCCAAGGAACAG

Lu12-11761 ACCTACTTCGCCAAGCAACGAGACGGAGGGCTTTTGGGAGATTATTTTGCTAAGCAAGAA

Lu6-41637 GGCTACTTCGCCGAG------GACGGAGGTCTCTTGCGAGACTACTTTGCCAAGGAACAG

Lu12-11698 GGCTACTTCGGCAAGCAACAGGACGGAGGTCTCAGACAAGGCTACTTCGGCAAGCAACAT

******** ** * ****** ** ** ** ** * *** ** *

Lu14-5765 GACGAAGGTTTTTTAGGAGACTACTTCGCGAAGCAACAGGATGGGGGTCTTTTGCGAGAC

Lu12-11761 GACGGAGGTCTTTTAGGAGACTACTTCGCCAAGCAACGGGACGGAGGGCTTTTGGGAGAC

Lu6-41637 GACGGAGGTCTCAGACAAGGCTACTTCGCCAAGCAACAAGACGAAGGTCTCAGACAAGGC

Lu12-11698 GACAGAGGTCTCAGACAAGGCTACTTCGGCAAGGAACAGGACGGAGGTCTTAGACAAGGC

*** **** * * ** ******** *** *** ** * ** ** ** *

Lu14-5765 TACTTCGGCAAGCAGCAGGACATAGGTATTCGATGCACCTATTTTGCCAAACAACAGGAC

Lu12-11761 TATTTTGCTAAACAAGAAGACGGAGGTCTTTTAGGAGACTACTTCGCCAAGCAACGGAAC

Lu6-41637 TACTTCGCCAAGCAACAAGACAAAGGTCTCAGACAAGGCTACTTTGCTAAGGAAAAAGAC

Lu12-11698 TACTTCGGCAAGCAACAGGACGAAGGTCTCCGACAAGGCTACTTCGGCAAACAACAGGAC

** ** * ** ** * *** **** * * *** ** * ** ** **

Lu14-5765 GGAGGTCTCTTG-----TTACTTCGCCAAGGAA---------------------------

Lu12-11761 GGAGGGCTTTTGGGAGACTATTTTGCCAAGCAA---------------------------

Lu6-41637 GGAGGTCTCAGACAAGGCTACTTCGCCAAGGAAAAGGACGGAGGTCTTAGACAAGGTTAC

Lu12-11698 GGAGGTCTCAGACAAGACTACTTCAGCAAGCAACAGGACGGAGGTCTCAGACAAGGATAC

***** ** ** ** **** **

Lu14-5765 ------------------------------------------------------------

Lu12-11761 ------------------------------------------------------------

Lu6-41637 TTCGCCGAGGACGGAGGTCTCTTGCGAGACTACTTTGCTAAAGAACAGGACGGAGGTCTC

Lu12-11698 TTCAGCAAG---------------------------------CAACATGACGGAGGTCTC

Lu14-5765 ---------------------------------------------------------CAA

Lu12-11761 ---------------------------------------------------------GAA

Lu6-41637 AGACAAGGCTACTTCGCCAAGGAAAAGGACGGAAGTCTTAGACAAGGCTACTTCGTCGAG

Lu12-11698 AGACAAGGATACTTCAGCAAGCAA---------------------------------CAG

*

Lu14-5765 GATGGATGTCTTTTGCGAGACTACTTCAGCAAGCAACTGGACGGAGGTATTAGGCGCACA

Lu12-11761 GACGGAGGTCTTTTAGGAGACTACTTCGCCAAACAACGGGACGGAGGGCTTTTGAGAGAC

Lu6-41637 GACGGAGGTCTCTTGCGAGACTACTTTGCCAAGGAACAAGACGGAGGTCTCAGACAAGGC

Lu12-11698 GACGGAGGTCTCAGACAAGGCTACTTCGGCAAGCAACAGGACGGAGGTCTCAGACAAGGC

** *** **** ** ****** *** *** ******** *

Lu14-5765 TACTTTGCTAAAGAA---------------------------------CAGGATGGAGGT

Lu12-11761 TATTTTGCTAAGCAA---------------------------------GAAGACAGAGGT

Lu6-41637 TACTTCGCCAAAGAAAAGGACGGAGGTCTTAGACAAGGCTACTTCGCCGAGGACGGAGGT

Lu12-11698 TACTTCGGTAAGCAA---------------------------------CGGGACGGAGGT

** ** * ** ** ** *****

Lu14-5765 CTCTTGTGAGACTACTTCGTCAAGGAAC--------------------------------

Lu12-11761 CTTTTGGGAGACTACTTCGCCAAACAAC--------------------------------

Lu6-41637 CTCTTGCGAGACTACTTTGCCAAGCAACAGGACGGAGGTCTCAGACAAGGGTACTTCGCC

Lu12-11698 CTCAGACAAGGCTACTTCGGTAAGCAAC--------------------------------

** ** ****** * ** ***

Lu14-5765 -ATGATAAAGGTCTTTTGCGAGACTACTTCGTCAAGCAACAAGACAGAAGTATTCGACAC

Lu12-11761 -AGGACGGAGGGCTTTTGAGAGACTATTTTGCTAAGCAAGAAGACGGAGGT---------

Lu6-41637 GAGGACGGAGGTCTCTTGCGAGACTACTTCGCCAAGCAACAAGACGGAGGTCTCAGCCAA

Lu12-11698 -AAGACGGAGGTCTCAGGCAAGACTACTTCGCCAAG------GACGGAGGTCTCAGGCAA

* ** *** ** * ****** ** * *** *** ** **

Lu14-5765 ATATACTTTGTCAAGGAACAGGACATAAGTCTTTTGCGAGACTATTTTACCAAATAGCAG

Lu12-11761 ------------------------------CTTTTGGGAGACTATTTTGCCAAGGAACAT

Lu6-41637 GGCTACTTTGCCG------AGGATGAAGGCCTCTTGCGAGATTACTTCGCCAAGGAGCAG

Lu12-11698 GACTACTTCGCCA------AGGACGGAGGTCTCTTGGGAGACTACTTCGCCAAAAAACAA

** *** **** ** ** **** * **

Lu14-5765 GACGAAAATCTGTTGCAAAACTACTTCTCCAA-GAACAAGACGAAG--AGATGATAATAT

Lu12-11761 GACGGAGGTTTCGT---AGACTACTTCACTAAAAAACATGACGGAGGTGAATG-------

Lu6-41637 GATGAAAGTCTTTTGCGAGATCATTTCGCCAAGGAGCAAGATGGTG--GGACGATAGTAT

Lu12-11698 GACGGAGGTCTCTTGGGAGATTACTTCGCCAAGGAGCAGGACGGTG--GGATGATAGTAT

** * * * * * * * * *** * ** * ** ** * * * *

Lu14-5765 CTCCGCCAATGTTTGTGCTTGTCGGCAAGAATCAAGATGAAATACCCTCTAACTGACTTT

Lu12-11761 ----------GGCTGCCTTCTTCGGCAAGCAACAAGATGAAAAACCCTCCAAGTGA----

Lu6-41637 CTCCACCGATGTTTGTGCTTGTCGGCAAGAATCAAGATGAAATACCCTCTAACTGA----

Lu12-11698 CTTCGCCGATGTTCATGCTTGTCGGCAAGAATCAAGATGAAATACCCTCTAACTGA----

* * ******** * ********** ****** ** ***

Lu14-5765 TCTCCCGGTTGTGAAACATGCACGCAGGTAGCTCAAAATAAGGATATGTTTTACTATATA

Lu12-11761 ------------------------------------------------------------

Lu6-41637 ------------------------------------------------------------

Lu12-11698 ------------------------------------------------------------

Lu14-5765 TGTAGGACGTATACGTGCATGCTATGGGAATATCGGCGTATAAGCCCATTGAATAAAATT

Lu12-11761 ------------------------------------------------------------

Lu6-41637 ------------------------------------------------------------

Lu12-11698 ------------------------------------------------------------

Lu14-5765 CAAAAGCTTCTTTGATGATAAATAAAACCCGACTTTTGGGTTTCTTAATTATTCTAGTAA

Lu12-11761 ------------------------------------------------------------

Lu6-41637 ------------------------------------------------------------

Lu12-11698 ------------------------------------------------------------

Lu14-5765 AATTAATGATAATGTTCCTAATGATATATGTCTATGCGAAATGTATGAATGCATGTATGA

Lu12-11761 ------------------------------------------------------------

Lu6-41637 ------------------------------------------------------------

Lu12-11698 ------------------------------------------------------------

Lu14-5765 GCCGATCCACCGATTGAACCATTGTCGATCTCGAGGCCAAAAGCCCAAGATTGTTGATTT

Lu12-11761 ------------------------------------------------------------

Lu6-41637 ------------------------------------------------------------

Lu12-11698 ------------------------------------------------------------

Lu14-5765 TCTAATGTTATTTAGAAGCATTATTATTAAAAAGGTGTTCAATGTAAAATGACATAATTG

Lu12-11761 ------------------------------------------------------------

Lu6-41637 ------------------------------------------------------------

Lu12-11698 ------------------------------------------------------------

Lu14-5765 TATAGGCGATCACTTTCCGTGACTATCTTAATTATTAACAATTTAGAGATAGTAATGATC

Lu12-11761 ------------------------------------------------------------

Lu6-41637 ------------------------------------------------------------

Lu12-11698 ------------------------------------------------------------

Lu14-5765 ACATGTGCATAGAGGTAATTAAGCAAATTACTCCATGTTGTCTTGATTTGAACATAACAT

Lu12-11761 ------------------------------------------------------------

Lu6-41637 ------------------------------------------------------------

Lu12-11698 ------------------------------------------------------------

Lu14-5765 CTATATATGCTGATTGATCACATTTGGCAGAGAGATGCTGAAGAAGGACTTGCGAATGTG

Lu12-11761 ------------------------------------------------------------

Lu6-41637 ------------------------------------------------------------

Lu12-11698 ------------------------------------------------------------

Lu14-5765 GTCCCGTTGCTAATGAGCCAACGGGACGAAACTACAGTAGAAGCTATGGATGAAGAAATC

Lu12-11761 ------------------------------------------------------------

Lu6-41637 ------------------------------------------------------------

Lu12-11698 ------------------------------------------------------------

Lu14-5765 AAAGGTTTGATTGATGAGAAGACGAAGAAACTCCTAAGGTTGGTTTTGGACGAAAGGAGT

Lu12-11761 ------------------------------------------------------------

Lu6-41637 ------------------------------------------------------------

Lu12-11698 ------------------------------------------------------------

Lu14-5765 ACAGCAGTTCCTAGGGAATGCAGGGAGCTGTTCTGGAAAATGAACAGTTAA

Lu12-11761 ---------------------------------------------------

Lu6-41637 ---------------------------------------------------

Lu12-11698 ---------------------------------------------------

# Output of GENECONV for sequence file .\Lu6-41637_Lu12-11761_Lu12-11698_Lu14-5765.clw

# GENECONV version 1.81

# Command line: GENECONV .\Lu6-41637_Lu12-11761_Lu12-11698_Lu14-5765.clw

# /r /w123 /lp

#

# .\Lu6-41637_Lu12-11761_Lu12-11698_Lu14-5765.clw: 4 sequences:

# CLUSTAL W (1.81) multiple sequence alignment

#

# Options and features: 4 DNA sequences, SILENT SITES only,

# 2331 aligned bases, 83 polymorphisms, CLUSTAL format,

# mismatches in fragments not allowed, no groups defined,

# maxSimPairPval=0.05, maxSimGlobPval=0.05, no maxKAPairPval limits,

# no maxKAGlobPval limits, simPvals based on 10,000 permutations,

# global P-values based on BLAST-like global scores,

# fragment limits: minlength=1, minnpolys=2, minscore=2,

# pairwise max lists=2000, starting seed=123,

# sites with indels are skipped,

# no match (replacement) char in source file.

# Sequence names: Lu14-5765 Lu12-11761 Lu6-41637 Lu12-11698

#

# No mismatches in fragments (`gscale'=0)

# Nucleotide codes in data: T(1349) C(1371) A(1792) G(1663)

# Other(3149) CG/TCAG ratio: 49.13%

# Non-nucleotide characters in data: -(2DH)(n=3149)

# 1322 sites with missing data or unrecognized characters.

# No sites with all gap or unrecognized characters.

# 407 polymorphic and 602 monomorphic sites with no indels.

# 453 codon positions with indels or missing data.

# 173 amino-acid polymorphic codon positions with no indels.

# 83 silent codon polymorphisms to be permuted.

# CODON POSITIONS with unknown or missing data are excluded.

# The silent sites at 151 amino-acid monomorphic codon positions are

# 0-degen (1) 2-degen (97) 3-degen (0) 4-degen (36) irreg (17)

# The silent polymorphic sites from these codon positions are

# 0-degen (0) 2-degen (54) 3-degen (0) 4-degen (16) irreg (13)

# Codon usage for 3-fold degenerate codons: (Ile) ATT(5) ATC(6) ATA(1)

#

# The starting random number seed is 123.

# Simulated P-values are based on 10,000 permutations.

# Maximum BLAST-like scores:

# Inner Max Sim S.D.s above S.D. of

# frags Score P-value sim. mean sims

# SCORE 2.808 0.1205 1.09 1.0822

# OuterSeq

# frags 1.262 0.3762 0.25 1.1464

#

# Global lists: no I and no O significant fragments

# Pairwise lists: 1 I and no O significant fragments

# where I means inner pair, O is outer seq, A is outer pair, and

# G means outer group (fragments).

# Global P-values are based on BLAST-like global scores.

#

# Fragment offsets and lengths are in nucleotides.

# Bases other than `TCAGU' are treated as indels

# for the purpose of generating polymorphisms.

#

#

# Global inner fragments (83 polymorphisms, 2331 aligned bases):

# (Inner fragments are runs of matching sites.)

# (Sim and BC KA P-values are corrected for multiple comparisons.)

# (Fragments are listed only if Sim P-value <= 0.05.)

# See earlier in the file for the full restrictions on fragments.

#

#

# No inner fragments listed.

#

#

# Global outer-sequence fragments (83 polymorphisms, 2331 aligned bases):

# (Outer-sequence fragments are runs of unique sites.)

# (Sim and BC KA P-values are corrected for multiple comparisons.)

# (Fragments are listed only if Sim P-value <= 0.05.)

# See earlier in the file for the full restrictions on fragments.

#

#

# No outer-sequence fragments listed.

#

#

# Pairwise inner fragments (83 polymorphisms, 2331 aligned bases):

# (Inner fragments are runs of matching sites.)

# (P-values are not corrected for multiple comparisons.)

# (Fragments are listed only if Sim P-value <= 0.05.)

# See earlier in the file for the full restrictions on fragments.

#

# KA Pvalues are NOT corrected for multiple pairwise comparisons.

# Multiply by 6 for Bonferroni-corrected KA Pvalues.

# Num Poly is the number of polymorphic sites in the fragment.

# Num Dif is the number of mismatches within the fragment.

# Tot Difs is the total number of mismatches between two sequences.

# MisM Pen is the penalty per mismatch for these two sequences.

#

# Seq Sim KA Aligned Offsets Num Num Tot MisM

# Names Pvalue Pvalue Begin End Len Poly Dif Difs Pen.

PI Lu14-5765;Lu6-41637 0.0421 0.05852 655 750 96 9 0 43 None

#

# One inner fragment listed.

#

#

# Pairwise outer-sequence fragments (83 polymorphisms, 2331 aligned bases):

# (Outer-sequence fragments are runs of unique sites.)

# (P-values are not corrected for multiple comparisons.)

# (Fragments are listed only if Sim P-value <= 0.05.)

# See earlier in the file for the full restrictions on fragments.

#

# No outer-sequence fragments listed.

#

Group 7

CLUSTAL W (1.81) multiple sequence alignment

Lu8-3343 ATGGCCTCTACTCTCACTATGACCACCATTGCTACCCTATTTGCTCCTGCATGTCCTACT

Lu11-24918 ATGGCTGCAACTTCTTCTCTCGCTGTCACAACTACCCTACTTG------------CTACC

Lu10-34966 ATGGCT---ACTTCCTCTCTGGCTATGACAACTACGTTGCTTG------------CTACC

Lu11-28070 ATGGCT---ACTTCCTCTCTCGCTATGACCACTACCTTGCTTG------------CTACT

***** *** ** * * * **** * *** ****

Lu8-3343 GCTGCTTCTTCGAAAACCAGACCGCCGATGGTAGC-------------------------

Lu11-24918 GTTGGCGCCTCTAAAAATAGGTTGCCCCCGACAGCTTCCGTCTCATGCAA----------

Lu10-34966 GTGGGCGCCTCCAAAACTATATCACCACCTGTTGCT---GTCTCATACAAGGCTTGTGGG

Lu11-28070 GTTGGCGCCTCCAAAACCAGATCACCATCAACAACG---GTCTTATTTAAGACTAATGGA

* * * ** **** * ** *

Lu8-3343 ----------------ATCGT-----------------CGGCA-----------GTTGCT

Lu11-24918 ----------------ATCCTATCC-------------CGGT------------GCTACT

Lu10-34966 AAAAGGGATGGCGGTTATCCTCCCCTCTCTCCTCTTTTCGGTAAAAAAAAGTGGGATGTT

Lu11-28070 AAGAGGGATGCTGGCTATCCTCCTCTTTCTCCTCTTTTTGGTTAAAAGAGAAGGGATTTT

*** * ** * * *

Lu8-3343 CGCGTATCCAACTCCAAAAC----------------------------------------

Lu11-24918 AATGCTGTCTCATACAAAGC----------------------------------------

Lu10-34966 GGC-TATCCTCCTCTTTCTCCTCTTTTTGGTCAGAAAAAGAATGACGCTAGTTATCCTCC

Lu11-28070 GGC-TATCCTCCTCTAAATCCTCTTTTCGGCCAGAAAAAAGGAGATACCGGCTACCCTCC

* * *

Lu8-3343 ----TCTCGTCTTTCCCGCCGAGAAAAATACGAC--------------------------

Lu11-24918 ------------------------------------------------------------

Lu10-34966 CCTTTCTTCTCTTTTCGGCCGAAAAAAGAGAGATGTCGAATATCCTTCCATCTCTCCTCT

Lu11-28070 CTTAAATCCTCTTTTCGGACAGCAAAAGGGAGATACCGGCTACCCTCCTTTAAATCCTCT

Lu8-3343 ---------------------------------------------------------AAC

Lu11-24918 ------------------------------------------------------TCGGAT

Lu10-34966 TCTTGGTCGGGAGAAGAAAGATGCCGGATATCCTCCTCTCTCTCCTCTTTTCGGTCAGAA

Lu11-28070 TTTCGGTGAGAAGAAGGAGGATACCGGCTACCCTTCCTTACATTCCCTTTTTGGCCAAAA

*

Lu8-3343 CAAGTCGTTTGTTGG------TCCTGTCTCTCGA---------------AAGGGCGACGC

Lu11-24918 GAAGGGGGATGCTGGCTATCCTCCCCTCTCTCCACTCTTTGGGCAGAACAATGAGGATGC

Lu10-34966 AAAAGGGGATACCGGCTATCCTCCCCTCTCTCCACTCTTCGGTCAGGAGAAGAGCGATGA

Lu11-28070 GAAGGGGGATACCGGTTTTCCTCCCCTCTCTCCATTATTCGGTCAGGAAAAACGTGATGC

** * * ** *** ****** * ** ** *

Lu8-3343 TAGCCA---CTACCACTGGCCTGTGTTAGGAAAGGGCCATGAGGAGGAGGA---------

Lu11-24918 TGGCTATCCTCCTCTCTCTCCCCTCTTCGG---------TCAGGAGAAGGG---------

Lu10-34966 TGGCTATCCTCCCCTCTCTCCACTCTTCGG---------TCAGGAGAAGGACAATACCGG

Lu11-28070 TGGCTATCCCCCCCTTTCCCCCCTCTTCAG---------TCAGGAAAAGGA---------

* ** * * * ** * ** * * **** ***

Lu8-3343 ------------------------------------------------------------

Lu11-24918 ------------------------------------------------------------

Lu10-34966 ATATCTTCCCCTCTCTTCACTCTTCGGTCAGAAAAAAAGCGATGCTGGCTATCCTCCCCT

Lu11-28070 ------------------------------------------------------------

Lu8-3343 ---------------------------AGATGCGAGC------CTCTTCAACTATTATGT

Lu11-24918 ---------------------------GGATGCTGGCGTTTACGGCTTCATCCCTCACGT

Lu10-34966 CTCCCCACTCTTCGGTCAGGATAATGGCGATGCCGGCTATCCTCCCCTCTCCCCTC---T

Lu11-28070 ---------------------------AGATGCCGGCTATCCTCCCCTCTCCCCTC---T

***** ** * ** * * *

Lu8-3343 GTTCGGA------AAGG-------------------------------------------

Lu11-24918 GTTCGGC------AAGG-------------------------------------------

Lu10-34966 CTTCGGTCAGGAAAAGAGCGATGCCGGTTATCCACCCCTTTCTCCTCTCTTTGGCCAGGA

Lu11-28070 CTTCGGTCAGGAGAAGGGCGATGCC-----------------------------------

***** ***

Lu8-3343 ------------------------------------------------------------

Lu11-24918 ------------------------------------------------------------

Lu10-34966 GGAACGAGATGCTGGCTATCCTCCTCTCTCTCCACTCTTTGGTCAGGATGAGCGCGATGC

Lu11-28070 ------------------------------------------------------------

Lu8-3343 --------------------------CCGGTCAG---GAGGTGGGCGATGCGAGC-----

Lu11-24918 -----------------------------GCCAGGTTGAGAATGGTGATGCTGGCGTTTA

Lu10-34966 TGGCTATCCTCCCCTCTCTCCACTCTTCGGTCAG---GAGAAGAGCGATGCCGGCTATCC

Lu11-28070 -GACTATCCTCCCCTCTCCCCTCTCTTCGGTCAG---GAGAAGGGCGATGCCGGCTATCC

* *** *** * ***** **

Lu8-3343 -CTCTTCAACTATTATGTGTTCGGAAAG--------------------------------

Lu11-24918 CGGCTTCATCCCTCACGTGTTCGGCAAG--------------------------------

Lu10-34966 ACCCCTCTCCCCTC---TCTTCGGTCAGGAGAAACGAGATGCTGGCTATCCTCCTCTCTC

Lu11-28070 TCCCCTCTCCCCTC---TTTTCGGTAAG--------------------------------

* ** * * * ***** **

Lu8-3343 ----------GGCCATGAGGAG--------------------------------------

Lu11-24918 ----------GGCCAGGTTGAG--------------------------------------

Lu10-34966 TCCACTTTTTGGTCAGGATGAGCATGATGCTGGTTACCCTCCCCTCTCCCCCCTCTACGG

Lu11-28070 -------------------GAG--------------------------------------

***

Lu8-3343 ----------GAGGGAGATGCGAG------------------------------------

Lu11-24918 ----------AATGGTGATGCTGG------------------------------------

Lu10-34966 AAGACAGCCAGAGGGAGATGCGGGTTGTGTTTCTCCCGATTTTCATATGATATTTGGCAA

Lu11-28070 ----------AAAGGCGATGCTGGATACAACGGTC---------------TTTTTGGCAA

* ** ***** *

Lu8-3343 -------------CCTCTTCAACTATTATGTG----------------------------

Lu11-24918 -------CGTTTACGGCTTCATCCCTCACGTG----------------------------

Lu10-34966 CGATGCTGGCTGGCAGTGTATTTTTTCTTATGACAAG-----------------------

Lu11-28070 AGATGCTGGCTGGCAGTGTATTTTTTCATATGACAAAACTGGCGATGCTGGATACAATGG

* * * **

Lu8-3343 ----TTCGGAAAG---GCCGGCCAG---------------------GAGGAGGGCGATGC

Lu11-24918 ----TTCGGCAAG------GGTCAG------------------GTTGAGAATGGCGATGC

Lu10-34966 ----ACTGGCAAT---GCTGGTTGGCAGTGTATTTTTTCCTATGACAAGACTGGCGATGC

Lu11-28070 TCTTTTTGGCAAAGATGCTGGCTGGCAGTGTATTTTTTCCTATGACAAGACTGACGATGC

** ** ** * ** * ******

Lu8-3343 TAGCCTCTTCAACT---------------------------------------------A

Lu11-24918 TGGCGTTTACGGCTTCATCCC---------------------------------------

Lu10-34966 TGGCAACAACGGTCTTTTTGCAAAGCGTCAGGATGGCGAGGTTGGCGATGCTGGCTATAA

Lu11-28070 TGGCTACAACGGTCTTTTTGGCAAA---------------------GATGCTAGCTGGCA

* ** *

Lu8-3343 TTATGTGTTCGGCAAGGGCCATGAG---GAGGAGGGCGATGCTAGCCTCTTCAACTA---

Lu11-24918 TCACGTGTTCGGAAAGGGTCAGGTT---GAGAATGGCGATGCTGGCGTTTACGGCTTCAT

Lu10-34966 CGGTCTTTTCGCAAAGCGTCAGGATGGCGAGGTTGGCGATGCTGGCTATAACGGTCT---

Lu11-28070 GTGTATTTTT------TGCTATGAC---AAGGTTGGCGATGCTGGCTACAACGGTCT---

* ** * * * ** ********* ** *

Lu8-3343 ---TTATGTGTTCGGCAAGGCTGGTCAG------GAGGTGGACGATGCGAGCCTCTTCAA

Lu11-24918 CCCTCACGTGTTCGGAAAG---GGTCAGGTT---GAGAATGGCGATGCCGGCGTTTACGG

Lu10-34966 ---T------TTCGCAAAG---CGTCAGGATGGCGAGGTTGGCGATGCTGGCTATAACGG

Lu11-28070 ---T------TTTGCAAAG---TGTCATGATGGCGAGGTTGGCGATACCGGCTATTACGG

* ** * *** **** *** * **** * ** *

Lu8-3343 CTA------TTATGTGTTCTGCAAGGGCCACGAGGAGGAG---GCCGATGCCAGCCTCTT

Lu11-24918 CTTCATCCCTCACGTGTTCGGAAAGGGTCAGGTTGAGAAA---GGCGATGCCGGCGTTTA

Lu10-34966 TCT------T------TTCGCAAAGCGTCAGGATGGCGAGGTTGGCGATGCTGGCTATAA

Lu11-28070 TCT------T------TTTGCAAAGTGTCATGATGGCGAGGTTGGCGATACCGGCTATTA

* ** *** * ** * * * * **** * **

Lu8-3343 CAACTA------TTATG-------------------------------------------

Lu11-24918 CGGCTTCATCCCTCACG-------------------------------------------

Lu10-34966 CGGTCT------TTTCGCAAAGCGTCAGGATGGCGAGGTTGGCGATGCTGGCTATAACGG

Lu11-28070 CGGTCT------T-----------------------------------------------

* *

Lu8-3343 --TGTTCGGCAAGGGCCATGAGGAGGAGGC---CGATGCTAGCCTCTTCAATT-------

Lu11-24918 --TGTTCGGCAAGGGCCAGGTTGAGAAGGCCGGCGATGCTGGCAGTACTGTTC-------

Lu10-34966 TCTTTTCGCAAAGCGTCAGGATGGCGAGGTTGGCGATGCTGGCTATAACGGTCTTTTCGC

Lu11-28070 ----TTTGCAAAGTGCCATGATGGCGAGGTTGGCGATACCGGCT------ATT-------

** * *** * ** * * *** **** * ** *

Lu8-3343 --------------------------------------ATTATGTGTTTGGCAAGGCC--

Lu11-24918 --------------------------------------CCTACTTTCCTATGATATTC--

Lu10-34966 AAAGCGTCAGGATGGCGATGTTGGCGATGCTGGCTATAACCGTCTTTTCGCAAAACGTCA

Lu11-28070 --------------------------------------ACGGTCTTTTTGTAAAGTGTCA

* *

Lu8-3343 -GGTCAAGAGGAGGGCGATGCTAGCCGCTAC----CATTGGCCTTGCTTCGGCAAGACCA

Lu11-24918 -GGCAAGGATGTTGGCTACATTGCTCACCTCATCATATTTGGAAATCGTAG---------

Lu10-34966 GGATGGCGAGGTTGGCGATGCTGGCTATAACGGTCTTTTCGCAAAGCGTCAGGATGGCGA

Lu11-28070 TGATGGCGAGGTTGGCGATACCGGCTATTACGGTCTTTTTGCAAAGTGTCATGATGACGA

* ** * *** * * ** * *

Lu8-3343 T--------------------------------CTAG

Lu11-24918 ---------------------------------CTGA

Lu10-34966 GGTTGGCGATGCTGGCTATAACGGTCTTTTTGCCTAA

Lu11-28070 GGTTGGCGATACCGGCTATTACGGTCTTTTTGCCTAA

**

# Output of GENECONV for sequence file .\Lu10-34966_Lu11-24918_Lu11-28070_Lu8-3343.clw

# GENECONV version 1.81

# Command line: GENECONV .\Lu10-34966_Lu11-24918_Lu11-28070_Lu8-3343.clw

# /r /w123 /lp

#

# .\Lu10-34966_Lu11-24918_Lu11-28070_Lu8-3343.clw: 4 sequences:

# CLUSTAL W (1.81) multiple sequence alignment

#

# Options and features: 4 DNA sequences, SILENT SITES only,

# 1597 aligned bases, 11 polymorphisms, CLUSTAL format,

# mismatches in fragments not allowed, no groups defined,

# maxSimPairPval=0.05, maxSimGlobPval=0.05, no maxKAPairPval limits,

# no maxKAGlobPval limits, simPvals based on 10,000 permutations,

# global P-values based on BLAST-like global scores,

# fragment limits: minlength=1, minnpolys=2, minscore=2,

# pairwise max lists=2000, starting seed=123,

# sites with indels are skipped,

# no match (replacement) char in source file.

# Sequence names: Lu8-3343 Lu11-24918 Lu10-34966 Lu11-28070

#

# No mismatches in fragments (`gscale'=0)

# Nucleotide codes in data: T(1119) C(1052) A(835) G(1071)

# Other(2311) CG/TCAG ratio: 52.07%

# Non-nucleotide characters in data: -(2DH)(n=2311)

# 989 sites with missing data or unrecognized characters.

# No sites with all gap or unrecognized characters.

# 326 polymorphic and 282 monomorphic sites with no indels.

# 360 codon positions with indels or missing data.

# 142 amino-acid polymorphic codon positions with no indels.

# 11 silent codon polymorphisms to be permuted.

# CODON POSITIONS with unknown or missing data are excluded.

# The silent sites at 30 amino-acid monomorphic codon positions are

# 0-degen (1) 2-degen (6) 3-degen (0) 4-degen (13) irreg (10)

# The silent polymorphic sites from these codon positions are

# 0-degen (0) 2-degen (0) 3-degen (0) 4-degen (9) irreg (2)

# Codon usage for 3-fold degenerate codons: (Ile) ATT(5) ATC(0) ATA(2)

#

# Comparisons without any fragments (SILENT SITES):

# Lu11-28070 (outer sequence frags): No sites are unique.

#

# The starting random number seed is 123.

# Simulated P-values are based on 10,000 permutations.

# Maximum BLAST-like scores:

# Inner Max Sim S.D.s above S.D. of

# frags Score P-value sim. mean sims

# SCORE 0.712 0.6766 -0.51 0.5146

# OuterSeq

# frags 1.362 0.1524 1.36 0.5627

#

# Global lists: no I and no O significant fragments

# Pairwise lists: no I and no O significant fragments

# where I means inner pair, O is outer seq, A is outer pair, and

# G means outer group (fragments).

# Global P-values are based on BLAST-like global scores.

#

# Fragment offsets and lengths are in nucleotides.

# Bases other than `TCAGU' are treated as indels

# for the purpose of generating polymorphisms.

#

#

# Global inner fragments (11 polymorphisms, 1597 aligned bases):

# (Inner fragments are runs of matching sites.)

# (Sim and BC KA P-values are corrected for multiple comparisons.)

# (Fragments are listed only if Sim P-value <= 0.05.)

# See earlier in the file for the full restrictions on fragments.

#

#

# No inner fragments listed.

#

#

# Global outer-sequence fragments (11 polymorphisms, 1597 aligned bases):

# (Outer-sequence fragments are runs of unique sites.)

# (Sim and BC KA P-values are corrected for multiple comparisons.)

# (Fragments are listed only if Sim P-value <= 0.05.)

# See earlier in the file for the full restrictions on fragments.

#

#

# No outer-sequence fragments listed.

#

#

# Pairwise inner fragments (11 polymorphisms, 1597 aligned bases):

# (Inner fragments are runs of matching sites.)

# (P-values are not corrected for multiple comparisons.)

# (Fragments are listed only if Sim P-value <= 0.05.)

# See earlier in the file for the full restrictions on fragments.

#

# No inner fragments listed.

#

#

# Pairwise outer-sequence fragments (11 polymorphisms, 1597 aligned bases):

# (Outer-sequence fragments are runs of unique sites.)

# (P-values are not corrected for multiple comparisons.)

# (Fragments are listed only if Sim P-value <= 0.05.)

# See earlier in the file for the full restrictions on fragments.

#

# No outer-sequence fragments listed.

#

Group 8

CLUSTAL W (1.81) multiple sequence alignment

Lu10-38063 ATGGCTACCATTGCCGCC-CGTTTCCCCAAACATACTACTGGTAAGGGAGGCGACCAGAG

Lu9-15288 ATGGCTTCTTCTGCCTTTGCACTTGCTTCCACCT--------TTAGGGTTCCTAC-----

Lu10-38024 ATGGCTACTTCTGCTTTCGCATTTGCTTCCACAT--------TTAGGACTCCCAC-----

****** * *** * ** * ** * * *** * **

Lu10-38063 TGTTAACCCCTGGCGGTTTCCCATCTTTGG-AAAGACATCTCATAACTCTGCTGGAGGTG

Lu9-15288 ----AGCCCCTACCGTTTCCTTGCCGTCGGCCAAGG---CTGATCACTCCGTCCGCAATT

Lu10-38024 ----AGCCCCTATTGTTTCCCTACCGTCAGCCAAGA----ACACTACTCCGGCGGCGGCT

* ***** * ** * * * * *** * **** * *

Lu10-38063 ACCATGGCTACGTCTTACCCTGCTTTGCAGAGGCAAATAACTCTATTGGCGGTGAC----

Lu9-15288 G-----GCC-TGTC---------TTCGGAAAGGCTGATCACTCCGTC---CGCAATTGGC

Lu10-38024 ACCGTTGCC-CGTCGTTTCC-------------CCAATCATTCGGCCAAACGTACTCTAC

** *** * ** * ** *

Lu10-38063 -----------AGGAGTGTTAACC-CC-----TGACGGTTTCCCATCTTCGGAAAGACAT

Lu9-15288 CTGTCTTCGGAAAGGCTGATCACT-CCGTCCGCAAT--TGGCCTGTCTTCGGAAAGG---

Lu10-38024 C-------------GATGCTGGCTACCGTCGGCGATGGTGGCC--TTTTC----------

** * * ** * * ** * ***

Lu10-38063 CTCATAACTCTCCTGGCGGTGACAGTAGTGGGAACGTCCTACCCTGCTTTGCAAA---AG

Lu9-15288 CTGATAACTCCGTCAGCAAT------------------TGGCCTGTCTTCGGAAAGGCAG

Lu10-38024 --------------------------------------CACCACTTCTTTGGAAA---AG

* *** * *** **

Lu10-38063 CCG---ATAACTCTACTGGAGACTGCGATGGTGGCTACGTCCTTACCTGCTTTGCAAAAG

Lu9-15288 CTGATGATAACTCTACGAGAG---GCGATAGTGGCTACGTTGTGCCCTGCTTCGGAAAGG

Lu10-38024 TTGATAATAACTCTACCGTCG---GCGATGATGG---TGTTTTTACCTTCTTTGGAAAGG

* ********** * ***** *** ** * *** *** * *** *

Lu10-38063 CCGAGAACT---------------CTACCGGAGGCGGCGATGCTGGCTATGTTCTGCCCT

Lu9-15288 CTGATAATTCCCACCTCCATTGGCCTGTC---TTCGGAAAGGCTGATAAC-TCCCACCTC

Lu10-38024 CTAAAAATG------------ATTTTACC---GTCGGCGATGGTGGTTTT-TTCCACTAT

* * ** * * *** * * ** * * *

Lu10-38063 ----------GCTTCGGAAAAGCCGATAACTCTACC---------------------AGA

Lu9-15288 CATTGGCCTGTCTTCGGAAAGGCTGATAACTCCGTTCGCAATTGGCCTGTCTTCGGAAAG

Lu10-38024 ------------TTTGGAAAGATTGAAA--------------------------------

** ***** ** *

Lu10-38063 GGCGGCGATGCTGGCTATGTTCTGTCCTGTTTCGGAA---AAGCCGATAACTGTAC----

Lu9-15288 GCTAATAACTCCCACCTCCATTGGCCTGTCTTCGGAAAGGAAGCTGATAACTCCGTCCGC

Lu10-38024 ----------------------------------------------ATAACTCTATC---

******

Lu10-38063 --------------------GAGAGGCGGCGATGATGGCTATATTCTGCCTTGCTTCGGA

Lu9-15288 AATTGGCCTGTCTTCGGAAAGGAAGCTGATAACTCCGTCTGCAATTGGCCTGTCTTCGGA

Lu10-38024 ------------------------GTTGACTACGATGAAGGCATTTTTACCTTTTTTGGA

* * * * * * * ** ***

Lu10-38063 AAAGC---CGATAACTCTACCGCAGGGGGCGATGGTGGCTACGTCCTGCCGTGCTTCGGA

Lu9-15288 AAGGCAACTGATAACTCTATG---GGGGGCGATAGTGGCTACGTTGTGCCGTGCTTCGGA

Lu10-38024 AAGGTTGAAAATGACTCTACC---GTCTGCGATGATGGCT---TTTTCCACTACTTTGGA

** * ** ****** * ***** ***** * * * * *** ***

Lu10-38063 ATTGCCG------------------------------------------ATAACTCTACC

Lu9-15288 AAGGCTGATAACTCCCACCTCCATTGGCCTGTCTTCGGAAAGGAAGCTGATAACTCTA--

Lu10-38024 AACACTGATA---------------------------------------ATAACTCTA--

* * * *********

Lu10-38063 GCAGGCGGCGATGGTGGCTACGTCCGAACCTGCTCTTCTTAA

Lu9-15288 -CGGGAGGCGATAGTGGCTACGTTGTGCCCTGCTTCGGCTGA

Lu10-38024 -AAGGAGTTGATGGTGGTATTGGAAAGGCCGATAATAACTAG

** * *** **** * ** *

# Output of GENECONV for sequence file .\Lu9-15288_Lu10-38024_Lu10-38063.clw

# GENECONV version 1.81

# Command line: GENECONV .\Lu9-15288_Lu10-38024_Lu10-38063.clw /r /w123

# /lp

#

# .\Lu9-15288_Lu10-38024_Lu10-38063.clw: 3 sequences:

# CLUSTAL W (1.81) multiple sequence alignment

#

# Options and features: 3 DNA sequences, SILENT SITES only,

# 762 aligned bases, 16 polymorphisms, CLUSTAL format,

# mismatches in fragments not allowed, no groups defined,

# maxSimPairPval=0.05, maxSimGlobPval=0.05, no maxKAPairPval limits,

# no maxKAGlobPval limits, simPvals based on 10,000 permutations,

# global P-values based on BLAST-like global scores,

# fragment limits: minlength=1, minnpolys=2, minscore=2,

# pairwise max lists=2000, starting seed=123,

# sites with indels are skipped,

# no match (replacement) char in source file.

# Sequence names: Lu10-38063 Lu9-15288 Lu10-38024

#

# No mismatches in fragments (`gscale'=0)

# Nucleotide codes in data: T(481) C(470) A(384) G(441) Other(510)

# CG/TCAG ratio: 51.30%

# Non-nucleotide characters in data: -(2DH)(n=510)

# 330 sites with missing data or unrecognized characters.

# No sites with all gap or unrecognized characters.

# 213 polymorphic and 219 monomorphic sites with no indels.

# 129 codon positions with indels or missing data.

# 90 amino-acid polymorphic codon positions with no indels.

# 16 silent codon polymorphisms to be permuted.

# CODON POSITIONS with unknown or missing data are excluded.

# The silent sites at 35 amino-acid monomorphic codon positions are

# 0-degen (1) 2-degen (9) 3-degen (2) 4-degen (18) irreg (5)

# The silent polymorphic sites from these codon positions are

# 0-degen (0) 2-degen (5) 3-degen (1) 4-degen (6) irreg (4)

# Codon usage for 3-fold degenerate codons: (Ile) ATT(9) ATC(4) ATA(8)

#

# The starting random number seed is 123.

# Simulated P-values are based on 10,000 permutations.

# Maximum BLAST-like scores:

# Inner Max Sim S.D.s above S.D. of

# frags Score P-value sim. mean sims

# SCORE -0.341 0.9916 -1.13 0.7894

# OuterSeq

# frags 0.000 0.9599 -1.07 0.7288

#

# Global lists: no I and no O significant fragments

# Pairwise lists: no I and no O significant fragments

# where I means inner pair, O is outer seq, A is outer pair, and

# G means outer group (fragments).

# Global P-values are based on BLAST-like global scores.

#

# Fragment offsets and lengths are in nucleotides.

# Bases other than `TCAGU' are treated as indels

# for the purpose of generating polymorphisms.

#

#

# Global inner fragments (16 polymorphisms, 762 aligned bases):

# (Inner fragments are runs of matching sites.)

# (Sim and BC KA P-values are corrected for multiple comparisons.)

# (Fragments are listed only if Sim P-value <= 0.05.)

# See earlier in the file for the full restrictions on fragments.

#

#

# No inner fragments listed.

#

#

# Global outer-sequence fragments (16 polymorphisms, 762 aligned bases):

# (Outer-sequence fragments are runs of unique sites.)

# (Sim and BC KA P-values are corrected for multiple comparisons.)

# (Fragments are listed only if Sim P-value <= 0.05.)

# See earlier in the file for the full restrictions on fragments.

#

#

# No outer-sequence fragments listed.

#

#

# Pairwise inner fragments (16 polymorphisms, 762 aligned bases):

# (Inner fragments are runs of matching sites.)

# (P-values are not corrected for multiple comparisons.)

# (Fragments are listed only if Sim P-value <= 0.05.)

# See earlier in the file for the full restrictions on fragments.

#

# No inner fragments listed.

#

#

# Pairwise outer-sequence fragments (16 polymorphisms, 762 aligned bases):

# (Outer-sequence fragments are runs of unique sites.)

# (P-values are not corrected for multiple comparisons.)

# (Fragments are listed only if Sim P-value <= 0.05.)

# See earlier in the file for the full restrictions on fragments.

#

# No outer-sequence fragments listed.

#
