# Supplementary material for: In silico analysis reveals a multi-dimensional model of adaptive evolution in the flax orbitide-related precursor protein family
Source: Front Plant Sci. 2026 Jun 30;17:1824173. doi: 10.3389/fpls.2026.1824173 (PMC13365257; doi:10.3389/fpls.2026.1824173)
Supplement: Supplementary Table 1 — Repeat sections of 30 proteins. [file Supplementaryfile1.zip › Figure S1_similarity matrices of 8 paralogous groups.pptx]

## Slide 1
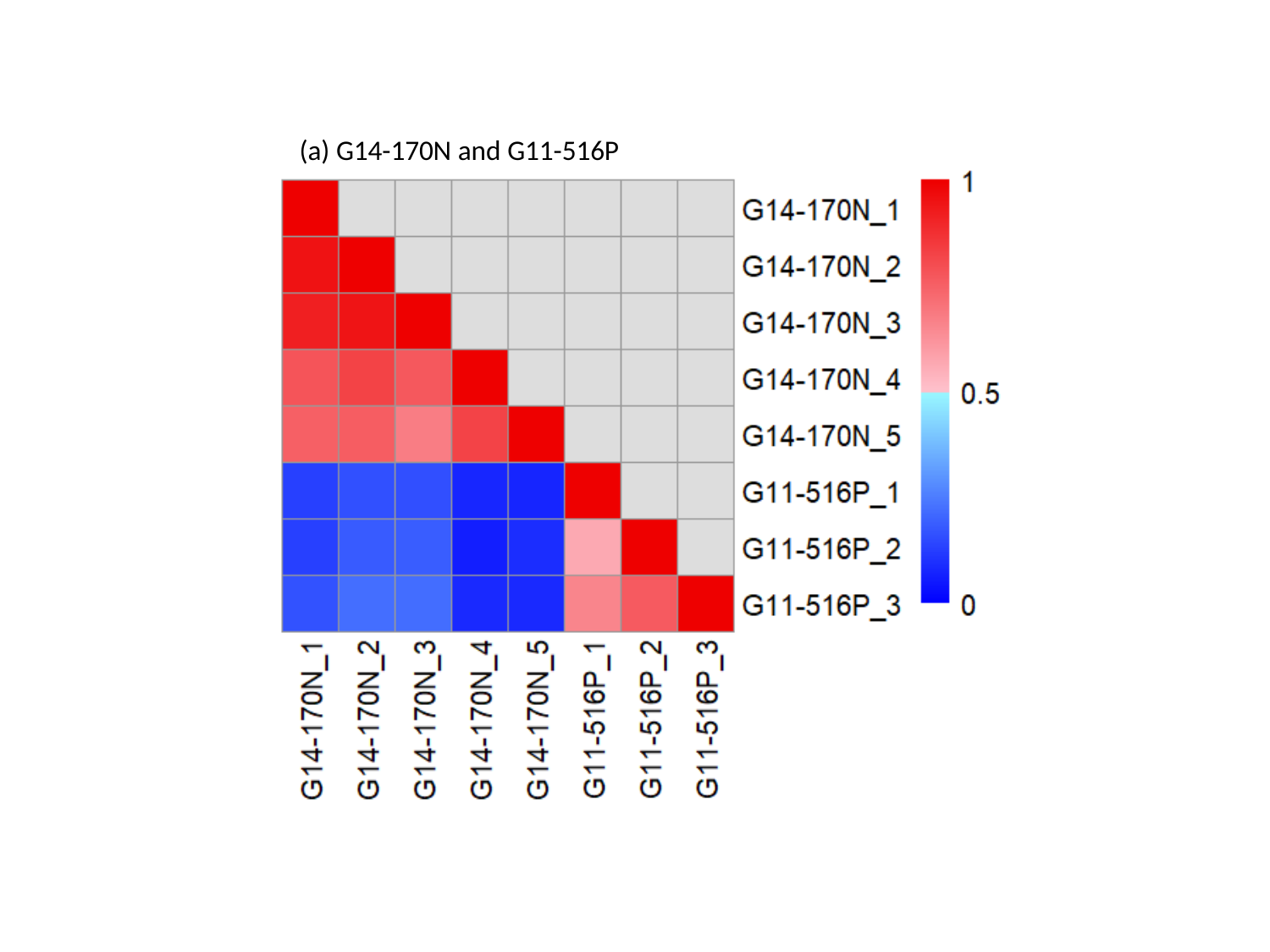

(a) G14-170N and G11-516P

## Slide 2
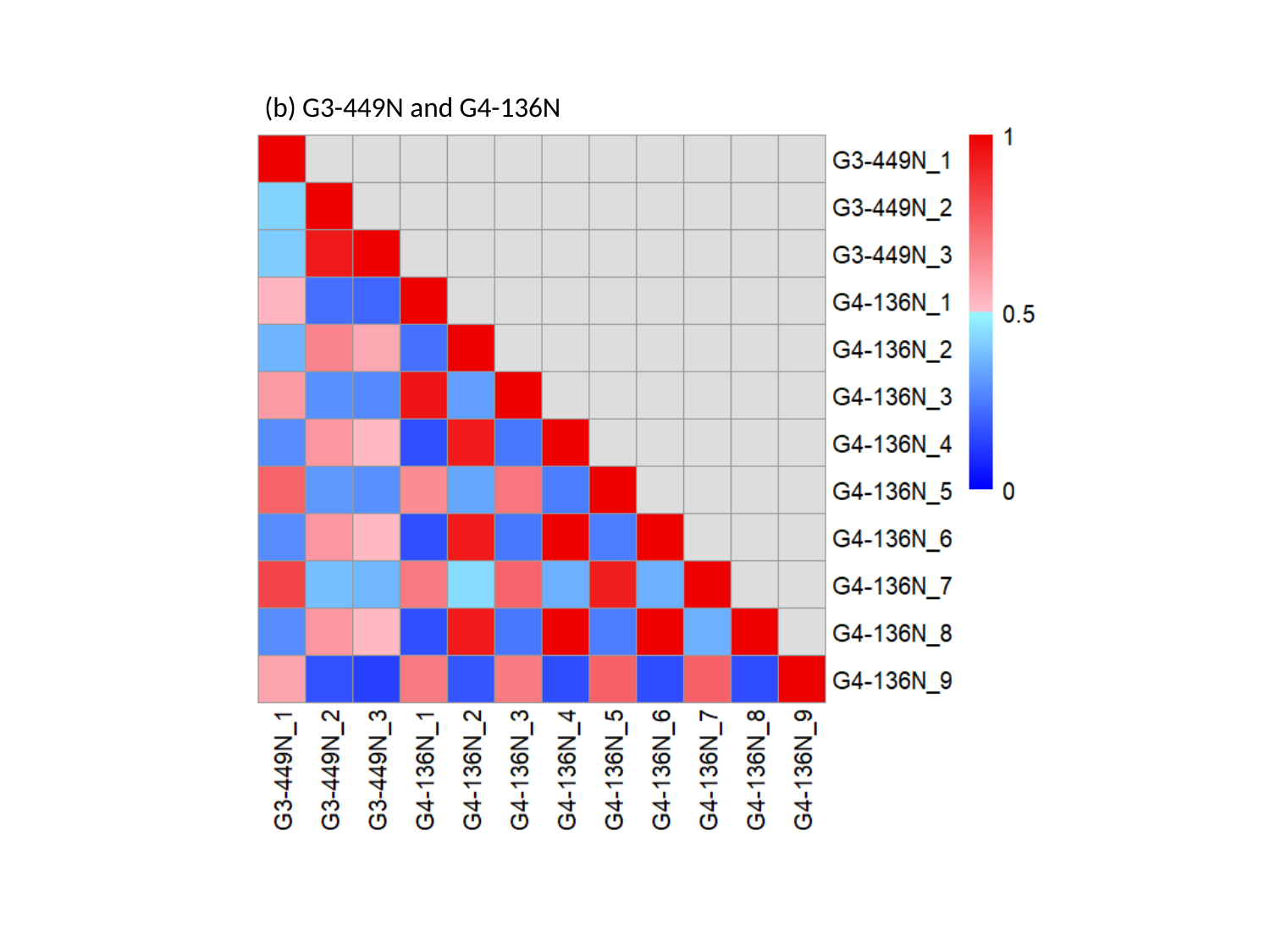

(b) G3-449N and G4-136N

## Slide 3
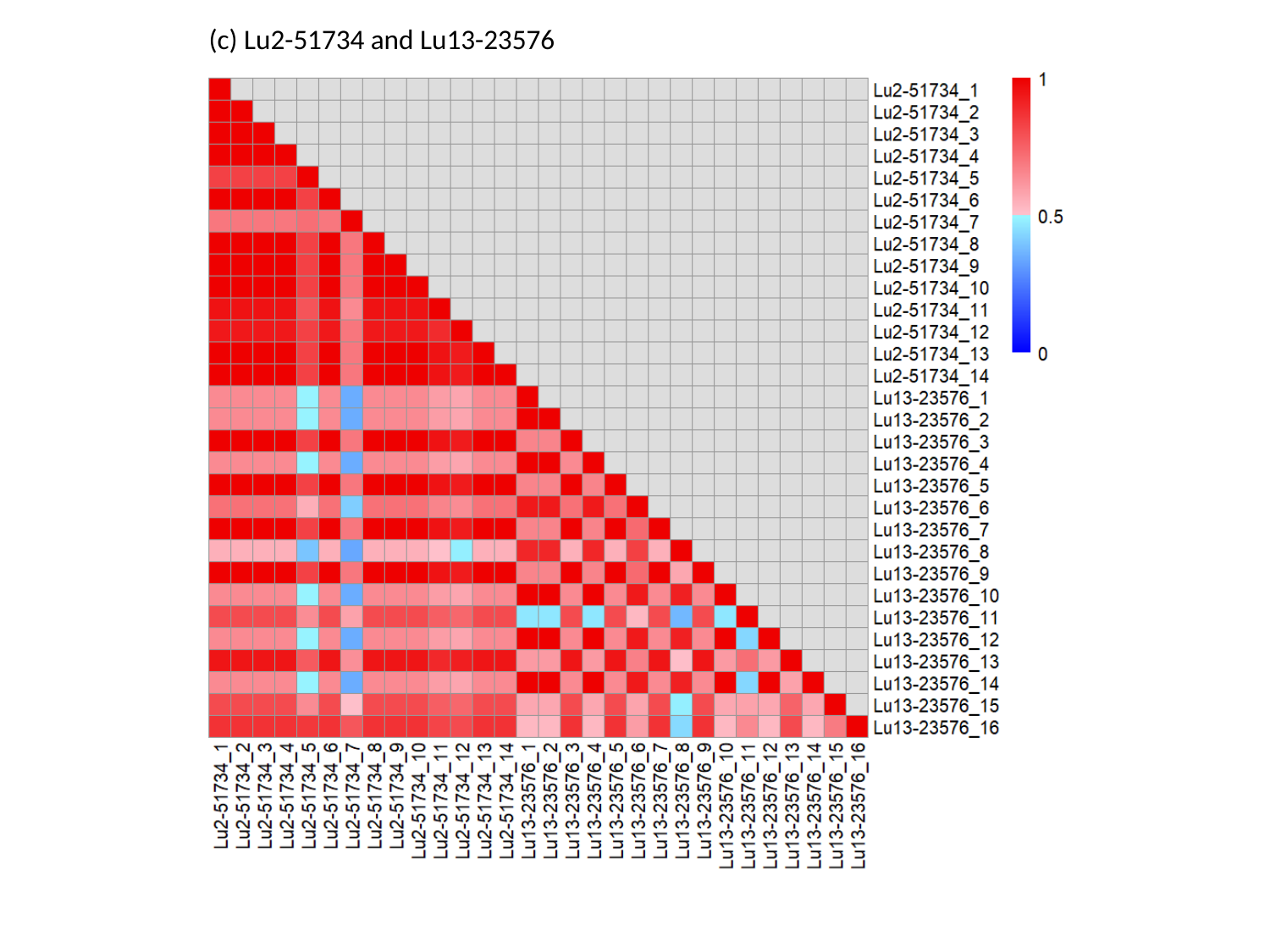

(c) Lu2-51734 and Lu13-23576

## Slide 4
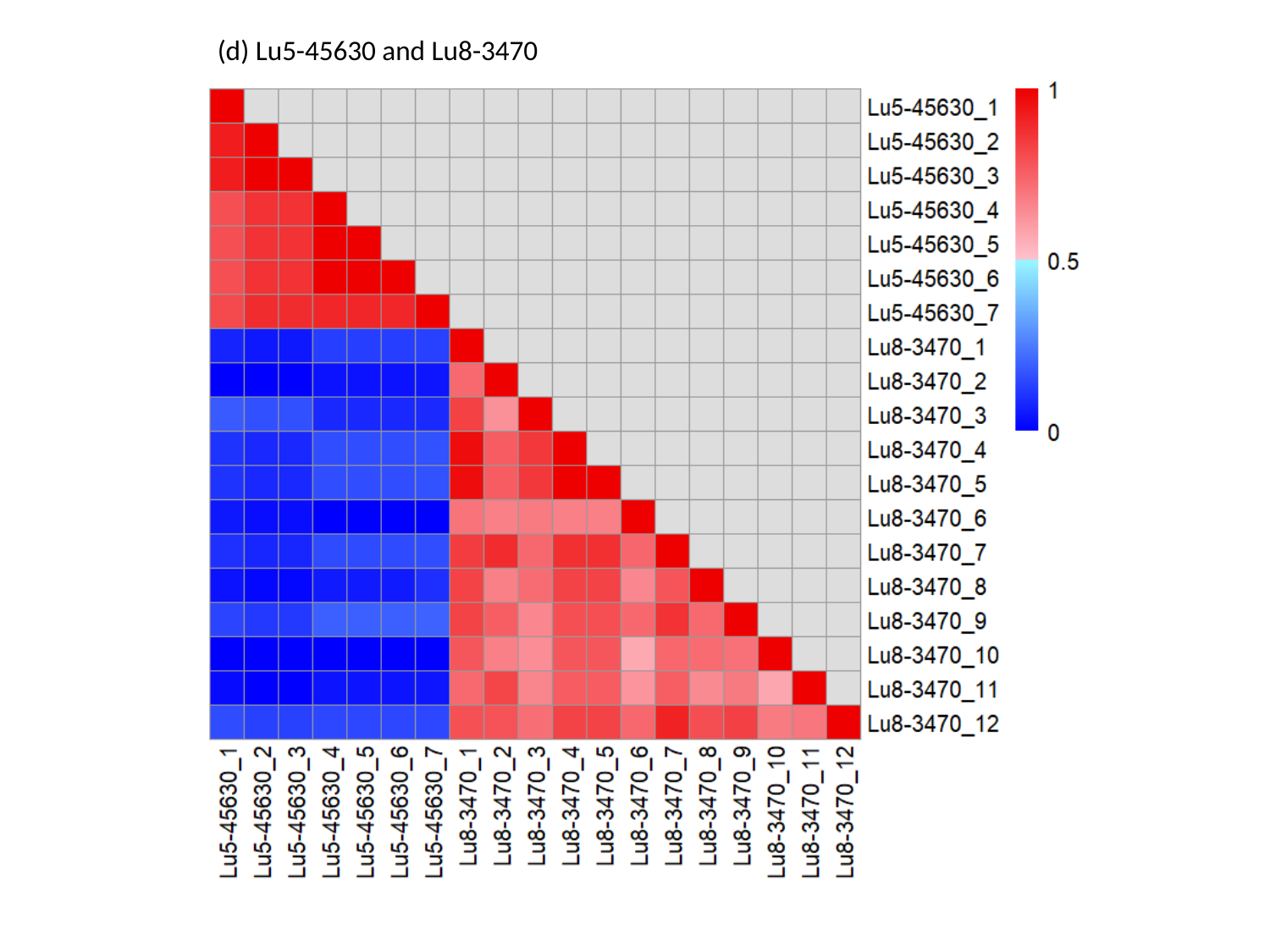

(d) Lu5-45630 and Lu8-3470

## Slide 5
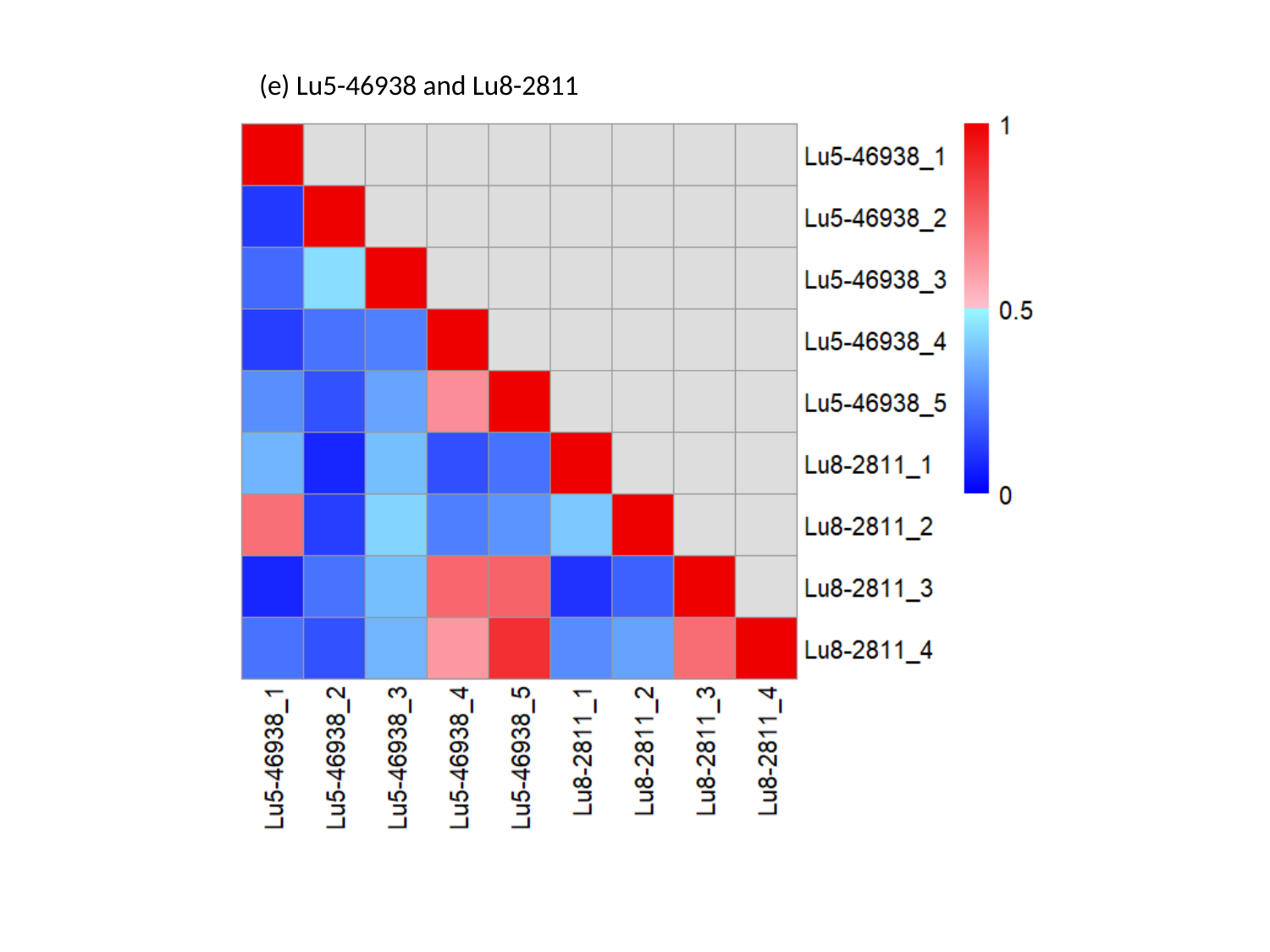

(e) Lu5-46938 and Lu8-2811

## Slide 6
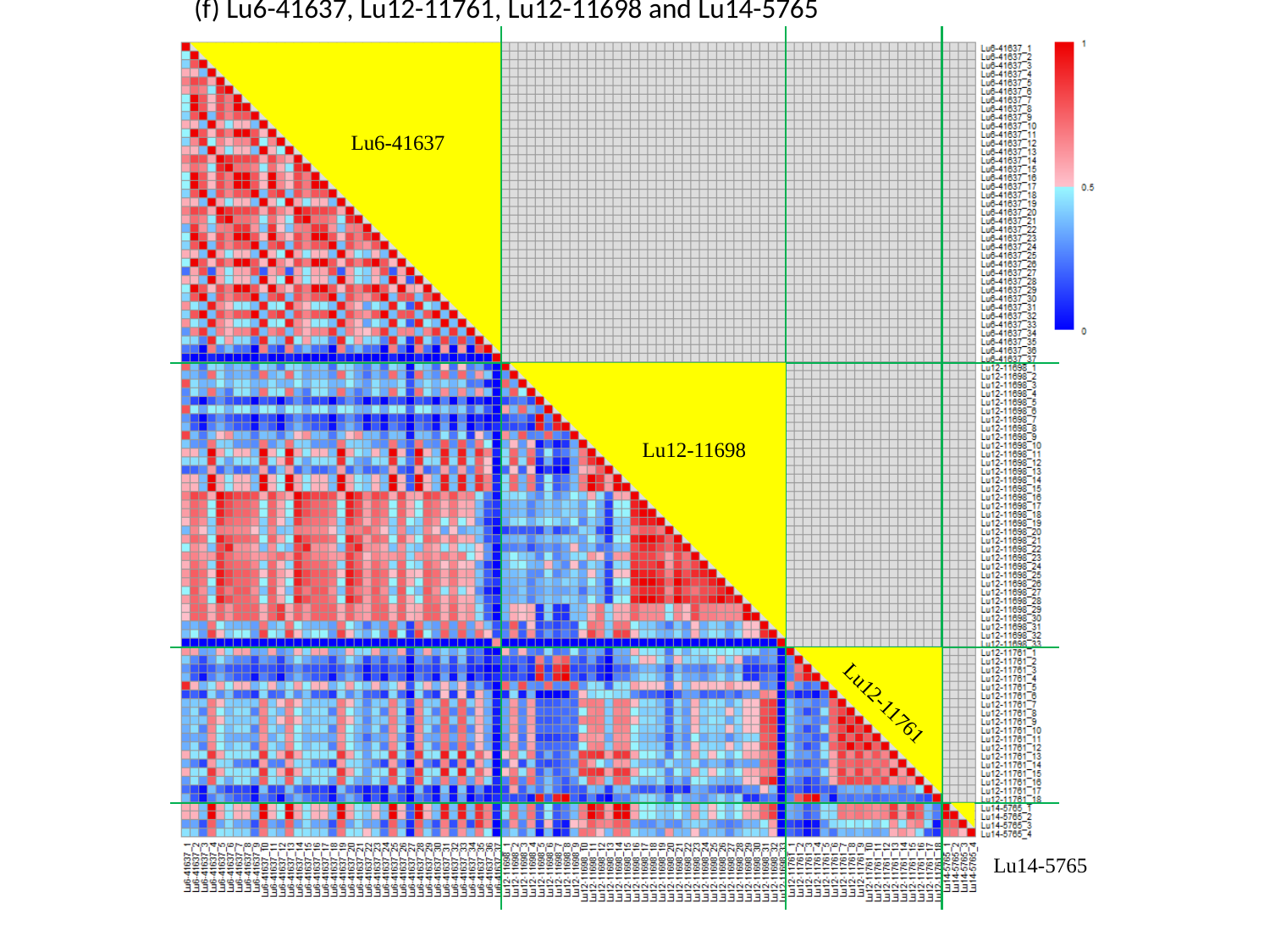

(f) Lu6-41637, Lu12-11761, Lu12-11698 and Lu14-5765
Lu6-41637
Lu12-11698
Lu12-11761
Lu14-5765

## Slide 7
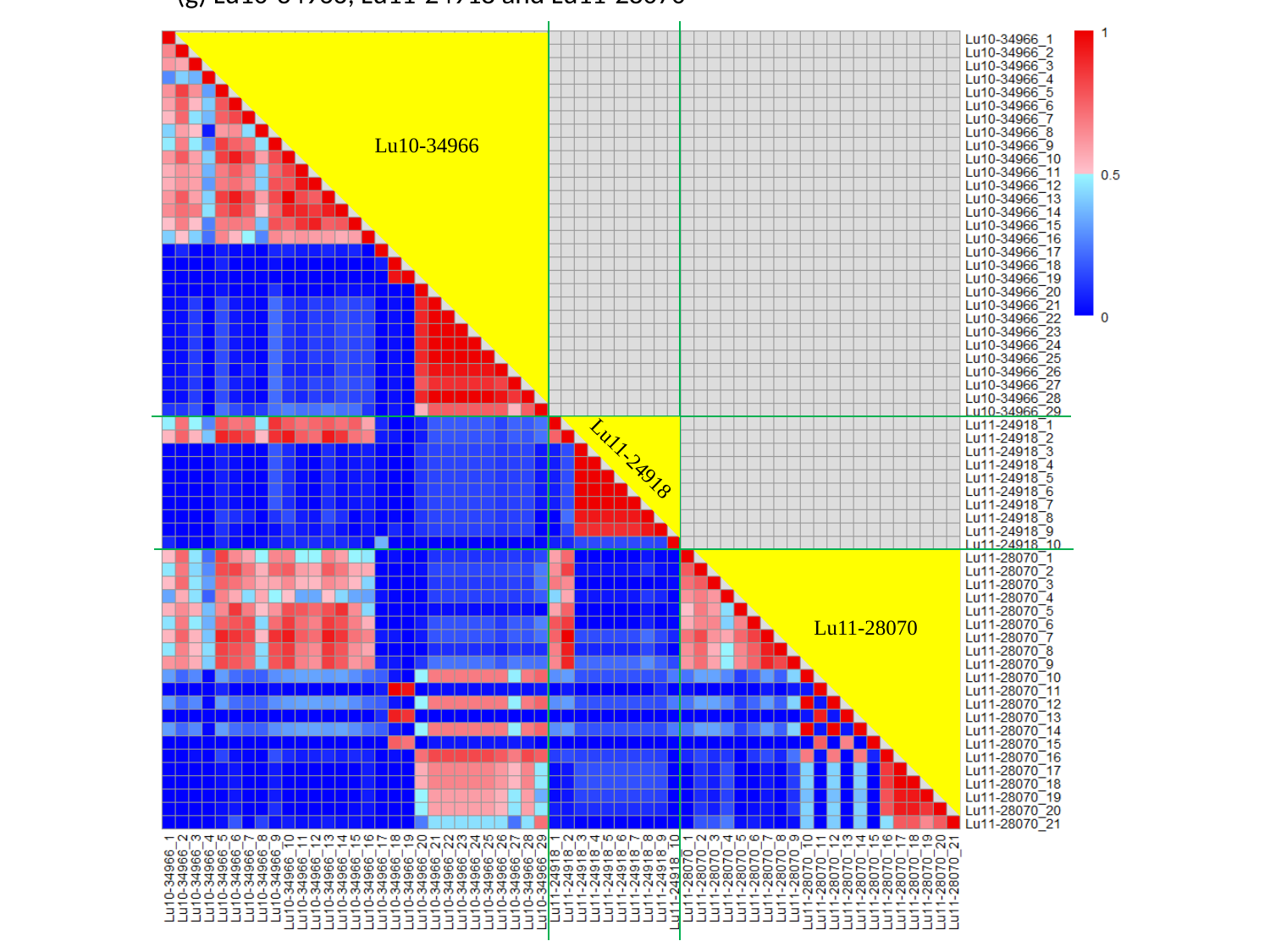

(g) Lu10-34966, Lu11-24918 and Lu11-28070
Lu10-34966
Lu11-24918
Lu11-28070

## Slide 8
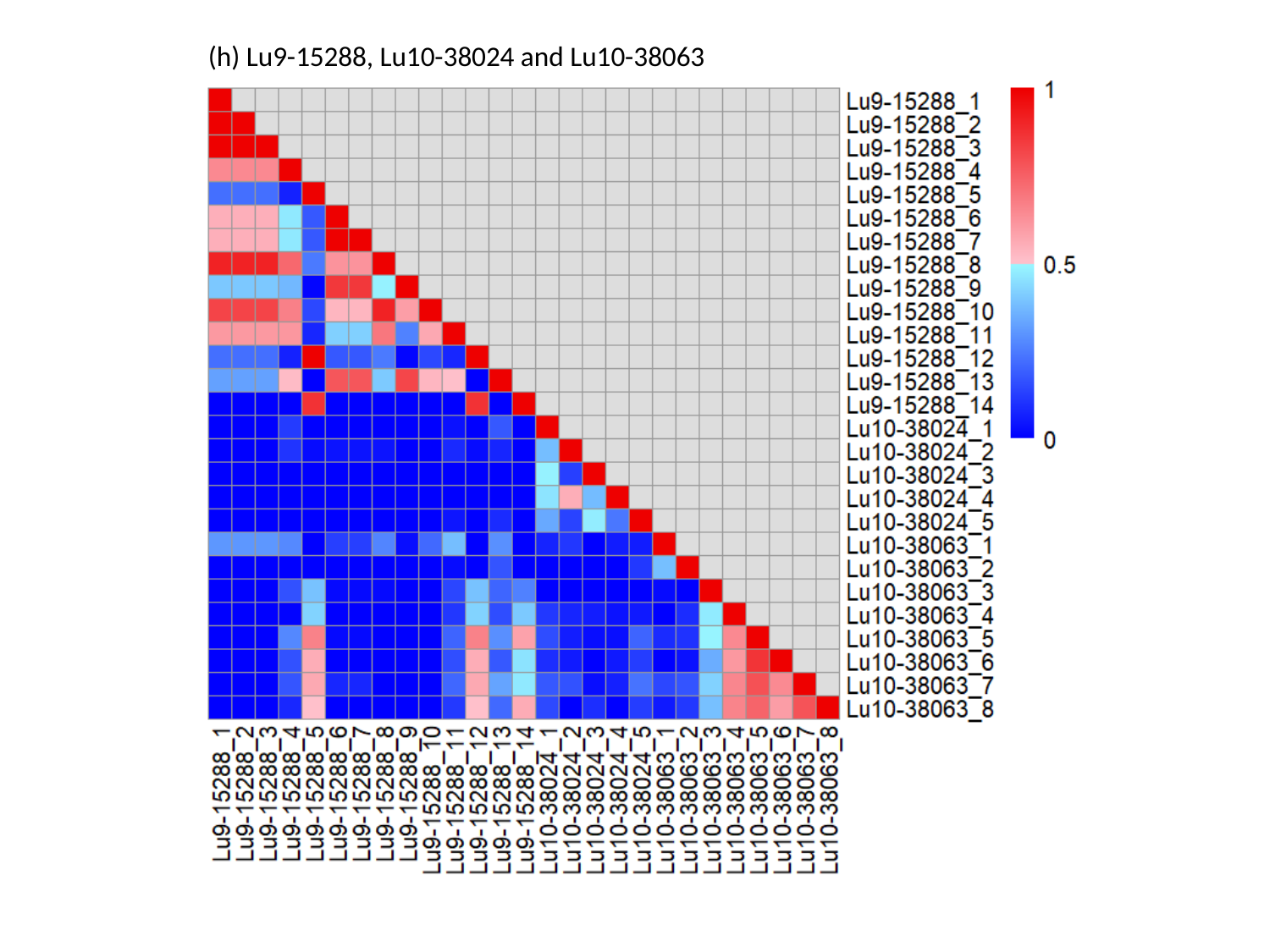

(h) Lu9-15288, Lu10-38024 and Lu10-38063
