# Supplementary material for: In silico analysis reveals a multi-dimensional model of adaptive evolution in the flax orbitide-related precursor protein family
Source: Front Plant Sci. 2026 Jun 30;17:1824173. doi: 10.3389/fpls.2026.1824173 (PMC13365257; doi:10.3389/fpls.2026.1824173)
Supplement: Supplementary Table 1 — Repeat sections of 30 proteins. [file Supplementaryfile1.zip › Table S3_Subcellular localizations of 30 proteins.docx]

Table S3. Subcellular localizations of 30 proteins

| Protein | Prediction | OTHER | SP | mTP | cTP | luTP | Cleavage site (CS) | CS Position | Probability |
| --- | --- | --- | --- | --- | --- | --- | --- | --- | --- |
| G11-516P | cTP | 0.003 | 0.017 | 0.002 | 0.974 | 0.003 | VKA-AA | 51-52 | 0.28 |
| G14-170N | cTP | 0.001 | 0.015 | 0.001 | 0.983 | 0.000 | VKA-VV | 50-51 | 0.70 |
| G11-514P | cTP | 0.060 | 0.008 | 0.000 | 0.918 | 0.014 | VKA-AV | 41-42 | 0.50 |
| G3-449N | cTP | 0.001 | 0.000 | 0.000 | 0.989 | 0.011 | LKK-SG | 63-64 | 0.19 |
| G4-136N | cTP | 0.000 | 0.000 | 0.000 | 0.998 | 0.001 | LKA-RK | 34-35 | 0.25 |
| Lu1-17016 | OTHER | 0.993 | 0.002 | 0.006 | 0.000 | 0.000 |  |  |  |
| Lu1-18055 | cTP | 0.005 | 0.000 | 0.000 | 0.991 | 0.004 | VVA-TS | 42-43 | 0.36 |
| Lu1-18070 | cTP | 0.000 | 0.000 | 0.000 | 0.999 | 0.001 | IKS-GS | 48-49 | 0.65 |
| Lu2-51734 | cTP | 0.003 | 0.105 | 0.004 | 0.884 | 0.004 | ISC-HP | 48-49 | 0.19 |
| Lu3-56299 | cTP | 0.001 | 0.001 | 0.000 | 0.996 | 0.001 | HRA-EV | 54-55 | 0.13 |
| Lu5-45630 | SP | 0.000 | 1.000 | 0.000 | 0.000 | 0.000 | VNG-IR | 25-26 | 0.74 |
| Lu5-46938 | OTHER | 0.857 | 0.106 | 0.035 | 0.003 | 0.000 |  |  |  |
| Lu5-47766 | SP | 0.037 | 0.951 | 0.012 | 0.000 | 0.000 | CHG-WG | 33-34 | 0.80 |
| Lu6-41637 | cTP | 0.135 | 0.000 | 0.001 | 0.782 | 0.082 | VSA-AV | 40-41 | 0.52 |
| Lu8-2811 | OTHER | 0.959 | 0.027 | 0.014 | 0.001 | 0.000 |  |  |  |
| Lu8-3343 | cTP | 0.000 | 0.010 | 0.000 | 0.989 | 0.001 | AVA-RV | 37-38 | 0.20 |
| Lu8-3470 | OTHER | 0.820 | 0.175 | 0.005 | 0.000 | 0.000 |  |  |  |
| Lu9-15288 | cTP | 0.004 | 0.001 | 0.001 | 0.982 | 0.011 | PVF-GK | 50-51 | 0.10 |
| Lu10-34966 | cTP | 0.042 | 0.068 | 0.025 | 0.845 | 0.019 | KAC-GK | 33-34 | 0.27 |
| Lu10-38024 | cTP | 0.000 | 0.000 | 0.000 | 0.991 | 0.009 | MLA-TV | 52-53 | 0.42 |
| Lu10-38063 | OTHER | 0.620 | 0.298 | 0.003 | 0.076 | 0.003 |  |  |  |
| Lu11-24918 | cTP | 0.000 | 0.028 | 0.002 | 0.961 | 0.009 | ARM-KG | 48-49 | 0.20 |
| Lu11-26217 | cTP | 0.002 | 0.000 | 0.000 | 0.907 | 0.091 | ALA-VD | 51-52 | 0.39 |
| Lu11-28070 | cTP | 0.097 | 0.078 | 0.014 | 0.780 | 0.031 | PLF-GQ | 40-41 | 0.11 |
| Lu12-9873 | OTHER | 1.000 | 0.000 | 0.000 | 0.000 | 0.000 |  |  |  |
| Lu12-11698 | cTP | 0.001 | 0.000 | 0.000 | 0.997 | 0.002 | VSA-AV | 46-47 | 0.68 |
| Lu12-11761 | cTP | 0.000 | 0.000 | 0.000 | 0.975 | 0.025 | VSA-VV | 46-47 | 0.43 |
| Lu13-23576 | OTHER | 0.921 | 0.035 | 0.035 | 0.009 | 0.000 |  |  |  |
| Lu14-4819 | cTP | 0.003 | 0.000 | 0.000 | 0.994 | 0.003 | VVA-RG | 42-43 | 0.47 |
| Lu14-5765 | OTHER | 1.000 | 0.000 | 0.000 | 0.000 | 0.000 |  |  |  |

# TargetP 2.0 predicts the presence of N-terminal presequences: secretory signal peptide (SP), mitochondrial transit peptide (mTP), chloroplast transit peptide (cTP), thylakoid luminal transit peptide (lTP) or no sorting signal (other). For sequences predicted to contain an N-terminal presequence a potential cleavage site is also predicted with a probability.
